# Comprehensive risk assessment revealed some physiological indicators responding to various GM-crop consumption

**DOI:** 10.1080/21645698.2025.2603726

**Published:** 2025-12-19

**Authors:** Yangyi Wang, Ruizhe Wu, Ken Cheng, Dunjie Yao, Qiuhan Wu, Haoyue Yong, Haoyu Sun, Xinwei Zhou, Wanjun Zhang, Rong Yuan, Fuying Ma, Libin Zhang, Li Su, Xinhua Zeng

**Affiliations:** a Key Laboratory of Molecular Biophysics of Ministry of Education, College of Life Science and Technology, Huazhong University of Science and Technology Wuhan, China; b School of Environment, Tsinghua University Beijing, China; c Oil Crops Research Institute of the Chinese Academy of Agricultural Sciences/Key Laboratory of Biology and Genetic Improvement of Oil Crops, Ministry of Agriculture Wuhan, China

**Keywords:** Genetically modified crop, risk assessment, physiological indicator, DerSimonian and Laird random-effects model, health status

## Abstract

Genetically modified (GM) crops have been provided as food and feed in over 70 countries in the world. But the concern is persisting on their comprehensive effects on human health status as feedstock. Physiological indicators detected in human beings or animals were explored to assess the health status after GM crop consumption. Here, a mammalian physiological indicators data set with seven metrics containing 25 physiological indicators was constructed by extracting the experimental raw data from the open access research articles published from January 2000 to September 2024 on GM maize, rice, and soybean consumption. To overcome the experimental heterogeneity in disparate model animals, limited animal number in each independent research, and statistical errors caused by different statistical methods, the multi-sourced data correlation analysis with DerSimonian and Laird random-effect model was employed. The result revealed that the concentration of glucose increased after nutritionally changed maize consumption (GLU, *p* < .01), but within the safe reference concentration range; the relative weight of liver increased after non-nutritional GM maize consumption (*p* < .05); the relative weight of kidney was the physiological indicators that significantly increased after nutritionally changed GM rice consumption (*p* < .05). No pathological characterizations in respective organs were reported. The findings indicated no pathological risks from GM crop consumption, though they emphasized the need for continued research into their metabolic and biochemical effects to ensure comprehensive food safety.

## 1. Introduction

Comprehensive risk assessments of genetically modified (GM) crops considering nutritional value and toxicological safety are indispensable to guarantee high-quality wide commercial farming and consumption. With changed stress resistance, higher yields, or rich nutrition, they have been processed into feedstock provided as feed and food in over 70 countries in the world^1^ and hold the superb potential to cope with the global food crisis.^2,3^ Among varieties of GM crops, GM maize might increase yields by 16.4%,^4^ and GM rice (ex. IPA1-Pro10) might increase yields by 15.9% compared to the parent rice,^5^ and GM soybean (ex. ric1a/2a) could produce a yield increase of more than 20% over parent soybean.^6^ Also, based on GM food and feed RASFF notifications, maize accounted for 20.39% in GM feed product, and rice held the leading position in GM food and feed product, and soybean accounted for 10.68% in GM feed product.^1^ These three types of GM crops are particularly noteworthy owing to their significant roles as staple foods and feedstocks. While foraged into feedstock supplies for human beings and mammals, the concerns about the safety of these GM crops and their by-products, especially the effects on human health status, intensified.^2,7–9^ Focused on comprehensive risk assessments, their physiological impacts on human are pivotal in ascertaining the appropriateness.^2,3,8,10^

Existing literature mainly attempted to evaluate the effects on human health after GM crops consumption by experiments on model animals from several main metrics^11–14^ (FDA, 2000).^15^ Rats and mice were widely used in these research under 90-day feeding experiments, respectively, and crab-eating monkeys were used under 52-week feeding experiments.^16–22^ Seven metrics were classified by weight gain, relative organ weight, blood cell concentration, and the index on cardiovascular function, liver function, renal function, and electrolyte function,^22,23^ composing of the indicators which were detected in these three model animals such as concentrations of red blood cell (RBC), serum Cholesterol (CHOL) concentration of cardiovascular function, serum aspartate transaminase (AST) activity and alanine aminotransferase (ALT) activity of liver function, serum creatinine (CRE) concentration of renal function, and serum Na^+^ and K^+^ concentrations of electrolyte function. Independent research has reported that consuming GM maize, rice and soybean had no adverse effect on these mammalian physiological indicators, thus consuming GM maize, rice, and soybean could be considered safe for human.^22,24,25^ However, as an indication of the health status of human beings after consuming GM crops, the integrated effects of consuming GM crops on mammalian health status reflected in the different model animals by the physiological indicators remain undiscovered.

To overcome the experimental heterogeneity in disparate model animals, limited number of animal repeats among each independent research, and statistical errors caused by different statistical methods, a mammalian physiological indicator data set reflecting health status of model animals and human was constructed with qualified literature. The qualified literature assessed GM crop effects using non-GM isogenic parental lines as negative controls in mammalian models, with raw physiological data.

The data set contained seven metrics including 25 physiological indicators. Then, the multi-sourced published raw experimental data were extracted and subjected to a systematic correlation analysis with a DerSimonian and Laird random-effects model. The findings on each indicator and critical subgroups of model animals could help to reveal the integrated and comprehensive influences of GM crop consumption on mammalian health status, and finally indicate the integrated and comprehensive effects of GM crop consumption on human health status.

## 2. Methods

### 2.1. Literature Screening and the Physiological Indicator Data Set Construction

**Figure 1.**
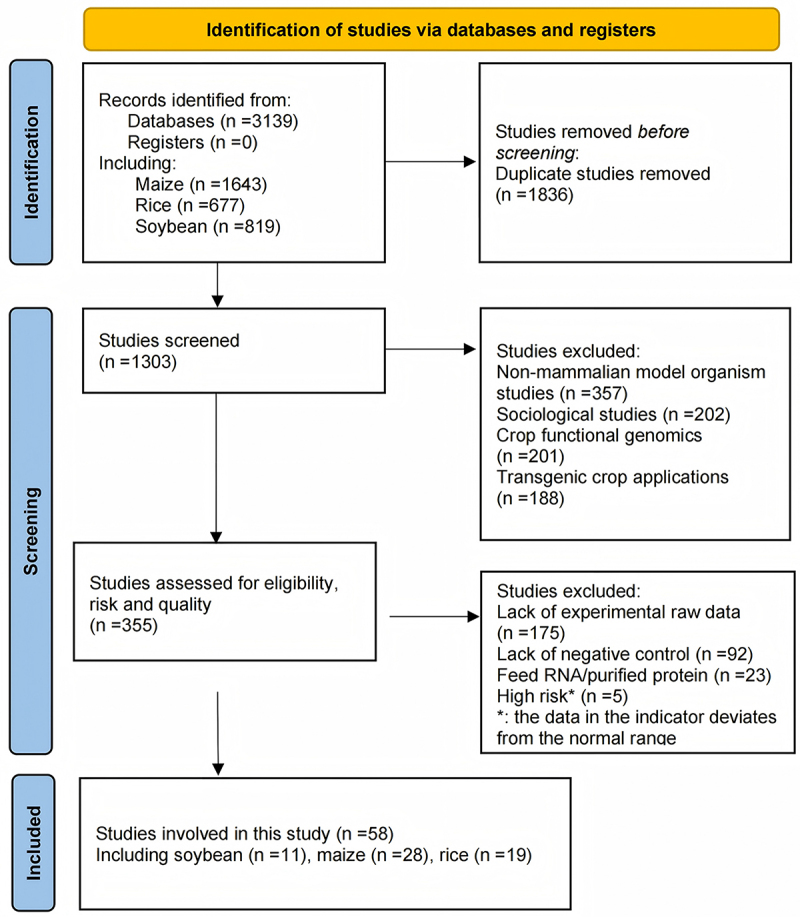
Flowchart for identification, screening and included qualified studies on the health status of mammals after consuming GM crops.

Studies published in open access on the consumption safety of the three GM crops (maize, rice, and soybean) were systematically collected and screened, excluding review papers, interview papers, and letters. Included databases were PubMed, Web of Science, and Scopus, spanning articles published between January 2000 and September 2024. Employed search terms were “gene editing” OR “genetically modified” OR “transgenic,” “maize” OR “corn” OR “Zea mays,” “rice” OR “Oryza sativa,” “soybean” OR “Glycine max” (“feed” OR “feeding” OR “fed”) OR (“animal health” OR “health monitoring”). The screening process was meticulously performed by four independent researchers (Y. Wang, R. Wu, Q. Wu, H. Yong). Sequentially adopting the predefined criteria (Fig. 1). Fifty-eight articles were selected out of a total of 3139 articles. This subset included 28 articles on maize, 19 on rice, and 11 on soybeans. This subset included 28 articles on maize, 19 on rice, and 11 on soybeans. The transgenic traits represented in these studies primarily encompassed insect resistance (conferred by Cry proteins such as *cry1Ab*, *cry1F*, *cry3Bb1*), herbicide tolerance (mainly involving *cp4 epsps*, *pat*, and *gat* genes), and nutritional enhancement (e.g., glycinin, hLF, psy/crt series), along with other genetic elements. Most of the included studies are from Peking University, Chinese Center for Disease Control and Prevention, China Agricultural University and Monsanto Company.

The comprehensive physiological indicator dataset was developed by collecting raw experimental data from published studies, unifying the units of measurement for all variables, and categorizing the substrate indicators according to the seven established metrics (Table 1). The justification to this comprehensive physiological indicator data set was further confirmed by muti-sourced data correlation analysis.

**Table 1.** Exogenous genes and traits based on the crops of screened literature.

Crops	Exogenous gene	Traits	Classification			Reference
			Nutritionally changed	non-Nutritionally changed		
Maize	*cp4 epsps*	Glyphosate-tolerant		⚪		^11^
Maize	*cry3Bb1*	Insect resistant		⚪		^27^
Maize	*cry1Ab*	Lepidopteran insect-resistant		⚪		^28^
Maize	*cry1F*, *pat*	Glufosinate-tolerant & Insect resistant		⚪		^29^
Maize	*cry 34Ab1*, *cry35Ab1*, *pat*	Glufosinate-tolerant & Insect resistant		⚪		^24^
Maize	*cry3Bb1*, *cp4 epsps*	Glyphosate-tolerant & Insect resistant		⚪		^30^
Maize	*cry34Ab1*, *cry35Ab1*, *pat*	Glufosinate-tolerant & Insect resistant		⚪		^31^
Maize	*cry1F*, *pat*, *cry34Ab1*, *cry35Ab1*	Glufosinate-tolerant & Insect resistant		⚪		^32^
Maize	*gat4621*, *zm-HRA*	Glyphosate-tolerant & ALS-inhibitor-tolerant		⚪		^33^
Maize	*psy1*, *crtI*, *dhar*, *folE*	Provitamin A-enriched & vitamin C-enhanced & folate-boosted	⚪			^34^
Maize	*cry1Ac-M*	Lepidopteran insect-resistant		⚪		^17^
Maize	*cry1F*, *cry34Ab1*, *cry35Ab1*, *pat*	Lepidopteran and coleopteran insect-resistant & Glufosinate-tolerant		⚪		^35^
Maize	*G2-aroA*	Glyphosate-tolerant herbicide		⚪		^36^
Maize	*cry1Ah*	Lepidopteran insect-resistant		⚪		^18^
Maize	*cry1Ac*	Lepidopteran insect-resistant		⚪		^37^
Maize	*cry117Ah*, *G1-aroA*	Lepidopteran insect-resistant & Glyphosate-tolerant		⚪		^38^
Maize	*γ-TMT*	Vitamin E-enhanced	⚪			^23^
Maize	*gat4601*, *gm-hra*	Glyphosate-tolerant & ALS-inhibitor-tolerant		⚪		^39^
Maize	*ZMM28*, *pat*	Yield-enhanced & Glufosinate-tolerant		⚪		^40^
Maize	*cp4 epsps*	Glyphosate-tolerant		⚪		^41^
Maize	*phyA2*	Phosphorus bioavailability-enhanced	⚪			^42^
Maize	*cp4 epsps*, *cry1F*	Glyphosate-tolerant & Lepidopteran insect-resistant		⚪		^43^
Maize	*cry1A.105*, *cp4 epsps*	Glyphosate-tolerant & Lepidopteran insect-resistant		⚪		^44^
Maize	*phyA2*	Phosphorus bioavailability-enhanced	⚪			^45^
Maize	*mCry1Ab*	Lepidopteran insect-resistant		⚪		^46^
Maize	*cry1Ab-ma*	Lepidopteran insect-resistant		⚪		^47^
Maize	*cry1Ab*, *cry1F*, *cp4epsps*	Lepidopteran insect-resistant & Glyphosate-tolerant		⚪		^48^
Maize	*cp4 epsps*	Fatty acid biosynthesis-enhanced	⚪			^14^
Rice	*glycinin*	High-lysine	⚪			^49^
Rice	*cry1Ab*	Lepidopteran insect-resistant			⚪	^50^
Rice	*cry1Ab*	Lepidopteran insect-resistant			⚪	^51^
Rice	*gna*	GNA lectin-expressing	⚪			^52^
Rice	*igf1*	Recombinant human insulin-like growth factor-1 (rhIGF-1) producing	⚪			^53^
Rice	*GL fusion protein gene*	Lysine-enriched storage protein-expressing	⚪			^54^
Rice	*cryAb*, *cryAc*	Lepidopteran insect-resistant			⚪	^55^
Rice	*cry1Ac*, *sck*	Lepidopteran insect-resistant			⚪	^12^
Rice	*SBE*	High-amylose starch	⚪			^56^
Rice	*cry1Ab*	Lepidopteran insect-resistant			⚪	^57^
Rice	*cry1Ab*, *cry1Ac*	Lepidopteran insect-resistant			⚪	^19^
Rice	*cry2A*	Lepidopteran insect-resistant			⚪	^58^
Rice	*cry1Ac*, *sck*	Lepidopteran insect-resistant			⚪	^59^
Rice	*crt E*, *crt B*, *crt Y*, *crt I*	β-carotene production	⚪			^60^
Rice	*AK* & *DHPS* (Up-regulation), *LKR* & *SDH* (Down-regulation)	Lysine-biosynthesis-enhanced	⚪			^13^
Rice	*hLF*	Recombinant human lactoferrin-producing	⚪			^61^
Rice	cry1C*	Lepidopteran insect-resistant			⚪	^21^
Rice	*Bar*, *PhyLf*	Glufosinate-tolerant and phosphorus bioavailability-enhanced	⚪			^62^
Rice	*Psy*, *CrtI*	β-carotene production	⚪			^22^
Soybean	*gat4601*, *gm-hra*	Glyphosate-tolerant & ALS-inhibitor-tolerant			⚪	^63^
Soybean	*gm-fad2-1*, *gm-hra*	High-oleic acid & ALS-inhibitor-tolerant	⚪		⚪	^16^
Soybean	*gm-fad2-1*, *gm-hra*, *cp4 epsps*	High-oleic & ALS-inhibitor-tolerant & Glyphosate-tolerant.	⚪		⚪	^64^
Soybean	*dmo*	Dicamba-tolerant			⚪	^20^
Soybean	*aad-12*, *2mepsps*, *pat*	2,4-D-tolerant & Glyphosate-tolerant & Glufosinate-tolerant			⚪	^65^
Soybean	*aad-12*, *2mepsps*, *pat*	2,4-D-tolerant & Glyphosate-tolerant & Glufosinate-tolerant			⚪	^66^
Soybean	*aad-12*, *2mepsps*, *pat*	2,4-D-tolerant & Glyphosate-tolerant & Glufosinate-tolerant			⚪	^24^
Soybean	*cry1F*, *cry1Ac*, *pat*	Lepidopteran insect-resistant & Glufosinate-tolerant			⚪	^67^
Soybean	*hppd*, *2mepsps*	HPPD-inhibitor-tolerant & Glyphosate-tolerant			⚪	^68^
Soybean	*gat4601*, *gm-hra*	Glyphosate-tolerant & ALS-inhibitor-tolerant			⚪	^69^
Soybean	*aad-12*, *pat*	2,4-D-tolerant & Glufosinate-tolerant			⚪	^70^

### 2.2. Data Analysis and Statistical Methods

The classified analysis was conducted on panel data sourced from the literature, encompassing the type and sex (male and female) of model animal, the consumption dosage and category of GM crops, and the metrics of physiological indicators in the experimental group (GM group) and the control group (non-GM group). Especially, the consumption dosage was indexed by the mass proportion of GM crops in the experimental diet: low dosage was defined as less than 20%, medium dosage ranged from 20% to 50%, and high dosage was defined as 50% or more; the category was defined as non-nutritional GM crops and nutritionally changed GM crops based on introduced gene; One category is non-nutritional plants that are genetically modified to have anti-stress traits such as insect resistance, the other is nutritionally changed plants that are genetically modified to be rich in certain amino acids, vitamins and other nutrients.

The multi-sourced panel data was categorized by crop type and physiological indicator, and a random-effects model was employed using the DerSimonian-Laird response index. The effect size was estimated by Cohen’s standardized mean difference (SMD), adjusted by the Hedges method. The results included 95% confidence intervals (95% CI) based on two-tailed tests. The inter-study variability was denoted by an *I*^2^ statistic exceeding 50%. Statistical significance is defined as a *p*-value of 0.05. In cases where heterogeneity was statistically significant, the sub-panel data for each indicator was further categorized by sex and consumption dosage, and a similar analysis was conducted. Particularly for weight gain, a Monte-Carlo process and a two-tailed t-test were utilized due to constraints in the available data (Table S1-3). All statistical analyses were performed using Stata version 17.0 (Stata Corp, College Station, TX, USA).

## 3. Results

### 3.1. Characteristics of Transgenic Traits, Physiological Indicators, and Animal Models in the Included Studies

The GM crops assessed in these studies were further classified into two categories based on the intended effect of the genetic modification: non-nutritional GM crops and nutritionally changed GM crops (ISAAA, 2025)^26^. Non-nutritional GM crops are those where the introduced gene(s) are intended for purposes other than altering nutritional content, such as conferring resistance to insects (e.g., *Bt* genes) or herbicides (e.g., *epsps* gene), improving agronomic traits, or facilitating selection. Nutritionally changed GM crops encompass those where the primary intent is to deliberately alter the nutritional profile or composition, including modifications for biofortification (e.g., increasing vitamin A, β-carotene), altering fatty acid profiles, or modifying amino acid content. This classification is further detailed in Table 1.

The included GM studies all contained at least one of our selected 25 physiological indicators which can be divided into seven metrics. Although 7 metrics were generally assessed for the health status of mammals, the use of indicators under each metric was not unified. The indicators of RBC, WBC, and PLT concentrations in blood cell concentrations and the indicators of serum ALT and AST concentrations in liver function were widely applied. However, indicators such as serum LDH concentration for cardiovascular function and serum TBIL concentration for liver function were seldom measured (Table 2).

**Table 2.** Physiological indicator system composition based on screened literature.

Studies	Animal	Exposition time /Week	No./Group	Physiological indicators																										Plant
				Weight gain	Relative organ weight						Blood cells concentration						Cardiovascular function		Liver function			Renal function			Electrolyte concentration					
					Brain	Heart	Lung	Kidney	Liver	Spleen	RBC	PLT	WBC	LYM	Neu	MON	CHOL	LDH	ALT	AST	TBIL	BUN	CRE	GLU	K	Na	Ca	P	Cl	
Hammond, Dudek, Lemen, & Nemeth^11^	Rats	13	10		⚪	⚪		⚪	⚪	⚪	⚪	⚪	⚪	⚪	⚪				⚪	⚪		⚪	⚪	⚪	⚪	⚪	⚪	⚪	⚪	Maize
Hammond, Dudek, Lemen, & Nemeth^27^	Rats	13	10		⚪	⚪		⚪	⚪	⚪	⚪	⚪	⚪	⚪	⚪				⚪	⚪		⚪		⚪	⚪	⚪	⚪	⚪	⚪	Maize
Hammond et al.^28^	Rats	13	10		⚪	⚪		⚪	⚪	⚪	⚪	⚪	⚪	⚪	⚪		⚪		⚪	⚪	⚪	⚪	⚪	⚪	⚪	⚪	⚪	⚪	⚪	Maize
MacKenzie et al.^29^	Rats	13	12		⚪	⚪		⚪	⚪	⚪	⚪	⚪	⚪	⚪	⚪	⚪	⚪		⚪	⚪		⚪	⚪	⚪	⚪	⚪	⚪		⚪	Maize
Malley et al.^24^	Rats	13	12		⚪	⚪		⚪	⚪	⚪	⚪	⚪	⚪	⚪	⚪	⚪			⚪	⚪	⚪	⚪	⚪	⚪	⚪	⚪	⚪	⚪	⚪	Maize
Healy, Hammond, & Kirkpatrick^30^	Rats	13	10		⚪	⚪		⚪	⚪	⚪	⚪	⚪	⚪	⚪	⚪		⚪		⚪	⚪	⚪	⚪	⚪	⚪	⚪	⚪	⚪	⚪	⚪	Maize
He et al.^31^	Rats	13	10		⚪	⚪	⚪	⚪	⚪	⚪	⚪	⚪	⚪						⚪	⚪		⚪	⚪	⚪			⚪	⚪		Maize
Appenzeller, Malley, Mackenzie, Hoban, & Delaney^32^	Rats	13	12		⚪	⚪		⚪	⚪	⚪	⚪	⚪	⚪	⚪	⚪	⚪	⚪		⚪	⚪	⚪	⚪	⚪	⚪	⚪	⚪	⚪	⚪	⚪	Maize
Appenzeller et al.^33^	Rats	13	12		⚪	⚪		⚪	⚪	⚪	⚪	⚪	⚪	⚪	⚪	⚪			⚪	⚪	⚪	⚪	⚪	⚪	⚪	⚪	⚪	⚪	⚪	Maize
Arjó et al.^34^	Rats	13	5								⚪	⚪		⚪	⚪	⚪			⚪											Maize
Liu et al.^17^	Rats	13	10		⚪	⚪	⚪	⚪	⚪	⚪	⚪	⚪	⚪					⚪	⚪	⚪				⚪				⚪		Maize
Delaney et al.^35^	Rats	13	12		⚪	⚪		⚪	⚪	⚪	⚪	⚪	⚪	⚪	⚪	⚪	⚪		⚪	⚪	⚪	⚪	⚪	⚪	⚪	⚪	⚪	⚪	⚪	Maize
Zhu et al.^36^	Rats	13	10		⚪	⚪	⚪	⚪	⚪	⚪	⚪	⚪	⚪				⚪	⚪	⚪	⚪		⚪	⚪	⚪				⚪		Maize
Song et al.^18^	Mice	13	10						⚪	⚪	⚪	⚪	⚪	⚪	⚪	⚪	⚪		⚪	⚪		⚪			⚪	⚪	⚪			Maize
Guo et al.^37^	Rats	13	10		⚪	⚪		⚪	⚪	⚪	⚪	⚪	⚪			⚪		⚪	⚪			⚪		⚪						Maize
Han, Zou, He, Huang, & Mei^38^	Rats	13	10		⚪	⚪	⚪	⚪	⚪	⚪	⚪	⚪	⚪				⚪	⚪	⚪	⚪		⚪	⚪	⚪			⚪	⚪	⚪	Maize
Fang et al.^23^	Rats	13	10		⚪	⚪		⚪	⚪	⚪	⚪	⚪	⚪				⚪		⚪	⚪		⚪	⚪	⚪						Maize
Zou, Lang, Liu, Huang, & He^39^	Rats	13	10		⚪	⚪	⚪	⚪	⚪	⚪	⚪	⚪	⚪				⚪	⚪	⚪	⚪		⚪		⚪			⚪	⚪	⚪	Maize
Carlson et al.^40^	Rats	13	16	⚪																										Maize
Steinberg et al.^41^	Rats	13	16		⚪	⚪		⚪	⚪	⚪	⚪	⚪	⚪						⚪	⚪					⚪	⚪	⚪	⚪	⚪	Maize
Liang et al.^42^	Rats	13	10		⚪	⚪		⚪	⚪	⚪	⚪	⚪	⚪				⚪	⚪	⚪	⚪		⚪	⚪	⚪			⚪			Maize
Smith et al.^43^	Rats	13	16	⚪																										Maize
Zhang et al.^44^	Rats	7	8			⚪		⚪	⚪	⚪	⚪	⚪	⚪						⚪	⚪		⚪		⚪						Maize
Sun et al.^45^	Rats	13	10		⚪	⚪		⚪	⚪	⚪	⚪	⚪	⚪				⚪	⚪	⚪	⚪		⚪	⚪	⚪			⚪			Maize
Zhang et al.^46^	Rats	10	20	⚪	⚪			⚪	⚪	⚪	⚪	⚪	⚪						⚪	⚪	⚪	⚪								Maize
Wang et al.^47^	Rats	52	#	⚪	⚪				⚪		⚪	⚪	⚪						⚪	⚪	⚪	⚪			⚪	⚪	⚪		⚪	Maize
Zhang et al.^48^	Rats	13	10		⚪	⚪			⚪	⚪	⚪	⚪	⚪						⚪	⚪		⚪	⚪	⚪	⚪	⚪			⚪	Maize
Chen et al.^14^	Rats	13	10	⚪		⚪		⚪	⚪		⚪	⚪	⚪				⚪		⚪	⚪		⚪	⚪	⚪	⚪	⚪			⚪	Maize
Momma et al.^49^	Rats	4	7	⚪							⚪	⚪	⚪		⚪	⚪			⚪	⚪										Rice
Wang et al.^50^	Rats	13	10	⚪				⚪	⚪	⚪							⚪		⚪			⚪	⚪	⚪						Rice
Schrøder et al.^51^	Rats	13	16		⚪	⚪		⚪	⚪	⚪	⚪	⚪	⚪		⚪	⚪	⚪					⚪	⚪	⚪	⚪	⚪				Rice
Poulsen et al.^52^	Rats	13	16		⚪	⚪		⚪	⚪	⚪	⚪	⚪			⚪	⚪	⚪						⚪	⚪						Rice
Tang et al.^53^	Rats	13	16		⚪	⚪		⚪	⚪	⚪	⚪	⚪	⚪		⚪	⚪	⚪		⚪	⚪			⚪	⚪	⚪	⚪				Rice
Zhou et al.^54^	Rats	13	10		⚪	⚪	⚪	⚪	⚪	⚪	⚪	⚪	⚪				⚪	⚪	⚪			⚪	⚪	⚪	⚪	⚪	⚪		⚪	Rice
Wang, Yu, Hu, Jia, & Xu^55^	Rats	10	12		⚪	⚪		⚪	⚪	⚪	⚪	⚪	⚪				⚪		⚪	⚪		⚪	⚪	⚪			⚪			Rice
Zhang, Zhuo, Tian, Piao, & Yang^12^	Rats	78	20		⚪	⚪		⚪	⚪	⚪	⚪	⚪	⚪				⚪	⚪	⚪	⚪		⚪		⚪						Rice
Zhou et al.^56^	Rats	13	10		⚪	⚪	⚪	⚪	⚪	⚪	⚪	⚪	⚪				⚪	⚪	⚪	⚪		⚪	⚪	⚪	⚪	⚪	⚪		⚪	Rice
Song et al.^57^	Rats	13	10		⚪	⚪	⚪	⚪	⚪	⚪	⚪	⚪	⚪						⚪	⚪		⚪	⚪	⚪			⚪	⚪		Rice
Mao et al.^19^	Monkey	52	5		⚪	⚪	⚪	⚪	⚪	⚪	⚪	⚪	⚪		⚪	⚪	⚪		⚪	⚪		⚪	⚪	⚪	⚪	⚪			⚪	Rice
Zou, Huang, Xu, Luo, & He^58^	Rats	13	10		⚪	⚪	⚪	⚪	⚪	⚪	⚪	⚪	⚪				⚪	⚪	⚪	⚪				⚪						Rice
Hu, Zhuo, Gong, Piao, & Yang^59^	Rats	13	20								⚪	⚪	⚪				⚪	⚪	⚪	⚪		⚪		⚪						Rice
Wu et al.^60^	Rats	13	9			⚪	⚪	⚪	⚪	⚪	⚪	⚪	⚪				⚪		⚪	⚪		⚪	⚪	⚪				⚪		Rice
Yang et al.^13^	Rats	13	10	⚪	⚪	⚪	⚪	⚪	⚪	⚪	⚪	⚪	⚪				⚪	⚪	⚪	⚪		⚪	⚪	⚪			⚪	⚪		Rice
Zhang et al.^61^	Rats	13	10	⚪	⚪		⚪	⚪	⚪	⚪	⚪	⚪	⚪				⚪	⚪	⚪	⚪		⚪	⚪	⚪			⚪	⚪		Rice
Tang et al.^21^	Rats	52	#		⚪	⚪		⚪	⚪	⚪	⚪	⚪	⚪		⚪				⚪	⚪		⚪	⚪	⚪	⚪	⚪	⚪		⚪	Rice
Li et al.^62^	Rats	13	10	⚪		⚪		⚪	⚪	⚪	⚪	⚪	⚪		⚪	⚪	⚪	⚪	⚪	⚪		⚪	⚪							Rice
Xia et al.^22^	Rats	8	10		⚪	⚪		⚪	⚪	⚪	⚪	⚪	⚪		⚪	⚪	⚪		⚪	⚪		⚪	⚪	⚪	⚪	⚪	⚪		⚪	Rice
Appenzeller et al.^63^	Rats	13	12		⚪	⚪		⚪	⚪	⚪	⚪	⚪	⚪				⚪		⚪	⚪		⚪	⚪	⚪						Soybean
Delaney et al.^16^	Rats	13	12		⚪	⚪		⚪	⚪	⚪	⚪	⚪	⚪				⚪		⚪	⚪		⚪	⚪	⚪						Soybean
Qi et al.^64^	Rats	13	10		⚪	⚪	⚪	⚪	⚪	⚪	⚪	⚪	⚪				⚪		⚪	⚪		⚪	⚪	⚪						Soybean
Wang et al.^20^	Rats	13	10		⚪	⚪	⚪	⚪	⚪	⚪	⚪	⚪	⚪				⚪		⚪	⚪		⚪	⚪	⚪						Soybean
Papineni et al.^65^	Rats	13	12	⚪										⚪																Soybean
Papineni, Passage, Ekmay, & Thomas^66^	Rats	13	16	⚪	⚪	⚪		⚪	⚪	⚪	⚪	⚪	⚪	⚪	⚪		⚪		⚪	⚪		⚪	⚪							Soybean
Qian et al.^24^	Rats	13	10	⚪		⚪		⚪	⚪	⚪	⚪	⚪	⚪	⚪	⚪		⚪		⚪	⚪		⚪	⚪	⚪						Soybean
Qian et al.^67^	Rats	13	#		⚪	⚪		⚪	⚪	⚪	⚪	⚪	⚪	⚪	⚪		⚪		⚪	⚪		⚪	⚪	⚪						Soybean
Xie et al.^68^	Rats	13	10		⚪	⚪	⚪	⚪	⚪	⚪	⚪	⚪	⚪						⚪	⚪		⚪	⚪	⚪						Soybean
Zou et al.^69^	Rats	13	10		⚪	⚪	⚪	⚪	⚪	⚪	⚪	⚪	⚪				⚪		⚪	⚪		⚪		⚪						Soybean
Zhang et al.^70^	Rats	13	10	⚪		⚪		⚪	⚪	⚪	⚪	⚪	⚪	⚪	⚪		⚪		⚪	⚪		⚪	⚪	⚪						Soybean

#The number of animals in each experimental group in the study is not the same.

PLT: platelet, WBC: white blood cells, LYM: lymphocyte, NEU: neutrophilic granulocytes, MON: monocytes, LDH: lactate dehydrogenase, TBIL: total bilirubin, BUN: blood urea nitrogen, Glu: glucose, Ca: calcium, P: phosphorus, Cl: chloride.

Besides, rats, specifically the Sprague-Dawley and Wistar strains, were the most common model animals in the included studies between GM and non-GM groups, and other employed monkeys and mice. The variance from model animal species was low, and by grouping them, the model animals could be characterized by rats and crab-consuming macaques.

### 3.2. Variations Were Not Found on Mammalian Body Weight While Found on Relative Organ Weights After GM Crop Consumption

#### 3.2.1. Variations Were Not Found on Mammalian Body Weight While Found on Relative Organ Weights After GM Maize Consumption

No notable disparity in body weight gain was observed in the GM-maize groups compared to non-GM controls after 7–91 days of consumption (Tables S1). However, statistically significant differences were identified in the relative weights of certain organs.

The relative brain, lung, kidney, and spleen weights in the GM-maize group were lower than its non-GM control group (Brain: SMD: −0.07, 95% CI: −0.16, 0.03, *p*-value > .05; Figure S1; Lung: SMD: −0.09, 95% CI: −0.29, 0.11, *p*-value > .05, Figure S2; Kidney: SMD: −0.02, 95% CI: −0.13, 0.08, *p*-value > .05, Figure S3; Spleen: SMD: −0.08, 95% CI: −0.16, 0.01, *p*-value > .05; Figure S4). While the relative heart weight was higher (SMD: 0.07, 95% CI: −0.03, 0.17, *p*-value > .05; Figure S5).

**Figure 2.**
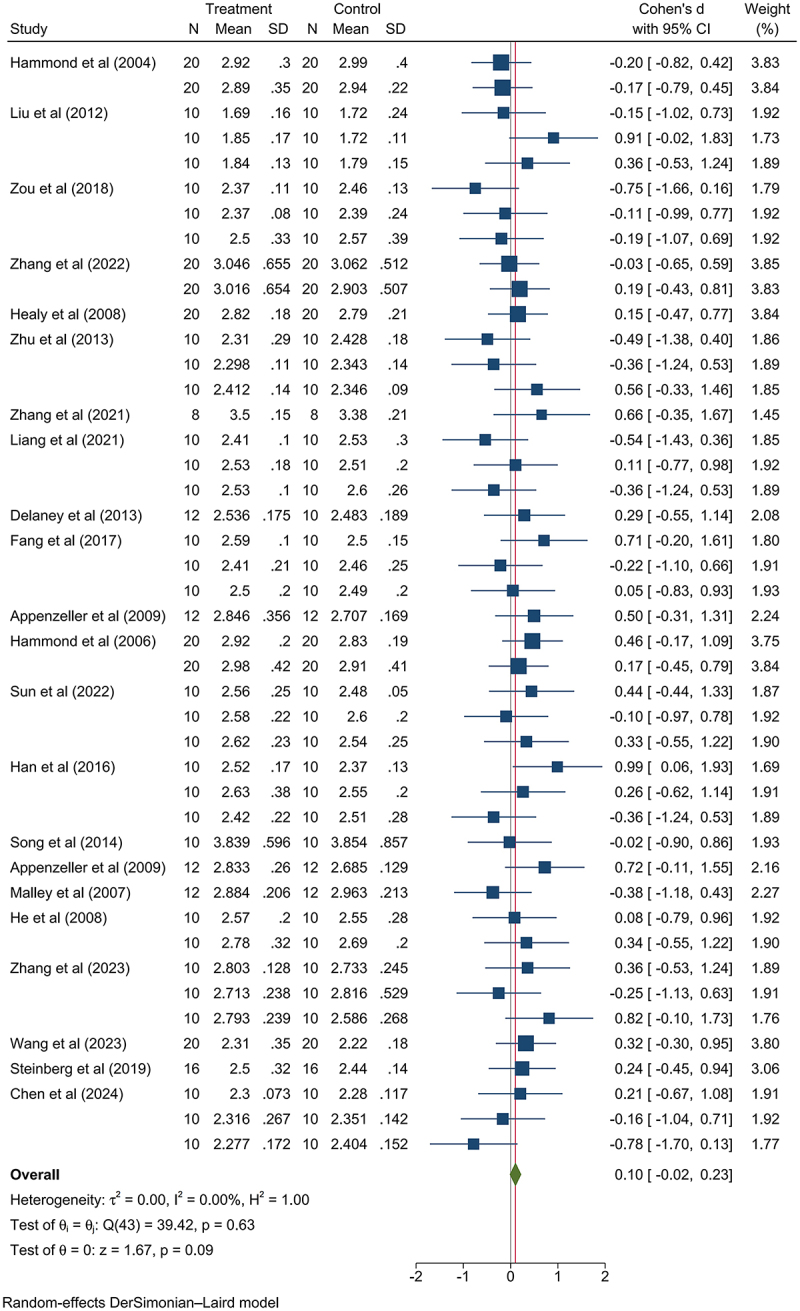
Consuming GM maize showed no statistically significant increase on female mammalian relative liver weight after removing the literature with significant contribution.^28,29^ N: number of model animals; SD: standard deviation; Cohen’s *d*: Cohen’s difference; 95% ci: 95% confidence interval.

The relative weights of the liver were higher, being statistically significant (SMD: 0.11, 95% CI: 0.03, 0.19, *p*-value < .05; Figure S6). This pronounced increase in liver weight was not attributed to differing levels of GM maize consumption dosage or category, but was peculiar to the female group (female group: SMD: 0.14, 95% CI: 0.03, 0.26, *p*-value < .05; Figure S7-11). Among the analyzed data of relative liver weight in the female group, non-nutritional GM maize consumption data from Hammond et al. (2006)^28^ and MacKenzie et al. (2007)^29^ contributed to the difference (Fig. 2). The GM maize in Hammond et al. (2006)^28^ encoded a *Cry3Bb1* protein, while that in MacKenzie et al. (2007)^29^ encoded *Cry1F* and *PAT* proteins; the parental maize line used in Hammond et al. (2006)^28^ as the negative control was formulated by Purina TestDiet (Richmond, IN), while in MacKenzie et al (2007),^29^ the negative control parental maize lines were 33P66 and 33J56. The differences in the negative control maize lines used in the two studies may have contributed to the significant findings observed in their respective analyses. Since there was no pathological change of liver observed in the two articles mentioned above, the difference was not considered as health risk (Table 3).

**Table 3.** Comprehensive effects of GM-crops consumption on mammal relative organ weight.

Indicators	No. of Articles	Cohen’s *d* (95% CI)	*p*-value	Plant
Brain	22	−0.07 [−0.16, 0.03]	.16	Maize
Heart	22	0.07 [−0.03, 0.17]	.15	Maize
Lung	5	−0.09 [−0.29, 0.11]	.39	Maize
Kidney	22	−0.02 [−0.13,0.08]	.69	Maize
Liver	25	0.11 [0.03, 0.19]	.01*	Maize
Liver ^1^	23	0.10 [−0.02, 0.23]	.09	Maize
Spleen	23	−0.08 [−0.16, 0.01]	.09	Maize
Brain	14	0.02 [−0.14, 0.17]	.83	Rice
Heart	15	0.04 [−0.11, 0.18]	.63	Rice
Lung	8	−0.21 [−0.38, −0.04]	.02*	Rice
Kidney	17	0.17 [0.05, 0.30]	.01*	Rice
Kidney^1^	4	0.60 [0.21,0.99]	.00**	Rice
Kidney^2^	4	−0.04 [−0.39,0.32]	.84	Rice
Kidney^3^	6	0.34 [0.09,0.58]	.01*	Rice
Kidney^4^	9	0.12 [−0.06,0.30]	.20	Rice
Liver	17	−0.09 [−0.21, 0.04]	.17	Rice
Spleen	17	−0.01 [−0.16, 0.14]	.92	Rice
Brain	8	0.04 [−0.09, 0.18]	.54	Soybean
Heart	10	−0.02 [−0.14, 0.10]	.77	Soybean
Lung	4	−0.04 [−0.22, 0.14]	.66	Soybean
Kidney	10	0.07 [−0.05, 0.19]	.26	Soybean
Liver	10	0.09 [−0.03, 0.22]	.14	Soybean
Spleen	10	0.07 [−0.04, 0.19]	.21	Soybean

*: *p*-value < .05; Liver ^1^: the analysis result of female relative liver weight after removing the data of non-nutritional GM maize consumption from Hammond et al.^28^ and MacKenzie et al.^29^; Kidney ^1^: the analysis result of relative kidney weight after medium dosage nutritionally changed GM rice consumption; Kidney ^2^: the analysis result of relative kidney weight concentration after medium dosage non-nutritional GM rice consumption; Kidney ^3^: the analysis result of relative kidney weight concentration after high dosage nutritionally changed GM rice consumption; Kidney ^4^: the analysis result of relative kidney weight concentration after high dosage non-nutritional GM rice consumption; No. of Articles: Number of Articles.

#### 3.2.2. Variations Were Not Found on Mammalian Body Weight While Found on Relative Organ Weights After GM Rice Consumption

No notable disparity in body weight gain was observed in the GM-rice groups compared to non-GM controls (Tables S2).

The relative brain and heart weights in the GM-rice group were higher than its non-GM control group (Brain: SMD: 0.02, 95% CI: −0.14, 0.17, *p*-value > .05; Figure S12; heart: SMD: 0.04, 95% CI: −0.11, 0.18, *p*-value > .05; Figure S13). A decrease in the relative weights of liver and spleen were observed (Liver: SMD: −0.09, 95% CI: −0.21, 0.04, *p*-value > .05; Figure S14; spleen: SMD: −0.01, 95% CI: −0.16, 0.14, *p*-value > .05; Figure S15).

**Figure 3.**
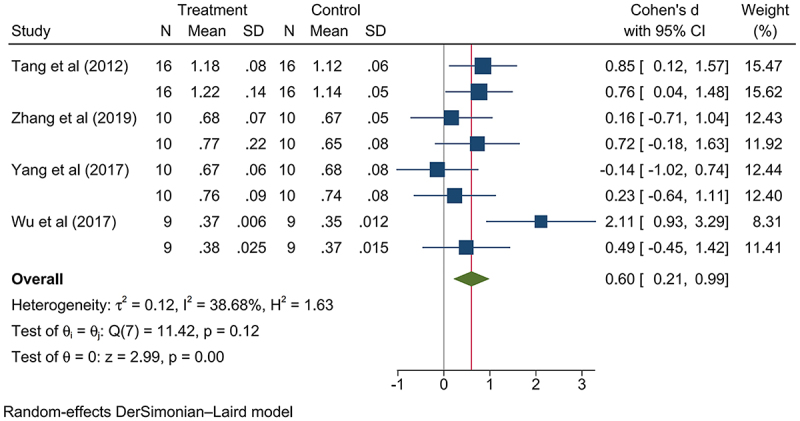
Consuming medium dosage of nutritionally changed GM rice presented a statistically significant increase on mammalian relative kidney weight.

**Figure 4.**
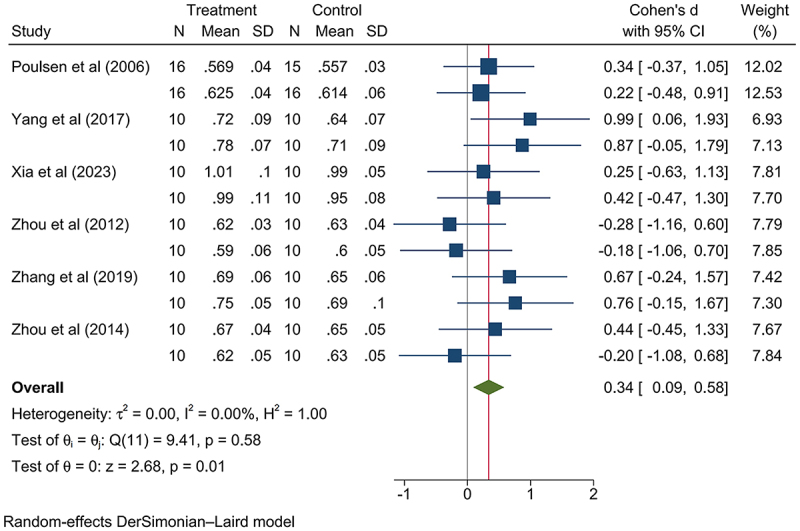
Consuming high dosage of nutritionally changed GM rice presented a statistically significant increase on mammalian relative kidney weight.

The GM-rice group exhibited higher relative kidney weight increase (SMD: 0.17, 95% CI: 0.05, 0.30, *p*-value < .05; Figure S16), and this was attributable to taking medium and high dosage nutritionally changed GM rice (low dosage: SMD: −0.23, 95% CI: −0.55, 0.08, *p*-value > .05; Figures S17; medium dosage: SMD: 0.31, 95% CI: 0.01, 0.62, *p*-value < .05, Figure S18; high dosage: SMD: 0.19, 95% CI: 0.05, 0.33, *p*-value < .05, Figure S19; Figure S20-21; Figures 3,4). The nutritionally changed GM rice expressed various beneficial components, including High-Free-Lysine (HFL),^13^ human insulin-like growth factor-1 (rhIGF-1),^53^ high-lysine proteins,^54^ high-amylose components,^56^ β-carotene,^22,60^ and human lactoferrin (hLF).^61^ Meanwhile, since this increase has not been associated with abnormal renal lesions, GM rice consumption is not considered a health risk. Differing, the relative weight of the lung decreased (SMD: −0.21, 95% CI: −0.38, −0.04, *p*-value < .05; Figure S22); for the significant difference on this indicator was observed in those consuming a low dosage of GM rice but not found in those consuming a medium and high dosage of GM rice (Low dosage: SMD: −0.54, 95% CI: −1.02, −0.07, *p*-value < .05; Medium dosage: SMD: −0.22, 95% CI: −0.49, 0.05, *p*-value > .05; High dosage: SMD: −0.08, 95% CI: −0.32, 0.16, *p*-value > .05; Figures S23-25); thus, the relative lung weight might not respond to GM rice consumption (Table 3).

#### 3.2.3. No Variations Were Found on Mammalian Body Weight After GM Soybean Consumption

No notable disparity in body weight gain was observed in the GM-soybean groups compared to non-GM controls (Tables S3). However, some differences were identified in the relative weights of certain organs.

The relative brain, kidney, liver, and spleen weights in the GM-soybean group were higher than its non-GM control group (Brain: SMD: 0.04, 95% CI: −0.09, 0.18, *p*-value > .05; Figure S26; Kidney: SMD: 0.07, 95% CI: −0.05, 0.19, *p*-value > .05, Figure S27; Liver: SMD: 0.09, 95% CI: −0.03, 0.22, *p*-value > .05, Figure S28; spleen: SMD: 0.07, 95% CI: −0.04, 0.19, *p*-value > .05, Figure S29); the relative weights of the heart and lung were lower than the control group (Heart: SMD: −0.02, 95% CI: −0.14, 0.10, *p*-value > .05, Figure S30; Lung: SMD: −0.04, 95% CI: −0.22, 0.14, *p*-value > .05, Figure S31). However, all these responses were not statistically significant (Table 3).

### 3.3. An Increased GLU Concentration was Found in Nutritionally Changed gm-Maize Group, While No Variations Were Found in the Renal Functional Indicators After GM Rice and Soybean Consumption

#### 3.3.1. An Increased GLU Concentration was Found in Nutritionally Changed gm-Maize Group

**Figure 5.**
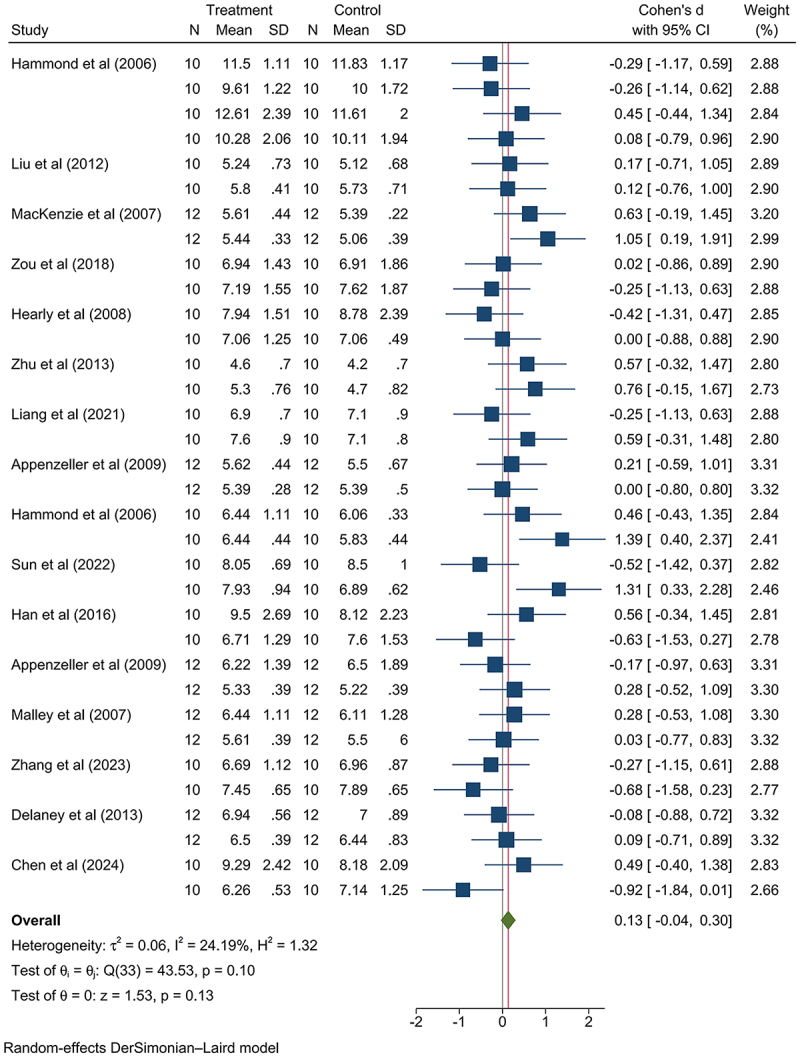
Consuming medium dosage of GM maize showed no statistically significant increase on mammalian serum GLU concentration after removing the literature with significant contribution.

**Figure 6.**
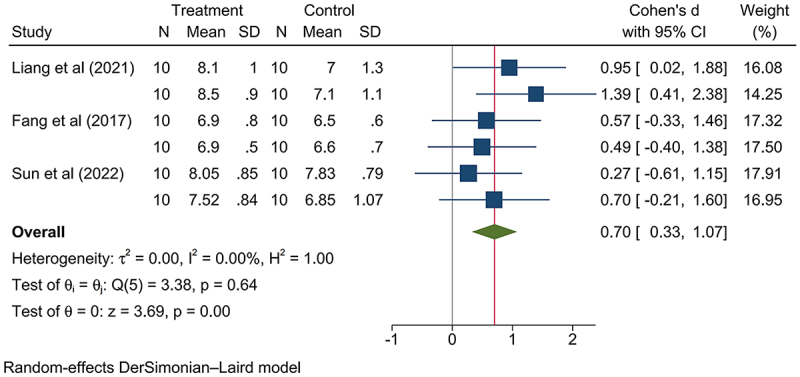
Consuming high dosage of nutritionally changed GM maize presented a statistically significant increase on mammalian serum GLU concentration.

The GM maize consumption resulted in elevated concentrations of both BUN and CRE compared to the non-GM control group (Table 4) (BUN: maize: SMD: 0.01, 95% CI: −0.11, 0.13, *p*-value > .05, Figure S32; CRE: SMD: 0.05, 95% CI: −0.10, 0.20, *p*-value > .05, Figure S33). However, the concentration of GLU was significantly elevated compared to the non-GM group (SMD: 0.19, 95% CI: 0.06, 0.31, *p*-value < .01; Table S8, Figure S34). Among the analyzed data on medium dosage consumption of GM maize, the data from Fang et al. (2017)^23^ on nutritionally changed GM maize contributed to the observed elevation in GLU concentration (Fig. 5). The parental maize line used as the negative control in the study was Zheng58. Notably, Zheng58 has a relatively higher lysine, which might have contributed to the significance of the findings reported by Fang et al. (2017).^23^ For the high dosage consumption of GM maize, the increase in the GLU levels was attributed to the consumption of the nutritionally changed GM maize (low dosage: SMD: −0.01, 95% CI: −0.18, 0.17, *p*-value > .05, Figure S35; medium dosage: SMD: 0.21, 95% CI: 0.01, −0.41, *p*-value < .05, Figure S36; high dosage: SMD: 0.36, 95% CI: 0.10, 0.63, *p*-value < .05, Figure S37; Figure S38; Fig. 6). The nutritionally changed GM maize has been designed to express multiple beneficial components, such as α-tocopherol^23^ and phytase,^42,45^ enhancing its nutritional profile. These components may contribute to the observed metabolic differences; α-tocopherol can improve insulin sensitivity via its antioxidant properties, while phytase enhances nutrient availability by degrading phytic acid.^71,72^ Notably, the reported GLU ranges (GM: 4.3–12.61 mmol/L; non-GM: 4.2–11.83 mmol/L) (Experimental Center for Life Science, 2021)^73^ show substantial inherent fluctuation. Despite slightly higher bounds in the GM group, this minor increase is not considered to pose a significant health risk.^74^

**Table 4.** Comprehensive effects of GM-crops consumption on mammal renal functional indicator.

Indicators	Included articles number	Cohen’s *d* (95% CI)	*p*-value	Plant
BUN	23	0.01 [−0.11, 0.13]	.83	Maize
CRE	16	0.05 [−0.10, 0.20]	.52	Maize
GLU	21	0.19 [0.06,0.31]	.00**	Maize
GLU^1^	16	0.13 [−0.04,0.30]	.13	Maize
GLU^2^	3	0.70 [0.31,1.09]	.00**	Maize
GLU^3^	8	0.14 [−0.19,0.46]	.41	Maize
BUN	15	0.12 [−0.07, 0.31]	.23	Rice
CRE	15	−0.20 [−0.41, 0.00]	.06	Rice
GLU	17	0.09 [−0.10, 0.27]	.37	Rice
BUN	10	0.14 [−0.02, 0.30]	.10	Soybean
CRE	9	0.01 [−0.14, 0.15]	.89	Soybean
GLU	9	−0.09 [−0.30, 0.13]	.44	Soybean

**p-value < .01; BUN: blood urea nitrogen; CRE: creatinine; GLU: glucose. GLU^1^: the analysis result of GLU concentration after removing the data of nutritionally changed GM maize consumption from Fang et al. (2017)^23^; GLU^2^: the analysis result of GLU concentration after high dosage nutritionally changed GM maize consumption. GLU^3^: he analysis result of GLU concentration after high dosage non-nutritional GM maize consumption.

#### 3.3.2. No Variations Were Found in the Renal Functional Indicators After GM Rice Consumption

The effects of GM rice consumption on renal function indicators were statistically insignificant (Table 4). The observed trend in GLU and BUN concentration showed higher levels (GLU: SMD: 0.09, 95% CI: −0.10, 0.27, *p*-value > .05; Figure S39; BUN: SMD: 0.12, 95% CI: −0.07, 0.31, *p*-value > .05, Figures S40), whereas the CRE concentration showed lower level (SMD: −0.20, 95% CI: −0.41, 0.00, *p*-value > .05, Figure S41). These results were not statistically confirmed.

#### 3.3.3. No Variations Were Found in the Renal Functional Indicators After GM Soybean Consumption

The effects of GM soybean consumption on renal function indicators were statistically insignificant (Table 4). The observed trend in GLU concentration showed lower levels (SMD: −0.09, 95% CI: −0.30, 0.13, *p*-value > .05; Figure S42), whereas the CRE and BUN concentrations showed higher level (CRE: SMD: 0.01, 95% CI: −0.14, 0.15, *p*-value > .05; Figures S43; BUN: SMD: 0.14, 95% CI: −0.02, 0.30, *p*-value > .05; Figures S44), though this was not statistically confirmed. These results were not statistically confirmed.

### 3.4. No Variations Were Found in the Electrolyte Functional Indicators After GM Crop Consumption

#### 3.4.1. No Variations Were Found in the Electrolyte Functional Indicators After GM Maize Consumption

The statistically significant influences of GM maize consumption on electrolyte function were not observed (Table 5). Serum concentrations of K^+^, Ca2^+^ and P were higher (K^+^: SMD: 0.04, 95% CI: −0.15, 0.22, *p*-value > .05; Figure S45; Ca^2+^: SMD: 0.06, 95% CI: −0.09, 0.20, *p*-value > .05; Figure S46; P: SMD: 0.01, 95% CI: −0.15, 0.17, *p*-value > .05; Figure S47). Serum concentrations of Na^+^ showed no change (SMD: 0.00, 95% CI: −0.09, 0.20, *p*-value > .05; Figure S48). Serum Cl^−^ concentration was lower (SMD: −0.06, 95% CI: −0.28, 0.16, *p*-value > .05; Figure S49). No statistically significant differences in these electrolyte functional indicators were observed.

**Table 5.** Comprehensive effects of feeding with GM crops on mammal electrolyte functional indicator.

Indicators	Included articles number	Cohen’s *d* (95% CI)	*p*-value	Plant^a^
Serum K	14	0.04 [−0.15, 0.22]	.69	Maize
Serum Na	14	0.00 [−0.09, 0.20]	.97	Maize
Serum Ca	17	0.06 [−0.09, 0.20]	.45	Maize
Serum P	14	0.01[−0.15, 0.17]	.89	Maize
Serum CL	15	−0.06 [−0.28, 0.16]	.58	Maize
Serum K	7	0.14 [−0.13, 0.40]	.31	Rice
Serum Na	7	0.15 [−0.21, 0.50]	.42	Rice
Serum Ca	8	−0.22 [−0.47, 0.03]	.08	Rice
Serum P	4	−0.13 [−0.17, 0.43]	.40	Rice
Serum CL	5	−0.43 [−0.85, −0.01]	.04*	Rice

*: *p*-value < .05; K: K^+^ concentration; Na: Na^+^ concentration; Ca^2+^ concentration; CL: Cl^−^ concentration.

#### 3.4.2. No Variations Were Found in the Electrolyte Functional Indicators After GM Rice Consumption

The statistically significant influences of GM rice consumption on electrolyte function were presented on Cl^−^ (Table 5). The GM-rice group was collectively lower in Cl^−^ concentrations (SMD: −0.43, 95% CI: −0.85, −0.01, *p*-value < .05; Figure S50). However, this difference was not attributable to either consumption dosage (Low dosage: SMD: −0.88, 95% CI: −2.95, 1.19, *p*-value > .05; Figure S51; Medium dosage: SMD: −1.2, 95% CI: −2.65, 0.24, *p*-value > .05; Figure S52; High dosage: SMD: −0.13, 95% CI: −0.47, 0.21, *p*-value > .05; Figure S53) or mammalian sex (Male: SMD: −0.56, 95% CI: −1.23, 0.12, *p*-value > .05; Figure S54; Female: SMD: −0.31, 95% CI: −0.86, 0.23, *p*-value > .05; Figure S55).

Other electrolyte parameters showed no significant alterations: serum K^+^ and Na^+^ concentrations were higher (K^+^ : SMD: 0.14, 95% CI: −0.13, 0.40, *p*-value > .05; Figure S56; Na^+^: SMD: 0.15, 95% CI: −0.21, 0.50, *p*-value > .05; Figure S57); serum Ca^2 +^ and P concentrations were lower (Ca^2 +^ : SMD: −0.22, 95% CI: −0.47, 0.03, *p*-value > .05; Figure S58; P: SMD: −0.13, 95% CI: −0.17, 0.43, *p*-value > .05; Figure S59).

### 3.5. No Variations Were Found in the Blood Cell Concentration After GM Crop Consumption

The influences of GM crop consumption on blood cell concentration are summarized in Table 6. In contrast to respective non-GM groups, WBC concentrations were lower in the GM-maize group (SMD: −0.01, 95% CI: −0.11, 0.09, *p*-value > .05; Figure S60) and higher in the GM-rice group (SMD: 0.06, 95% CI: −0.14, 0.26, *p*-value > .05; Figure S61) and the GM-soybean group (SMD: 0.01, 95% CI: −0.14, 0.15, *p*-value > .05; Figure S62); LYM concentrations were lower in the GM-maize (SMD: −0.08, 95% CI: −0.29, 0.12, *p*-value > .05; Figure S63), GM-rice (SMD: −0.19, 95% CI: −0.51, 0.12, *p*-value > .05; Figure S64), and GM-soybean (SMD: −0.1, 95% CI: −0.31, 0.12, *p*-value > .05; Figure S65) groups; Neu concentrations were lower in the GM-maize (SMD: −0.04, 95% CI: −0.21, 0.12, *p*-value > .05; Figure S66), and GM-rice (SMD: −0.18, 95% CI: −0.64, 0.29, *p*-value > .05; Figure S67), but higher in GM-soybean (SMD: 0.14, 95% CI: −0.08, 0.37, *p*-value > .05; Figure S68) groups; MON concentrations were lower in GM-maize group (SMD: −0.13, 95% CI: −0.39, 0.14, *p*-value > .05; Figure S69), while higher in GM-rice (SMD: 0.05, 95% CI: −0.28, 0.38, *p*-value > .05; Figure S70) group; PLT concentrations were higher invariant to crop types (maize: SMD: 0.03, 95% CI: −0.09, 0.15, *p*-value > .05, Figure S71; rice: SMD: 0.14, 95% CI: −0.04, 0.33, *p*-value > .05, Figure S72; soybean: SMD: 0.04, 95% CI: −0.12, 0.20, *p*-value > .05, Figure S73). However, no statistically significant differences in these physiological indicators were observed.

**Table 6.** Comprehensive effects of GM-crops consumption on concentration of the mammal blood cell.

Indicators	No. of Articles	Cohen’s *d* (95% CI)	*p*-value	Plant
RBC	26	0.04 [−0.09, 0.16]	.55	Maize
PLT	25	0.03 [−0.09, 0.15]	.67	Maize
WBC	26	−0.01 [−0.11, 0.09]	.86	Maize
LYM	11	−0.08 [−0.29, 0.12]	.42	Maize
NEU	11	−0.04 [−0.21, 0.12]	.61	Maize
MON	7	−0.13 [−0.39, 0.14]	.35	Maize
RBC	18	0.22 [0.03, 0.41]	.02*	Rice
PLT	18	0.14 [−0.04, 0.33]	.13	Rice
WBC	17	0.06 [−0.14, 0.26]	.55	Rice
LYM	11	−0.19 [−0.51, 0.12]	.23	Rice
NEU	8	−0.18 [−0.64, 0.29]	.45	Rice
MON	7	0.05 [−0.28, 0.38]	.78	Rice
RBC	10	0.12 [−0.00, 0.24]	.05	Soybean
PLT	10	0.04 [−0.12, 0.20]	.61	Soybean
WBC	10	0.01 [−0.14, 0.15]	.94	Soybean
LYM	5	−0.1 [−0.31, 0.12]	.39	Soybean
NEU	4	0.14 [−0.08, 0.37]	.22	Soybean

*: *p*-value < .05; RBC: red blood cell; PLT: platelet; WBC: white blood cell; LYM: lymphocyte; NEU: neutrophilic granulocyte, MON: monocyte.

The concentrations of RBC in GM groups were higher than the non-GM groups (maize: SMD: 0.04, 95% CI: −0.09, 0.16, *p*-value > .05, Figure S74; rice: SMD: 0.22, 95% CI: 0.03, 0.41, *p-*value < 0.01; Figures S75; soybean: SMD: 0.12, 95% CI: −0.00, 0.24, *p*-value = .05, Figure S76). For the statistically significant response in the GM-rice group was linked to the medium dosage level of GM rice consumption, but not to the high dosage (Medium dosage: SMD: 0.54, 95% CI: 0.24, 0.85, *p*-value < .05; Figures S77-79), this statistically significant difference might not be due to GM rice consumption.

### 3.6. No Variations Were Found in the Cardiovascular Functional Indicators After GM Crop Consumption

The relative effects between the GM groups and the non-GM groups on cardiovascular functional indicators showed no significant correlations (Table 7). The integrated effects on CHOL concentration were higher with GM maize (SMD: 0.09, 95% CI: −0.04, 0.22, *p*-value > .05; Figures S80) and GM soybean (SMD: 0.14, 95% CI: −0.04, 0.32, *p*-value > .05; Figures S81), but lower with GM rice (SMD: −0.01, 95% CI: −0.23, 0.21, *p*-value > .05; Figures S82) consumption, showing no statistical significance, despite the data Wu et al. detected exhibiting a notably substantial deviation (SMD: 13.91, Weight: 0.19%; Figures S82).^60^ This divergence did not influence the overall statistical significance. The enzyme activity of LDH in the GM-maize group (SMD: −0.14, 95% CI: −0.35, 0.06, *p*-value > .05; Figures S83) and GM-rice group (SMD: −0.01, 95% CI: −0.26, 0.23, *p*-value > .05; Figures S84) were lower than respective non-GM groups.

**Table 7.** Comprehensive effects of GM-crops consumption on mammal cardiovascular functional indicator.

Indicators	Included articles number	Cohen’s *d* (95% CI)	*p*-value	Plant
CHOL	18	0.09 [−0.04, 0.22]	.19	Maize
LDH	7	−0.14 [−0.35, 0.06]	.17	Maize
CHOL	16	−0.01 [−0.23, 0.21]	.93	Rice
LDH	8	−0.01 [−0.26, 0.23]	.92	Rice
CHOL	9	0.14 [−0.04, 0.32]	.13	Soybean

CHOL: cholesterol; LDH: lactate dehydrogenase.

### 3.7. No Variations Were Found in the Liver Functional Indicators After GM Crop Consumption

The relative effects over liver functional indicators exhibited no statistical significance (Table 8), and the influence on GM groups could not be statistically confirmed. The overall GM-maize group indicated a lower ALT activity (SMD: −0.09, 95% CI: −0.21, 0.04, *p*-value > .05; Figures S85) and a higher AST activity level (SMD: 0.03, 95% CI: −0.10, 0.16, *p*-value > .05; Figures S86). For GM-rice group, the ALT activity level was higher (SMD: 0.03, 95% CI: −0.20, 0.26, *p*-value > .05; Figures S87), and the AST activity level was lower (SMD: −0.11, 95% CI: −0.37, 0.14, *p*-value > .05; Figures S88). For GM-soybean group, the ALT activity level was lower (SMD: −0.03, 95% CI: −0.16, 0.11, *p*-value > .05; Figures S89), and the AST activity level was higher (SMD: 0.11, 95% CI: −0.03, 0.25, *p*-value > .05; Figures S90). Given that elevated simultaneous enzyme activity of ALT and AST would indicate potential liver damage, the likeliness of hepatic dysfunction was relatively low. In the case of GM-maize group, the TBIL level was higher than the non-GM group (SMD: 0.04, 95% CI: −0.13, 0.22, *p*-value > .05; Figure S91).

**Table 8.** Comprehensive effects of GM-crops consumption on mammal liver functional indicator.

Indicators	Included articles number	Cohen’s *d* (95% CI)	*p*-value	Plant
ALT	26	−0.09 [−0.21, 0.04]	.17	Maize
AST	24	0.03 [−0.10, 0.16]	.65	Maize
TBIL	8	0.04 [−0.13,0.22]	.63	Maize
ALT	17	0.03 [−0.20, 0.26]	.81	Rice
AST	15	−0.11 [−0.37, 0.14]	.39	Rice
ALT	10	−0.03 [−0.16, 0.11]	.68	Soybean
AST	10	0.11 [−0.03, 0.25]	.12	Soybean

ALT: alanine aminotransferase; AST: aspartate transaminase; TBIL: total bilirubin.

## 4. Discussion

This study, through multi-source data correlation analysis, systematically summarized the impact of consuming three types of GM crops on the physiological indicators data set of mammals, and thus revealed the comprehensive impact of consuming GM crops on human health status. Our results suggested that the consumption of GM maize, rice, and soybean might have no pathological effect on human health status. However, certain physiological indicators responded significantly to GM crops consumption were identified when consuming specific GM crops. These indicators were the relative weights of liver with non-nutritional GM maize consumption, the serum GLU concentration with nutritionally changed GM maize consumption and the relative kidney weight with nutritionally changed GM rice consumption. Although these physiological indicators were observed to respond to the consumption of GM crops, evidences were insufficient to consider these responses as health risks for mammals^13,28,29,74^ (Experimental Center for Life Science, 2021).^73^

Upon consuming GM-maize, it was observed that the relative liver weight in female mammals tends to be higher, and the data from two researches mainly contributed to the difference between the GM maize group and the non-GM maize group.^28,29^ However, it should be noted that the control maize used in these two studies was not the strict parental line of the GM maize but rather a closely related variety, which may have influenced the results to some extent. Despite this, no abnormal hepatic pathological changes in the liver were revealed in histopathology examinations at the tissue level from the two studies. As for other analyzed studies, except aged model animals,^44^ no abnormal hepatic pathological changes were observed as well. Considering the complex influence of aging, there is no concrete evidence supporting the harmful influence of GM maize consumption on the liver or overall health. Additionally, GLU levels were found higher with medium and high doses of nutritionally changed GM maize consumption, which contains elevated levels of α-tocopherol and phytase. These components may contribute to the observed differences, as α-tocopherol is known to improve insulin sensitivity through its antioxidant properties, while phytase enhances nutrient availability by breaking down phytic acid,^71,72^ potentially influencing glucose metabolism. Meanwhile, the GM group reported a range of 4.3 ~ 12.61 mmol/L, and the non-GM group reported a range of 4.2 ~ 11.83 mmol/L (Figure S57) (Experimental Center for Life Science, 2021).^73^ Despite the slight increase in both lower and upper bounds, the fluctuation of GLU levels is substantial, suggesting that a minor increase in GLU concentration might not significantly impact mammalian health.^74^

Regarding the GM-rice group, an increase in kidney relative weight was noted, which was also related to consuming the medium and high doses of nutritionally changed GM rice. It is important to note, however, that in all studies contributing to these findings, the control rice used was not the direct parental line of the GM rice but instead a closely related variety, which may have partially influenced the outcomes. Meanwhile, since this increase has not caused abnormal renal lesions, GM rice consumption could not be considered a health risk.^13,62^ Especially, for the lung relative weight, RBC concentration, and serum Cl^−^ concentration, these were physiological indicators that showed significant difference between GM rice group and non-GM rice group, however, these indicators might not be considered responding to GM rice consumption since without strict dose dependence (ICCF, 2019).^75^ Meanwhile, more evidence is required for varied dosages to confirm the overall consequences of GM rice consumption in these indicators.

As for consuming GM soybean, our meta-analysis did not identify statistically significant alterations in any of the physiological indicators. However, the concentration of MON, TBIL, and electrolyte functional indicators and LDH were underrepresented in the current dataset due to the scarcity of the data of these indicators from the selected studies in this research. Thus, our future work will focus on acquiring high-quality datasets specifically for these underrepresented indicators. This targeted approach will enable a more complete and robust assessment of the potential health impacts associated with GM soybean consumption.

Methodologically, the results of the study might be influenced by literature acquisition and screening, as well as the bias in past experimental research concerning the selected model animals and the health indicators.^76,77^ The publications included in our analysis span 24 years from major databases and authoritative laboratory. Although we also attempted to retrieve raw experimental data from patent documents through major patent databases (e.g., USPTO, EPO, WIPO), patents typically disclose research outcomes and applications rather than complete raw data suitable for meta-analysis. Therefore, further integration of data from gray sources, including national agricultural departments and ministries, would expand the scope of the research. Further investigations are necessary to expand the scope of time, especially for rice and soybean, and the coverage of GM crop species. Meanwhile, the majority of studies on genetically modified crops have conducted 90-day (subchronic) toxicological assessments in accordance with standard international guidelines^78^ (OECD, 2018).^79^ However, we did identify a subset of studies employing either shorter (acute)^22,46,49,55^ or significantly longer (chronic) exposure periods.^12,19,21^ This indicates a gap in the toxicological research on GM crop safety, particularly concerning systematic studies of varying exposure durations on animal health. It suggests a need for further studies across multiple time scales, to generate data and conclusions under more realistic and complex exposure scenarios. These efforts should build upon existing data obtained within established safety assessment frameworks, to advance toward a more comprehensive and nuanced understanding of long-term dietary risk.

## 5. Conclusion

By synthesizing multi-source experimental data from 2000 to 2024 and overcoming limitations of disparate methodologies and small sample sizes, this study provides robust evidence addressing previous constraints of heterogeneous study designs. The findings in this study revealed that nutritionally changed GM maize consumption increased serum glucose levels, nutritionally changed GM rice consumption increased relative kidney weight, and *Cry1F/PAT*-expressing maize consumption increased relative liver weight in female rats; however, we identified these specific measurable physiological changes connected with no pathological changes. These findings not only demonstrate safety but also highlight important metabolic considerations that warrant further investigation to fully understand the biochemical implications of GM crop consumption.

The curated dataset and systematically defined physiological indicator framework presented in this study provide a transparent resource for comparative risk assessment of GM crops. These findings contribute to the growing body of evidence regarding the safety assessment of genetically modified crops and highlight the importance of distinguishing statistical significance from biological relevance in food safety evaluation.

## Supplementary Material

Supplementary Figure S80 to S84.docx

Supplementary Figure S50 to S59.docx

Supplementary Figure S26 to S31.docx

Supplementary Figure S32 to S38.docx

Supplementary Figure S45 to S49.docx

Supplementary Figure S42 to S44.docx

Supplementary Figure S12 to S25.docx

Supplementary Tables.docx

Supplementary Figure S1 to S11.docx

Supplementary Figure S85 to S91.docx

Supplementary Figure S60 to S79.docx

Supplementary Figure S39 to S41.docx
