# Supplementary material for: Comprehensive risk assessment revealed some physiological indicators responding to various GM-crop consumption
Source: GM Crops Food. 2025 Dec 19;17(1):2603726. doi: 10.1080/21645698.2025.2603726 (PMC12721096; doi:10.1080/21645698.2025.2603726)

**Indicators of cardiovascular function after GM crops consumption**

**Figure S80** Consuming GM maize showed no statistically significant impact on mammalian CHOL concentration.


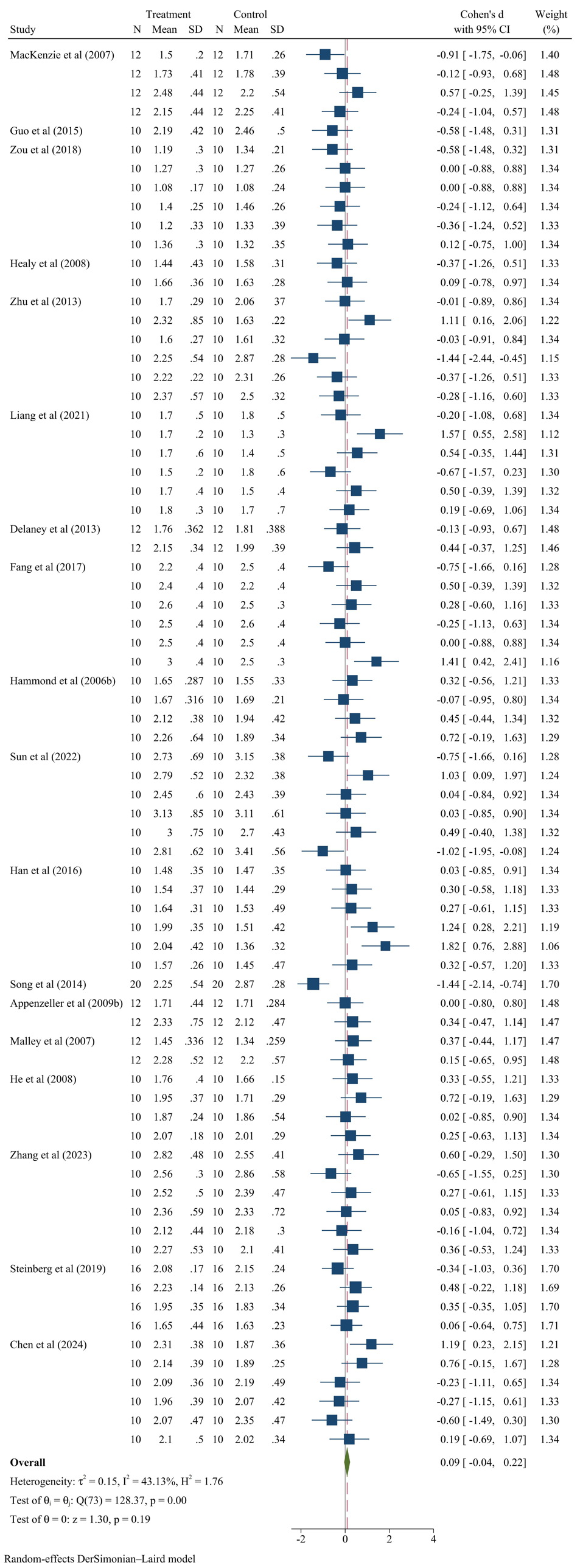


**Figure S81** Consuming GM soybean showed no statistically significant impact on mammalian CHOL concentration.


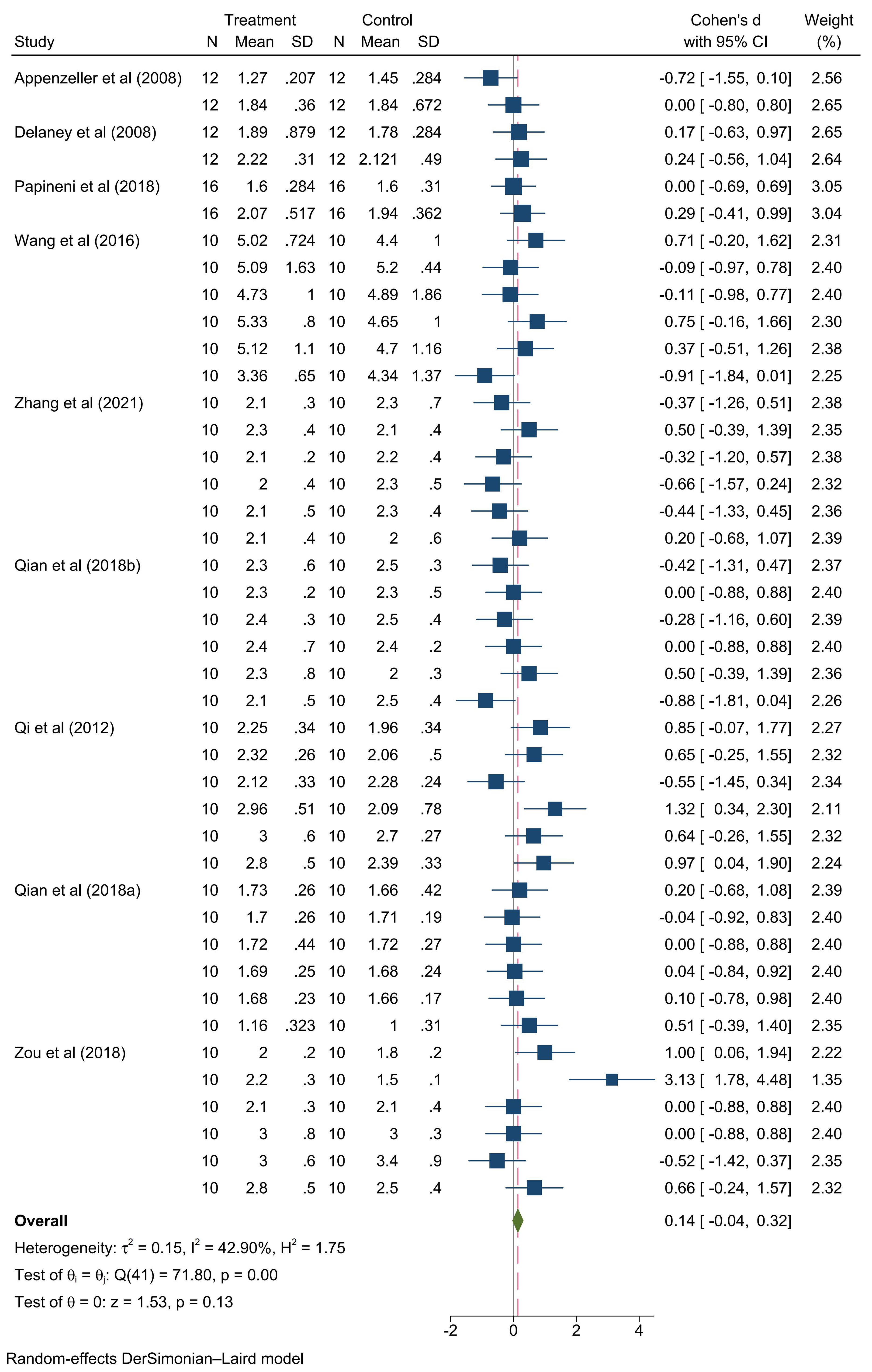


**Figure S82** Consuming GM rice showed no statistically significant impact on mammalian CHOL concentration.


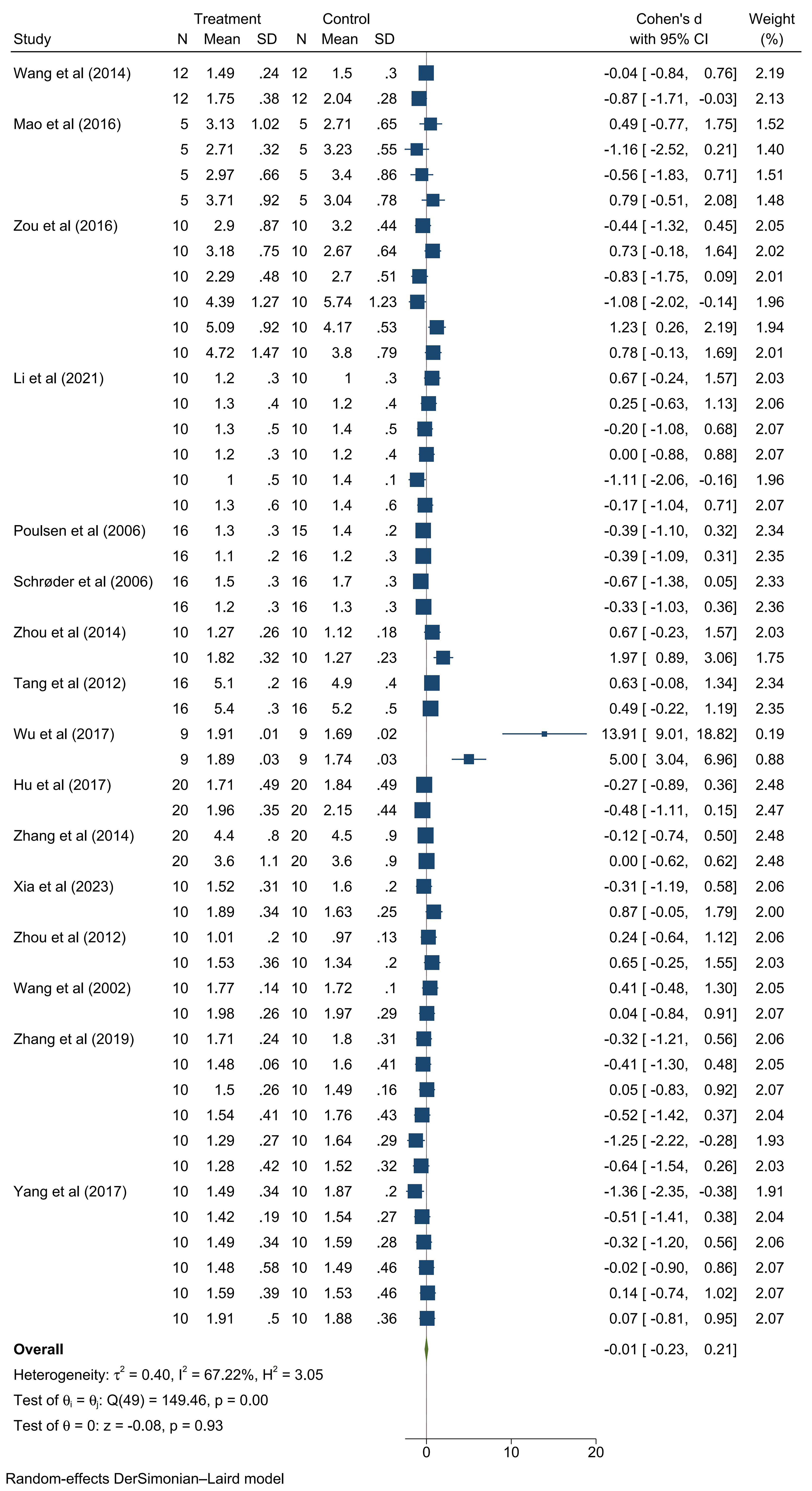


**Figure S83** Consuming GM maize showed no statistically significant impact on mammalian LDH concentration.


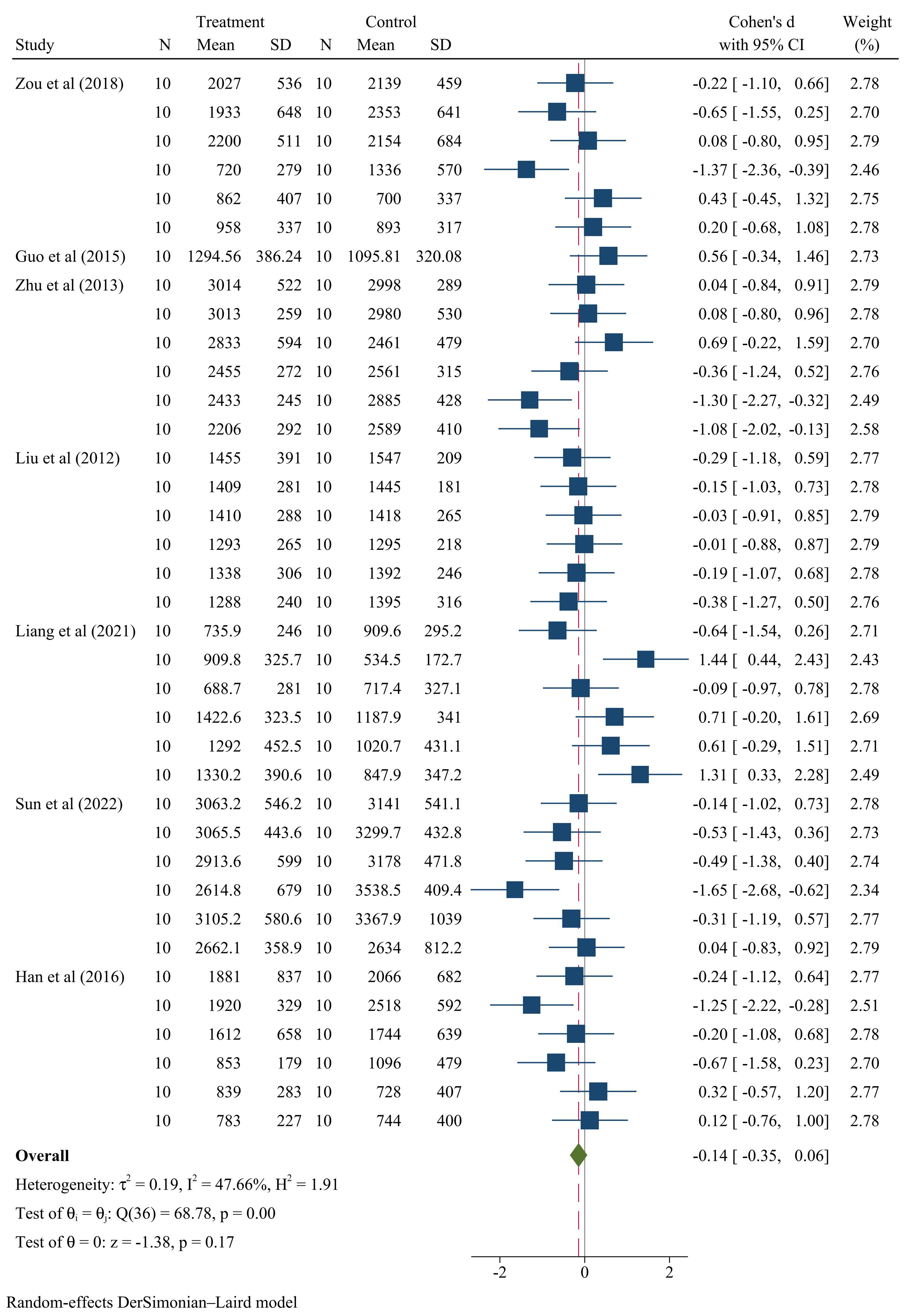


**Figure S84** Consuming GM rice showed no statistically significant impact on mammalian LDH concentration.


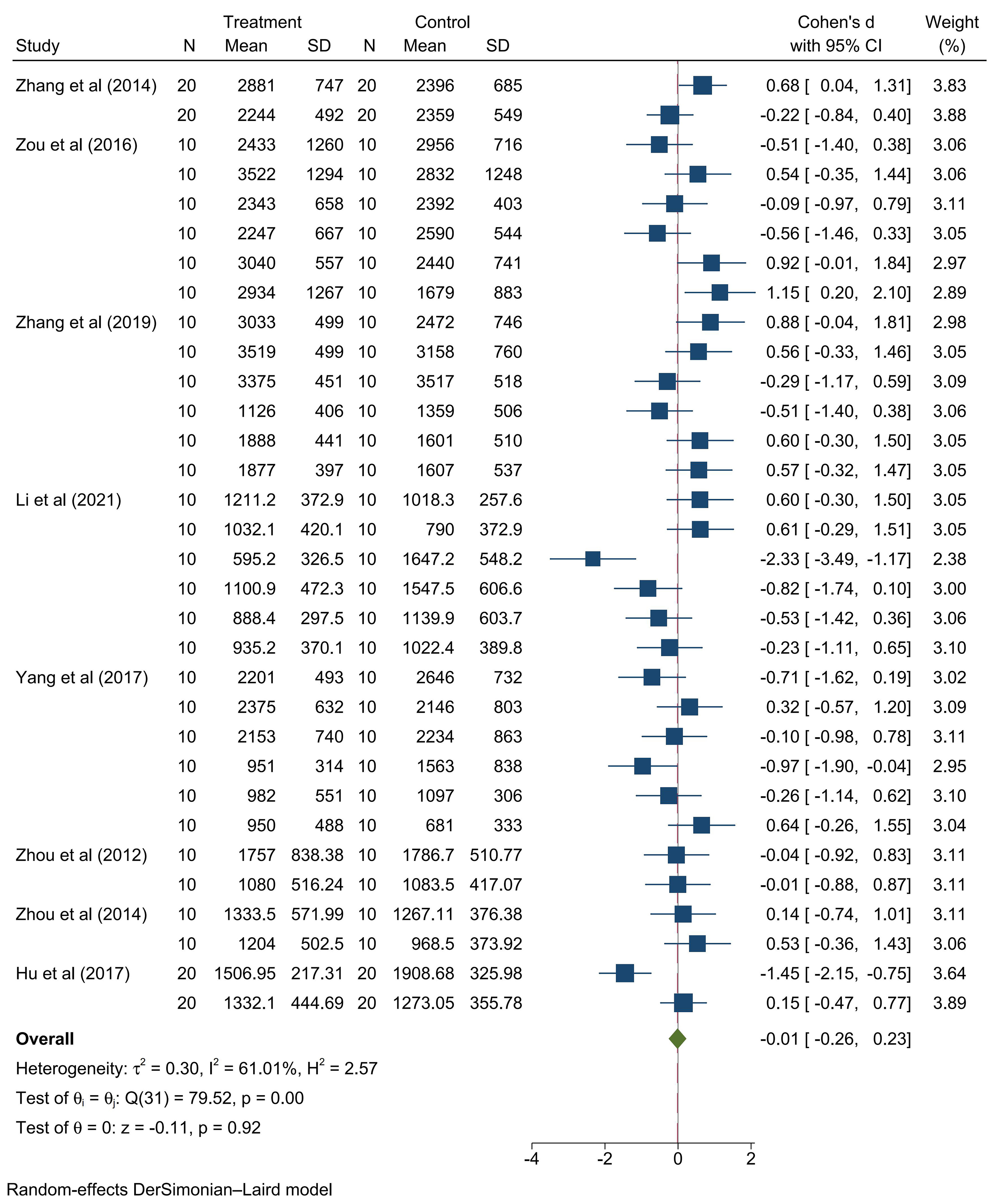

Supplement: Supplementary Figure S80 to S84.docx [file KGMC_A_2603726_SM6472.docx]
