# Supplementary material for: Comprehensive risk assessment revealed some physiological indicators responding to various GM-crop consumption
Source: GM Crops Food. 2025 Dec 19;17(1):2603726. doi: 10.1080/21645698.2025.2603726 (PMC12721096; doi:10.1080/21645698.2025.2603726)

**Electrolyte concentrations after GM-rice consumption**

**Figure S50** Consuming GM rice led to statistically significant decrease on mammalian serum Cl^-^ concentration.


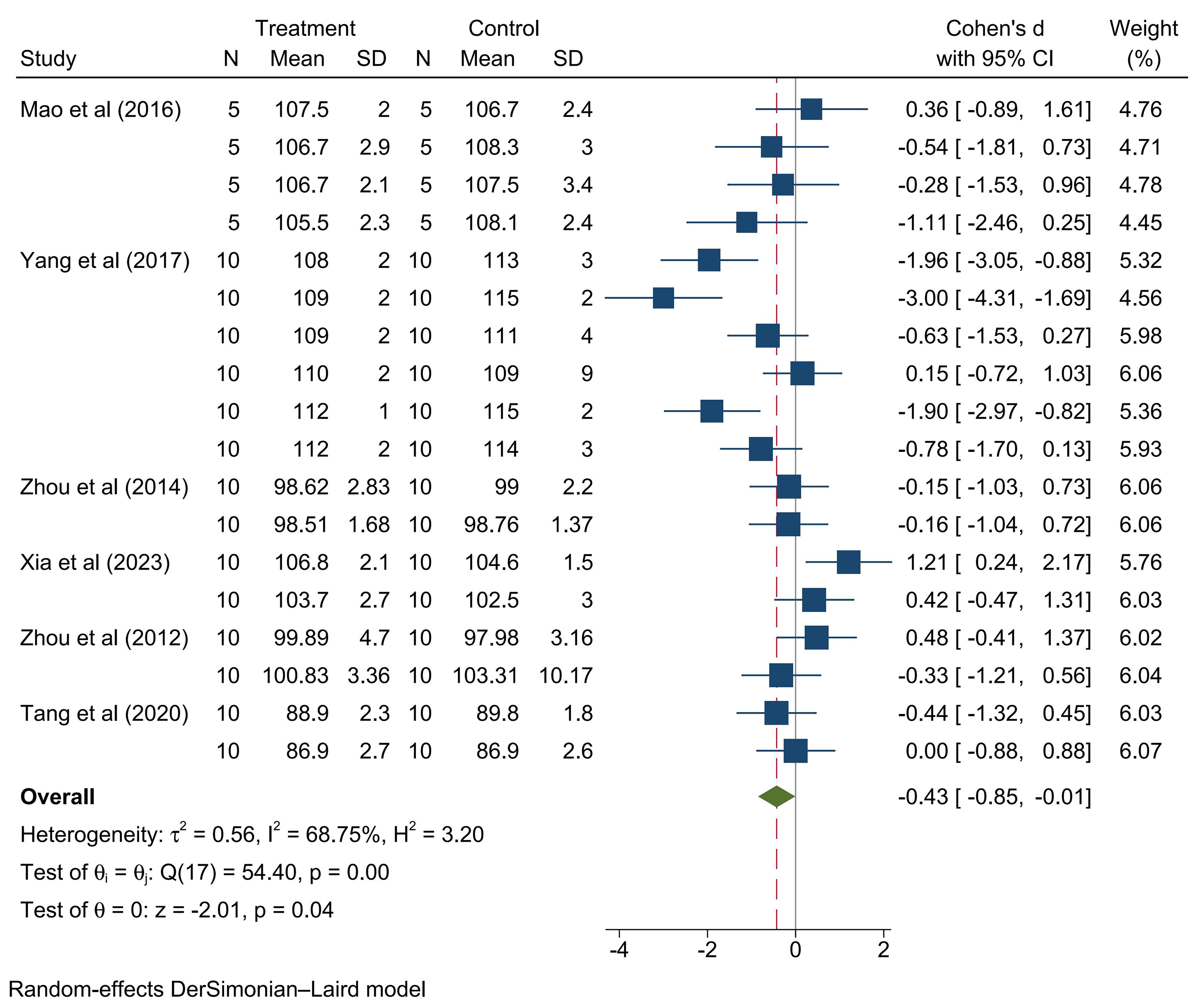


**Figure S51** Consuming low dose of GM rice showed no statistically significant impact on mammalian serum Cl^-^ concentration.


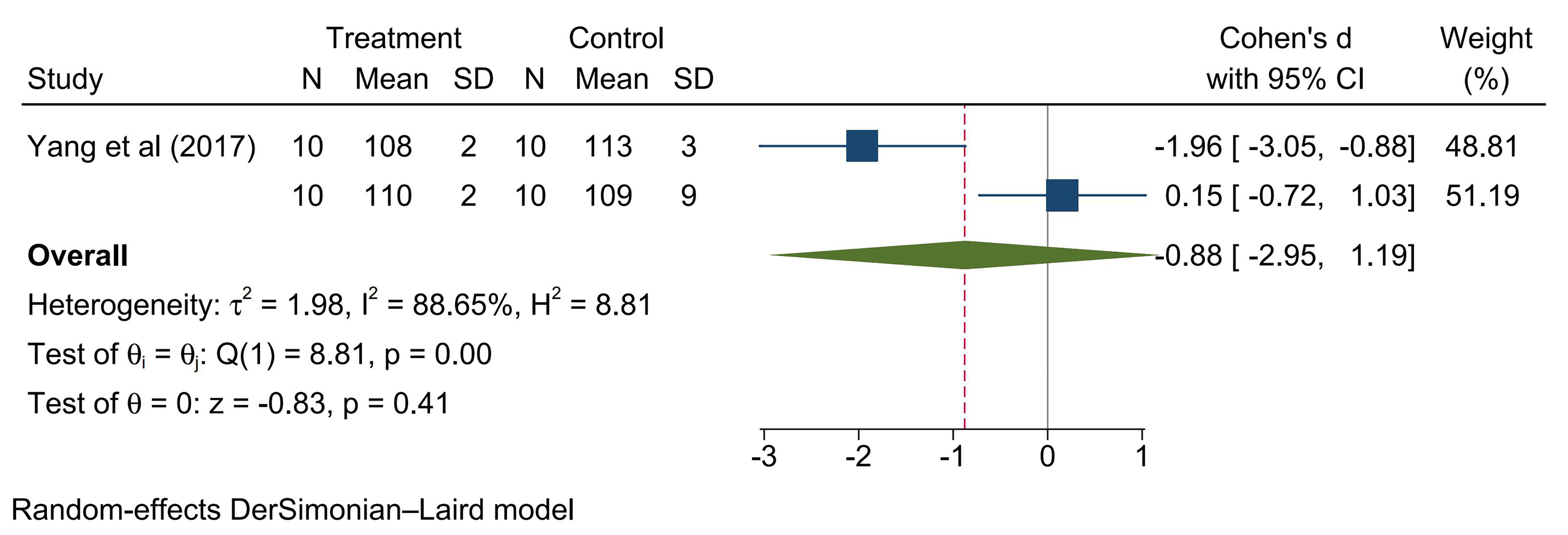


**Figure S52** Consuming medium dose of GM rice showed no statistically significant impact on mammalian serum Cl^-^ concentration.


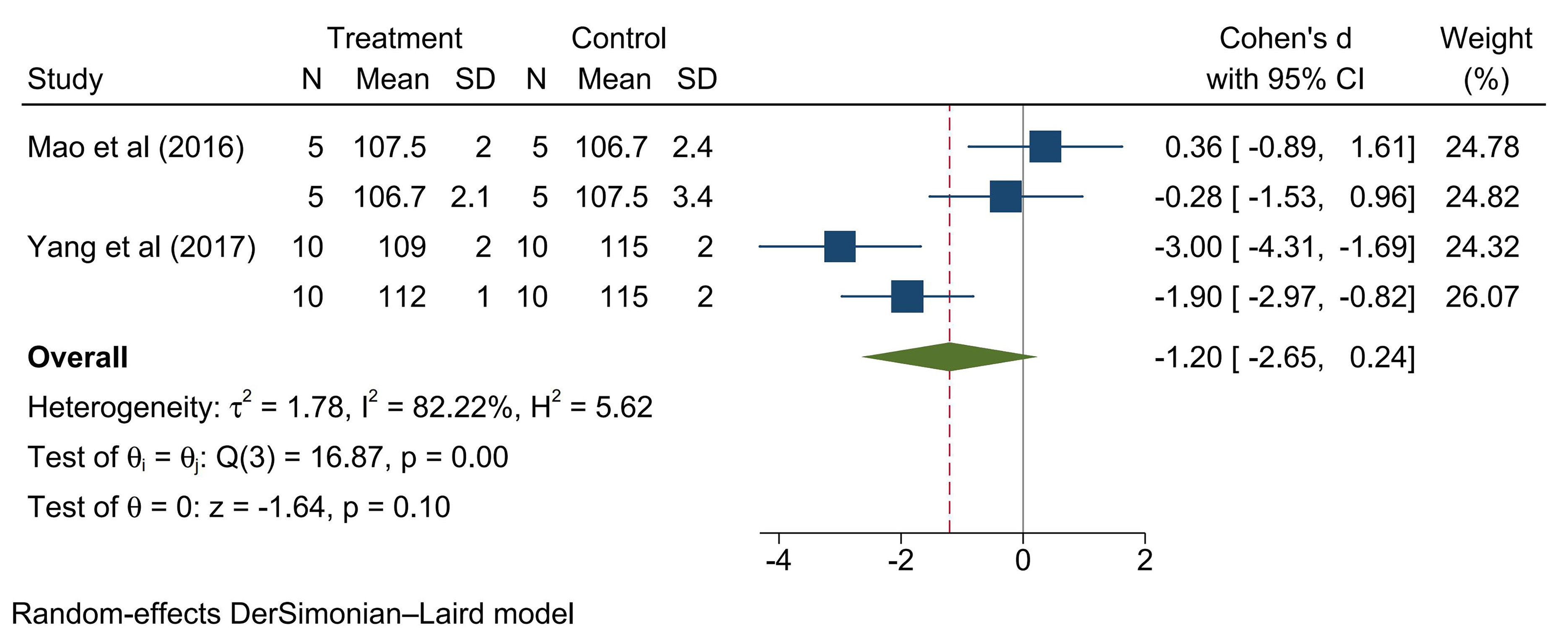


**Figure S53** Consuming high dose of GM rice showed no statistically significant impact on mammalian serum Cl^-^ concentration.


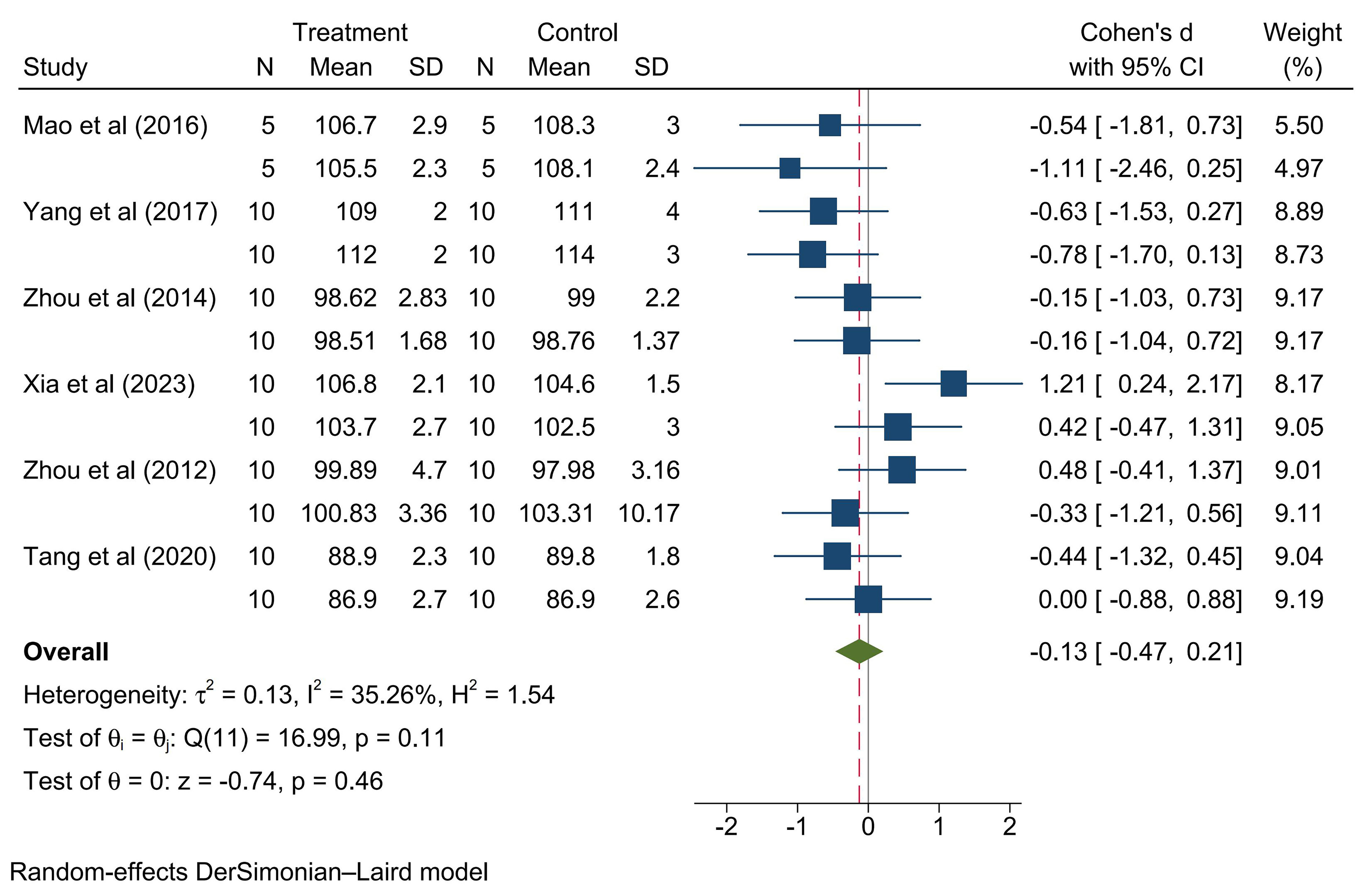


**Figure S54** Consuming GM rice showed no statistically significant impact on male mammalian serum Cl^-^ concentration.


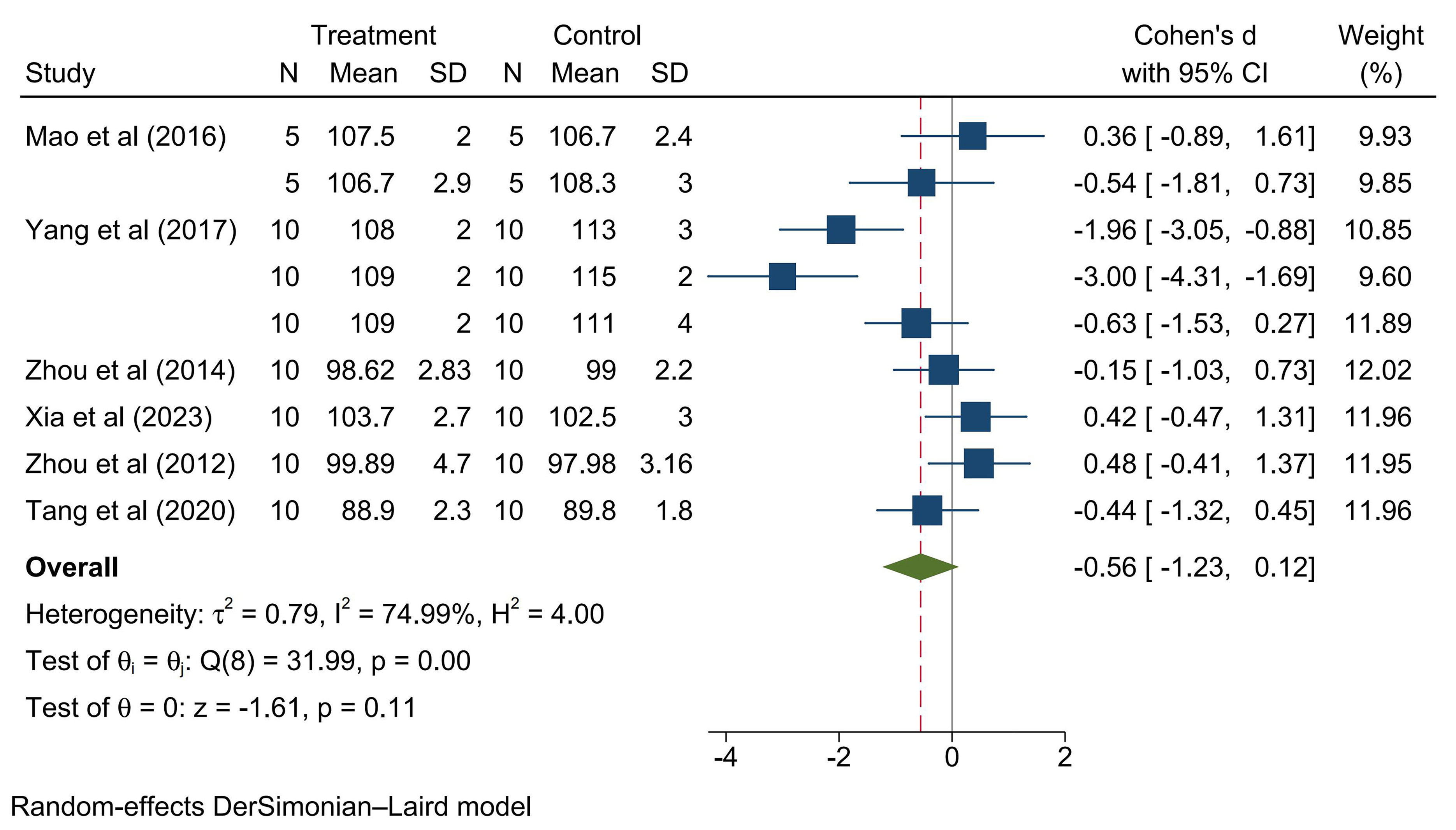


**Figure S55** Consuming GM rice showed no statistically significant impact on female mammalian serum Cl^-^ concentration.


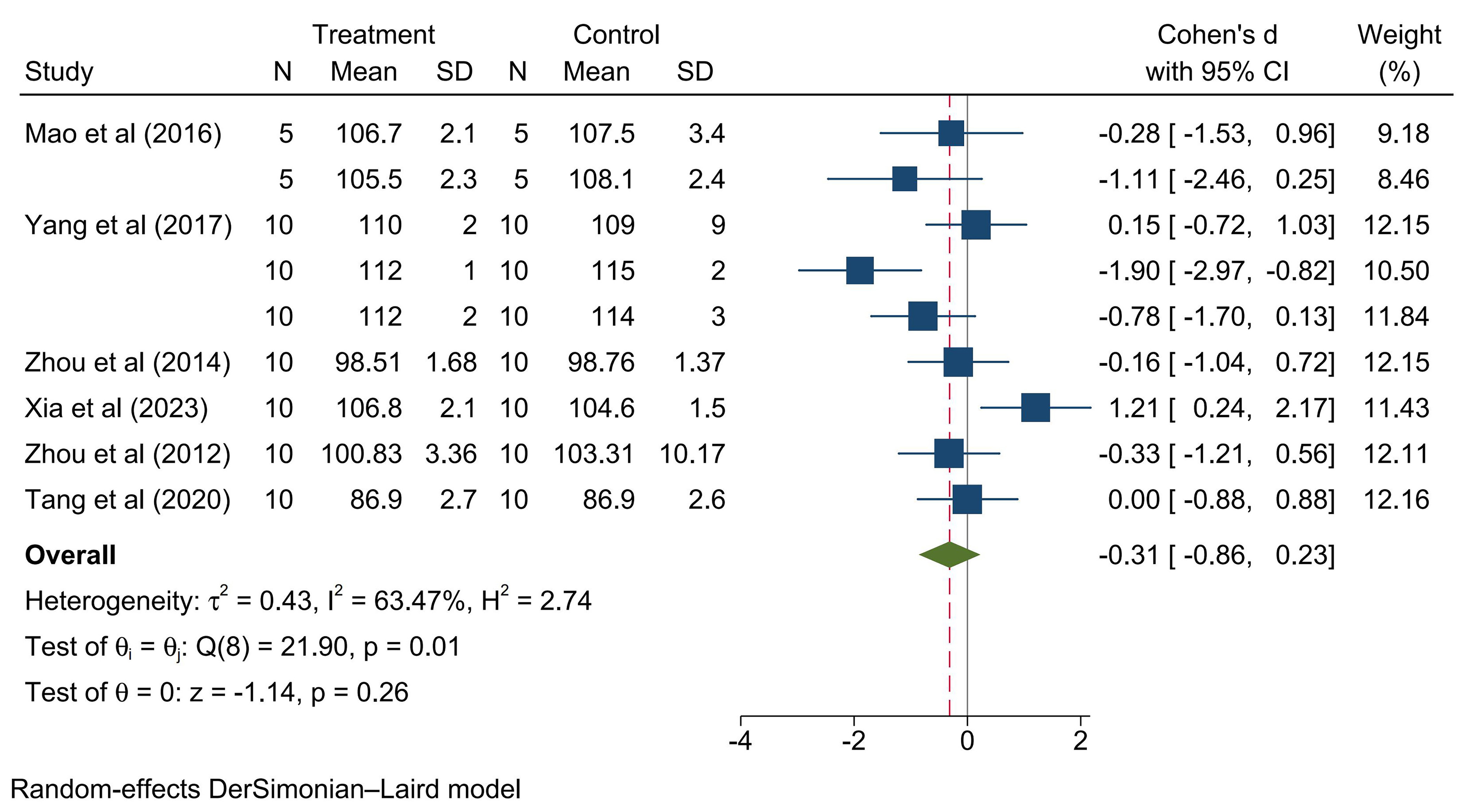


**Figure S56** Consuming GM rice showed no statistically significant impact on mammalian serum K^+^ concentration.


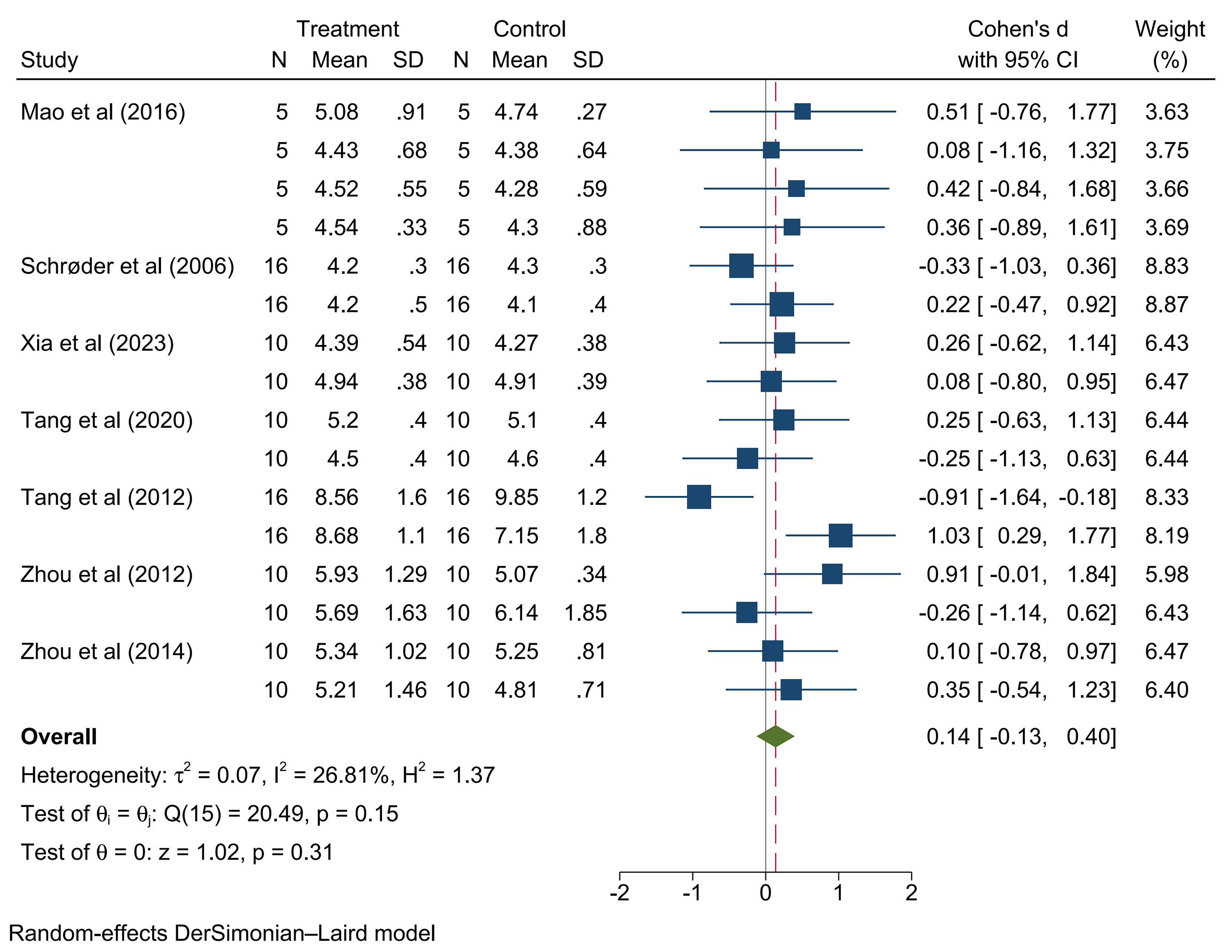


**Figure S57** Consuming GM rice showed no statistically significant impact on mammalian serum Na^+^ concentration.


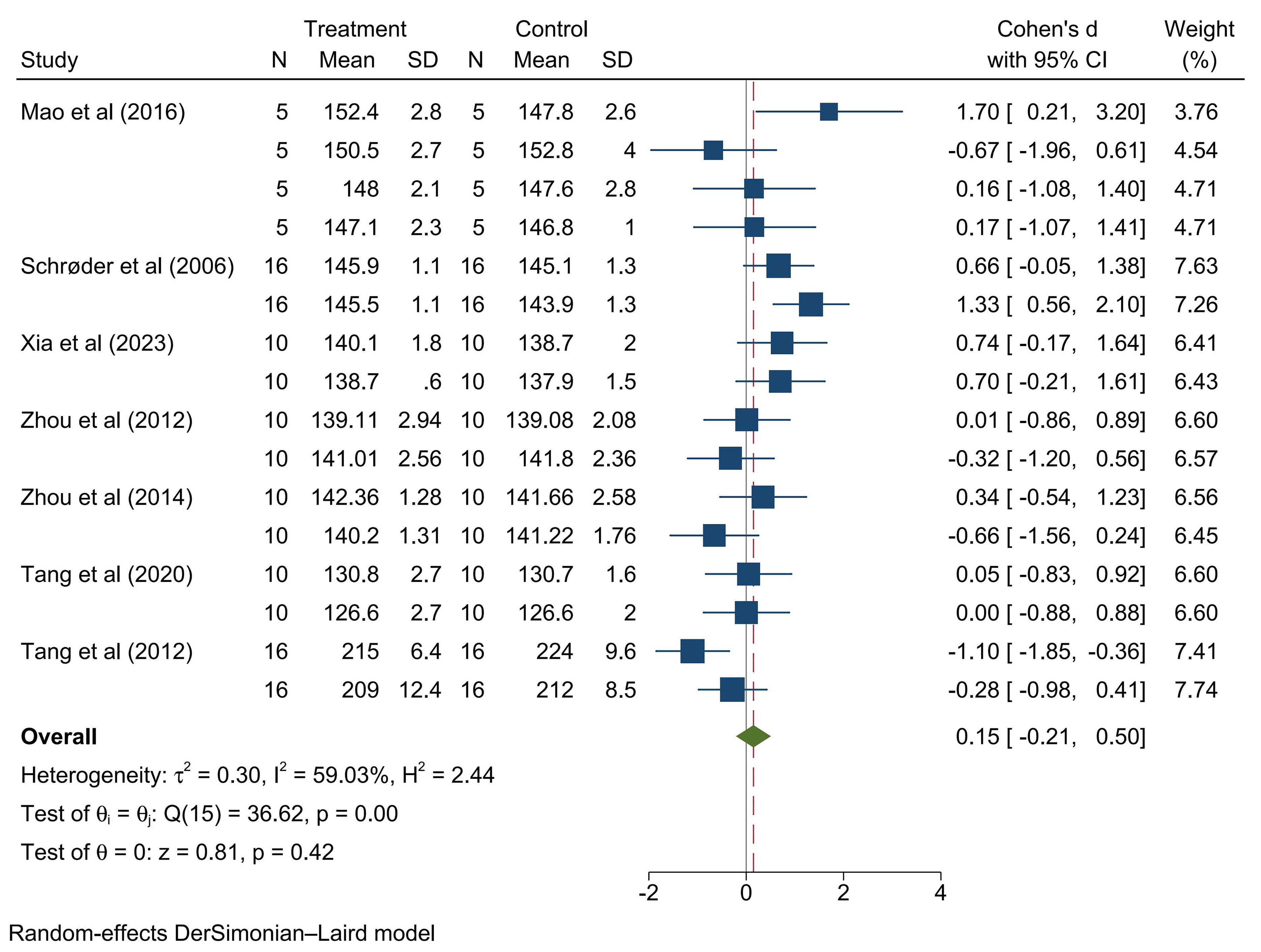


**Figure S58** Consuming GM rice showed no statistically significant impact on mammalian serum Ca^2+^ concentration.


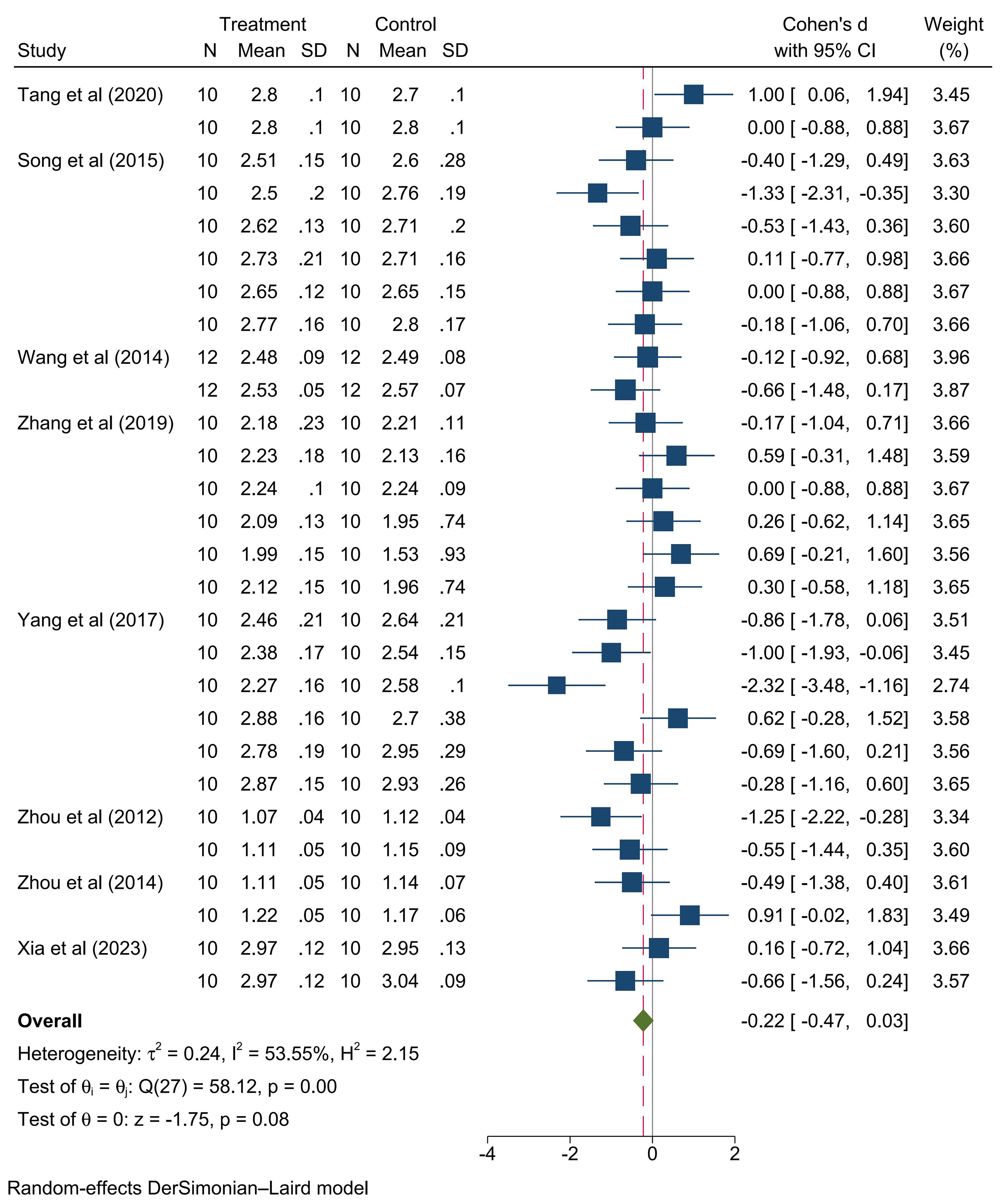


**Figure S59** Consuming GM rice showed no statistically significant impact on mammalian serum P concentration.


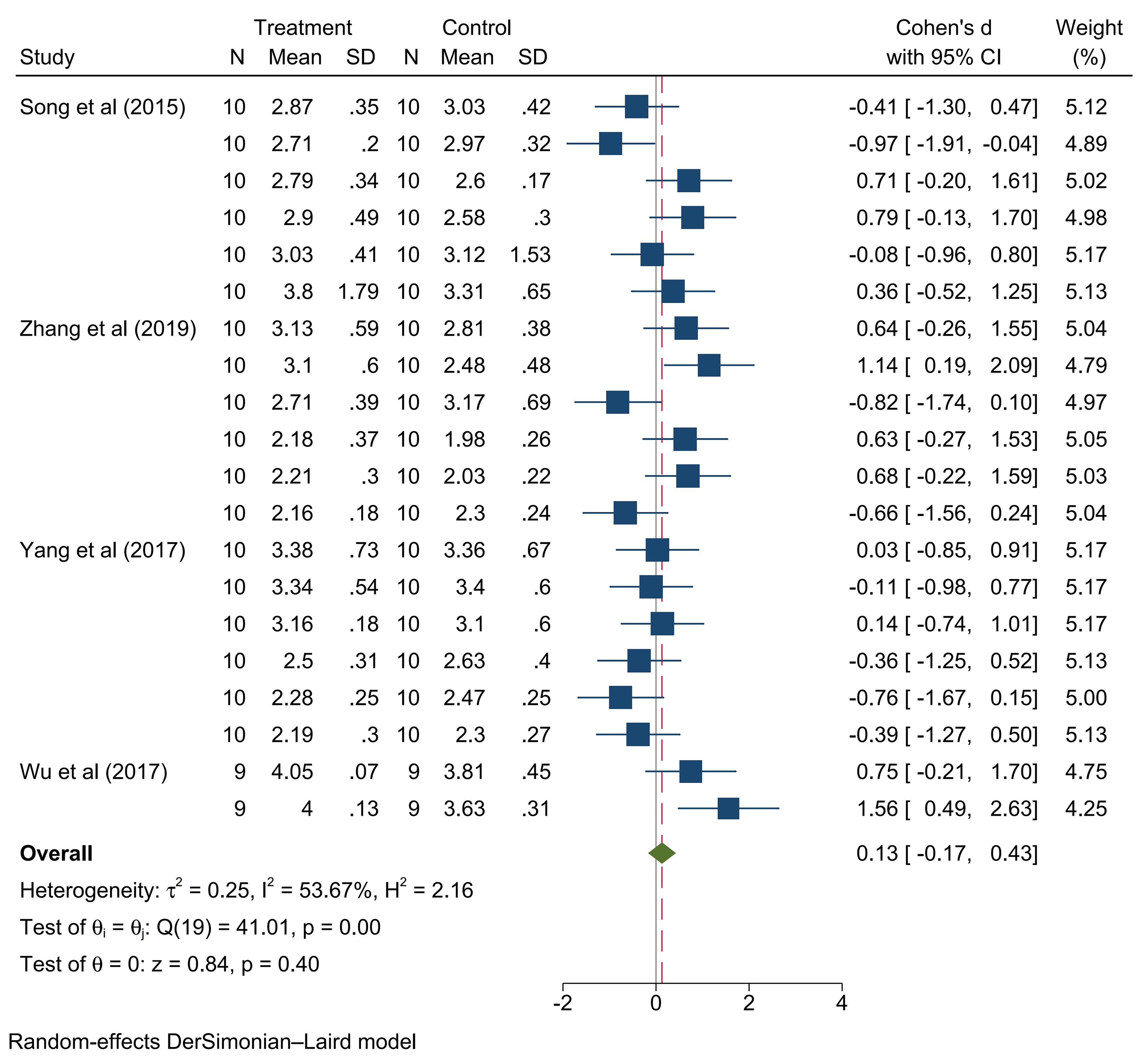

Supplement: Supplementary Figure S50 to S59.docx [file KGMC_A_2603726_SM6471.docx]
