# Supplementary material for: Comprehensive risk assessment revealed some physiological indicators responding to various GM-crop consumption
Source: GM Crops Food. 2025 Dec 19;17(1):2603726. doi: 10.1080/21645698.2025.2603726 (PMC12721096; doi:10.1080/21645698.2025.2603726)

**Relative organ weight after GM-soybean consumption**

**Figure S26** Consuming GM soybean showed no statistically significant impact on mammalian relative brain weight


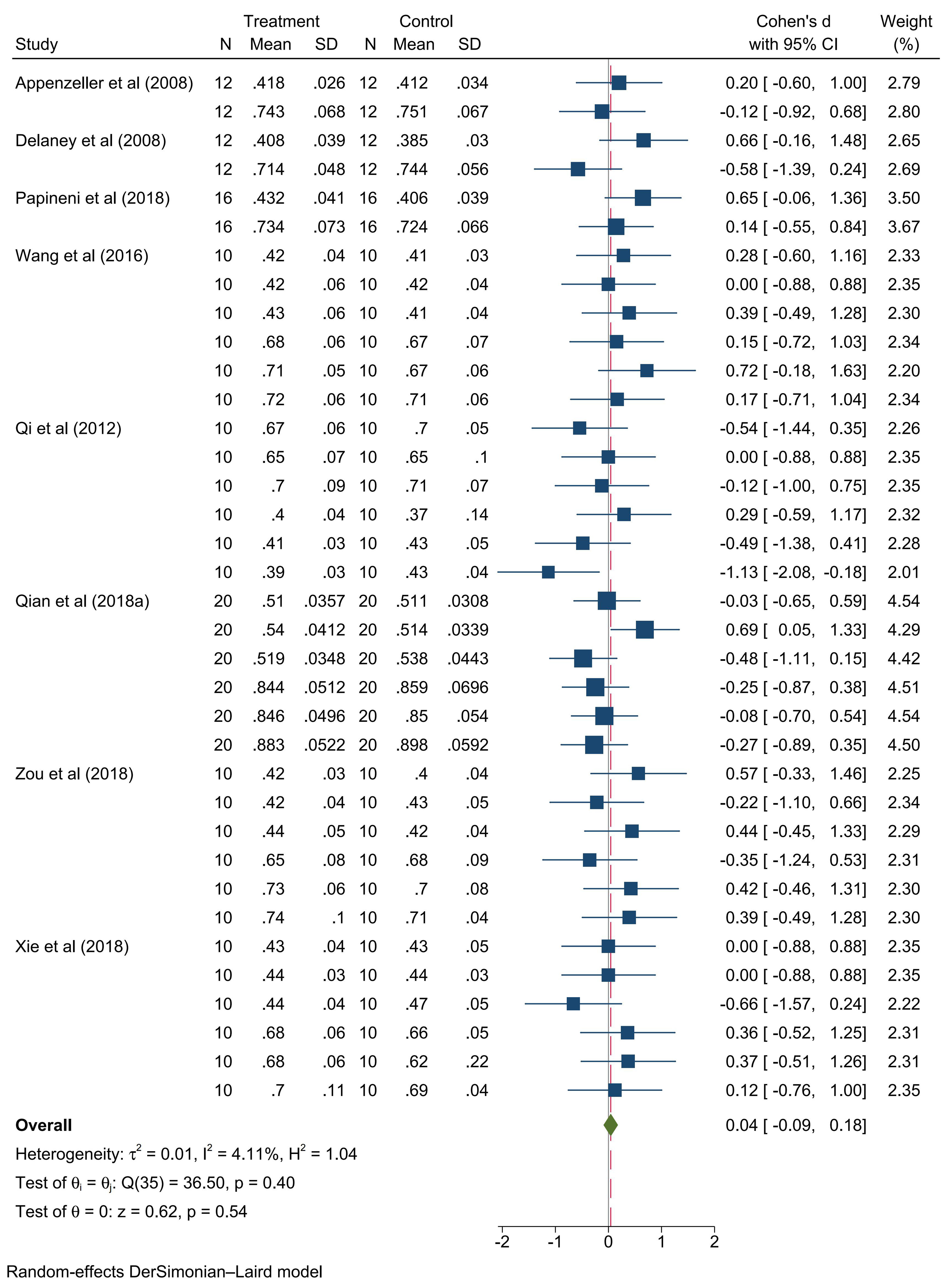


**Figure S27** Consuming GM soybean showed no statistically significant impact on mammalian relative kidney weight


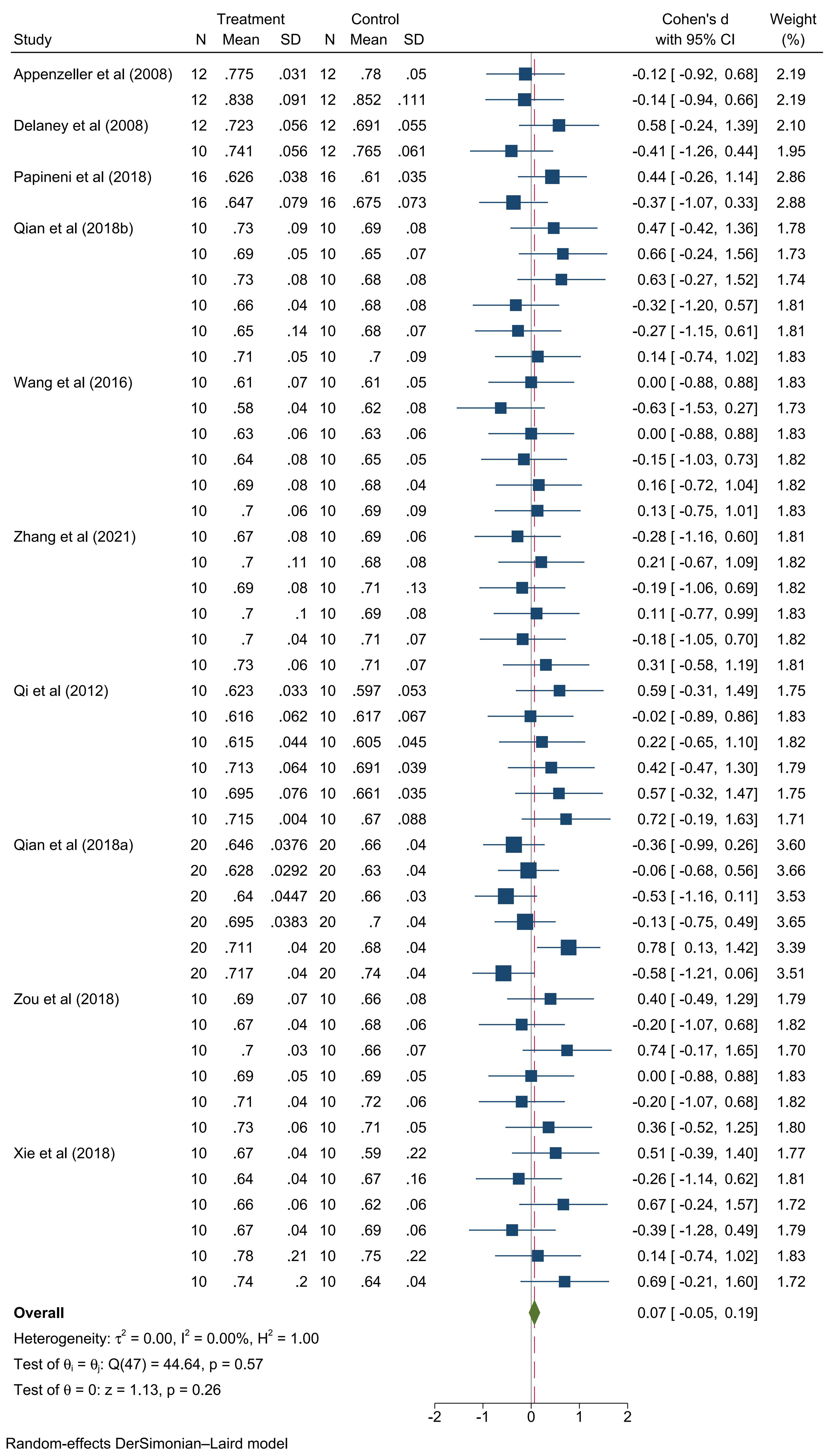


**Figure S28** Consuming GM soybean showed no statistically significant impact on mammalian relative liver weight


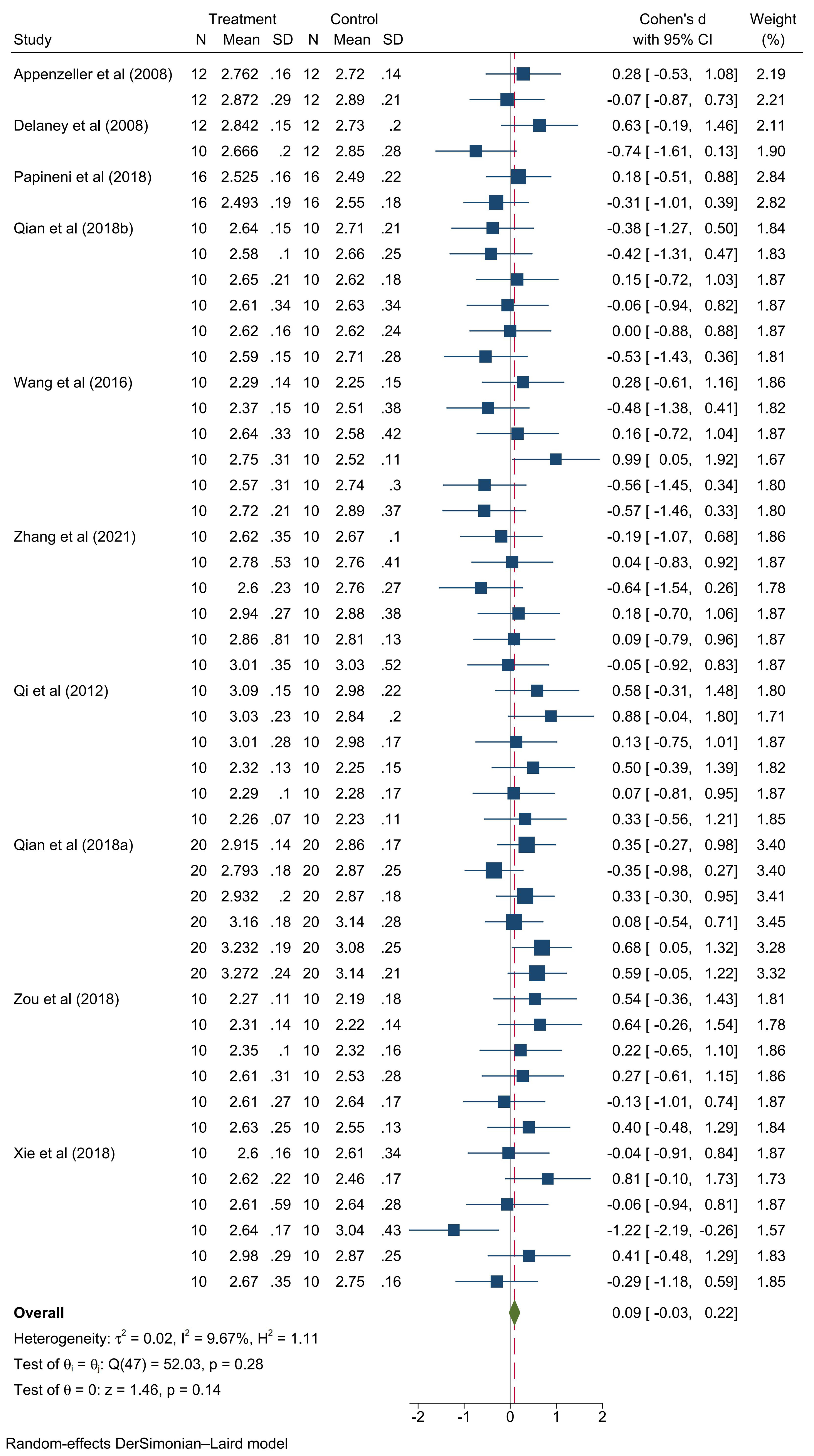


**Figure S29** Consuming GM soybean showed no statistically significant impact on mammalian relative spleen weight


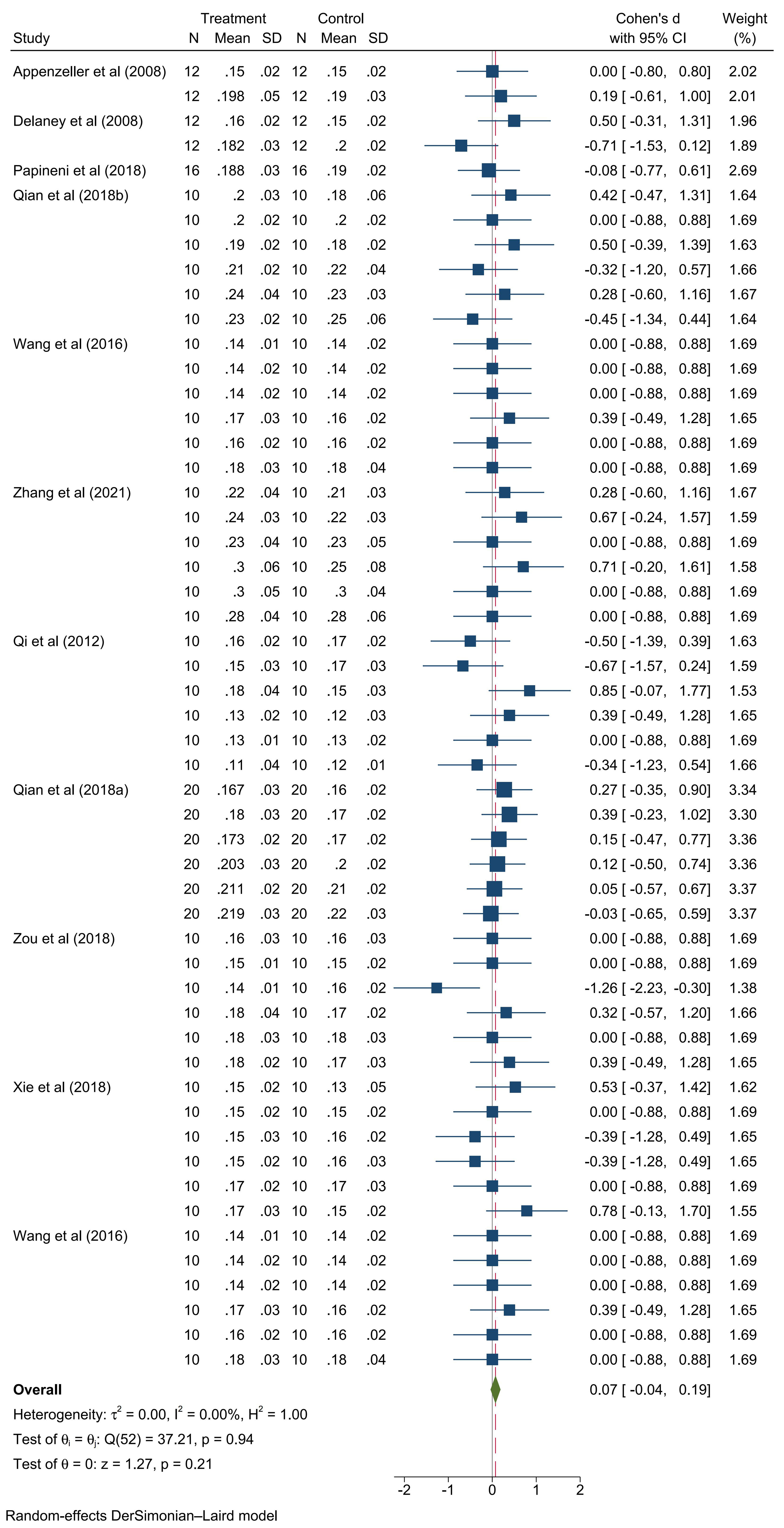


**Figure S30** Consuming GM soybean showed no statistically significant impact on mammalian relative heart weight


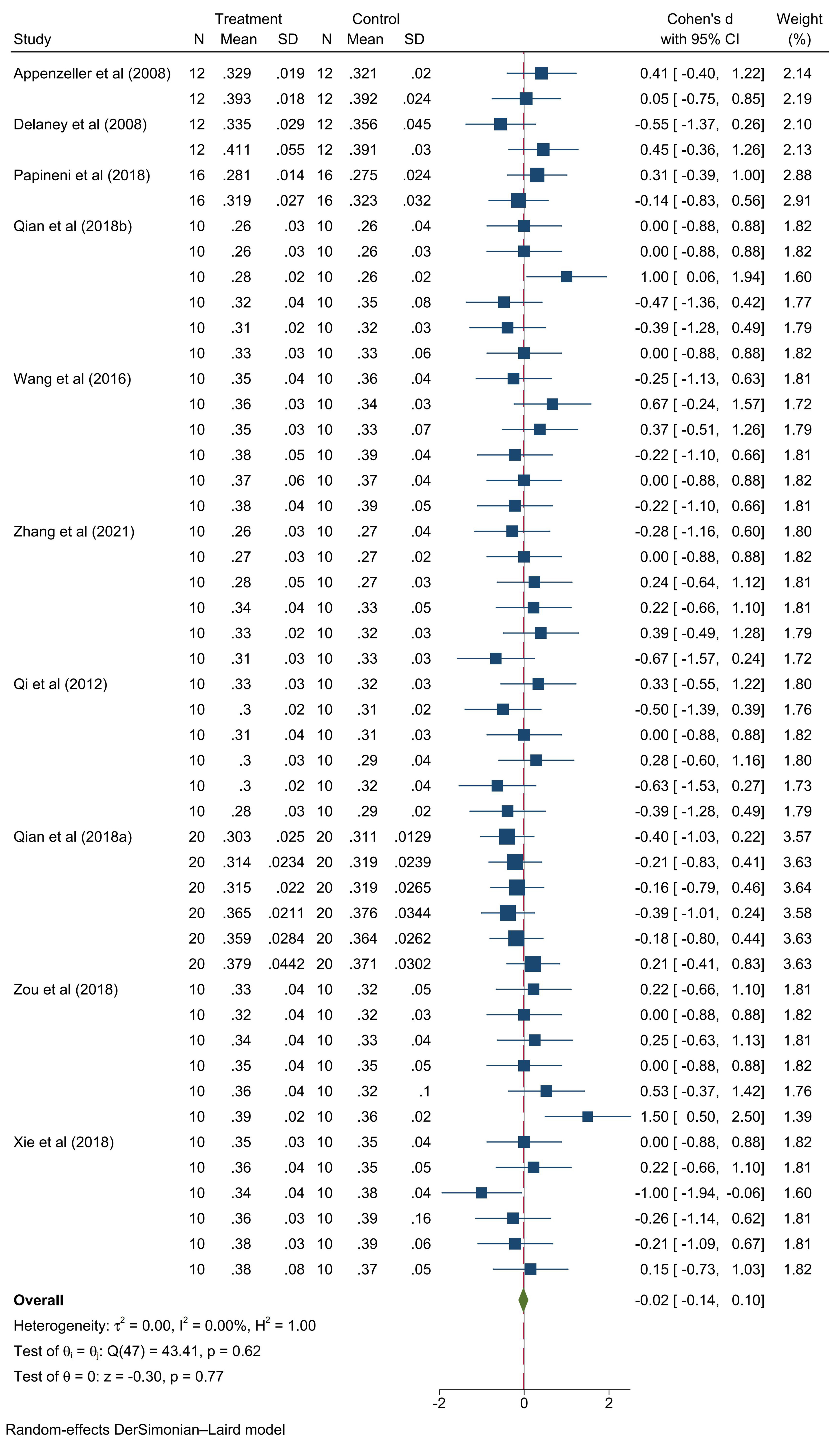


**Figure S31** Consuming GM soybean showed no statistically significant impact on mammalian relative lung weight


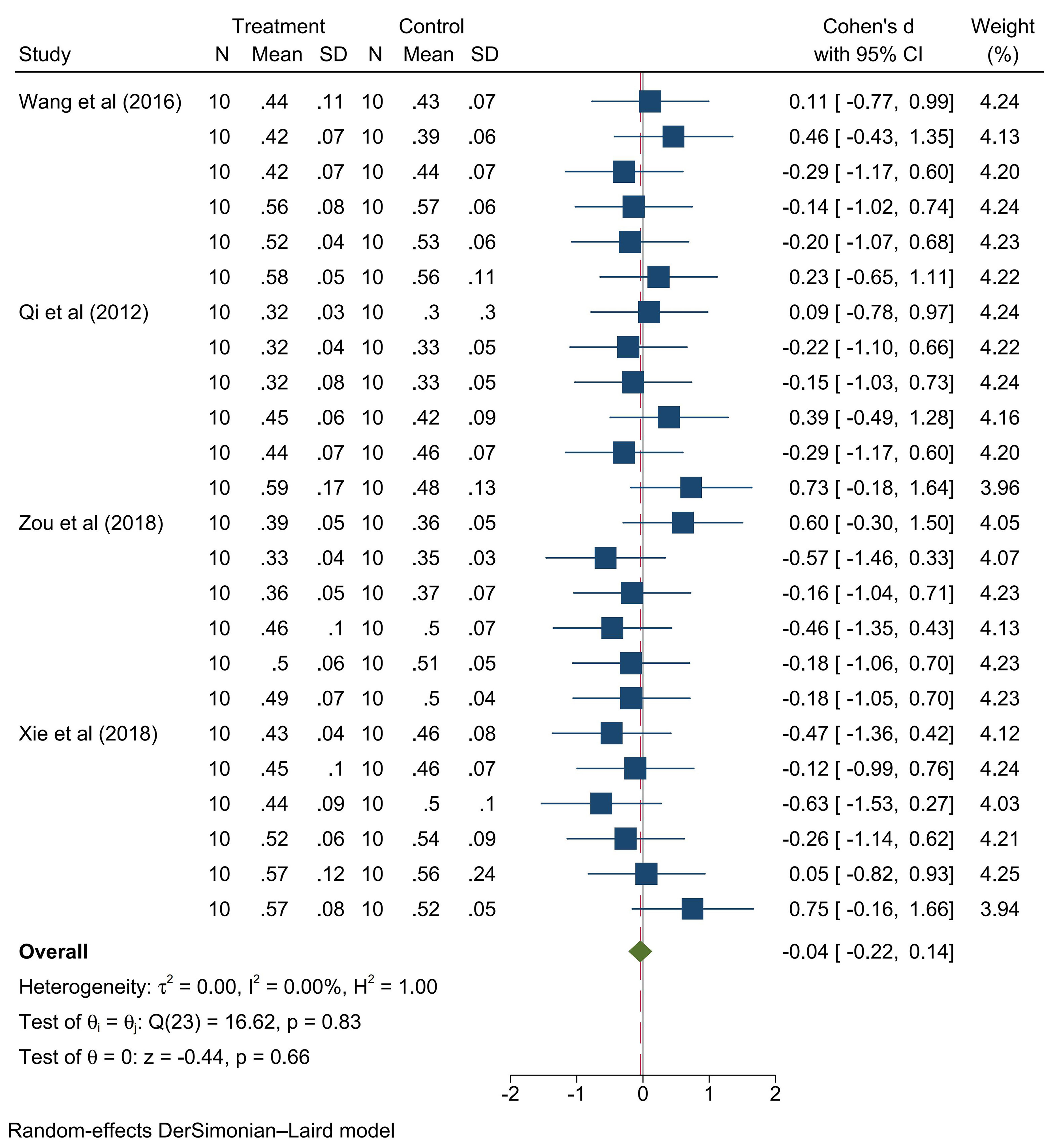

Supplement: Supplementary Figure S26 to S31.docx [file KGMC_A_2603726_SM6470.docx]
