# Supplementary material for: Comprehensive risk assessment revealed some physiological indicators responding to various GM-crop consumption
Source: GM Crops Food. 2025 Dec 19;17(1):2603726. doi: 10.1080/21645698.2025.2603726 (PMC12721096; doi:10.1080/21645698.2025.2603726)

**Indicators of renal function after GM-maize consumption**

**Figure S32** Consuming GM maize showed no statistically significant impact on mammalian BUN concentration.


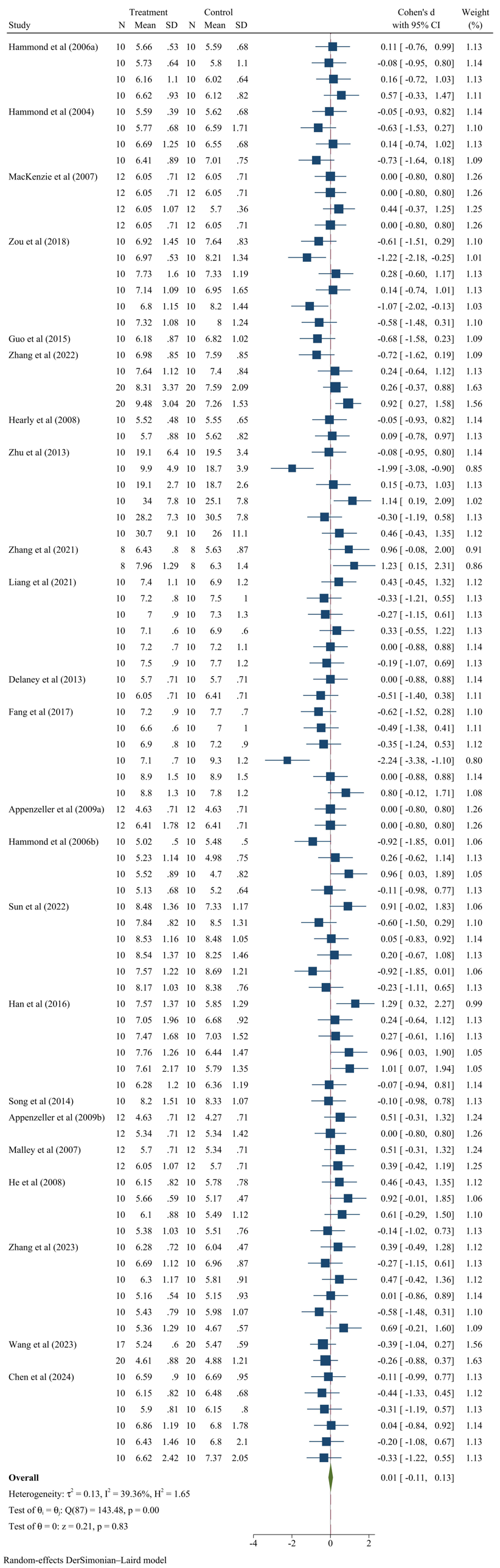


**Figure S33** Consuming GM maize showed no statistically significant impact on mammalian CRE concentration.


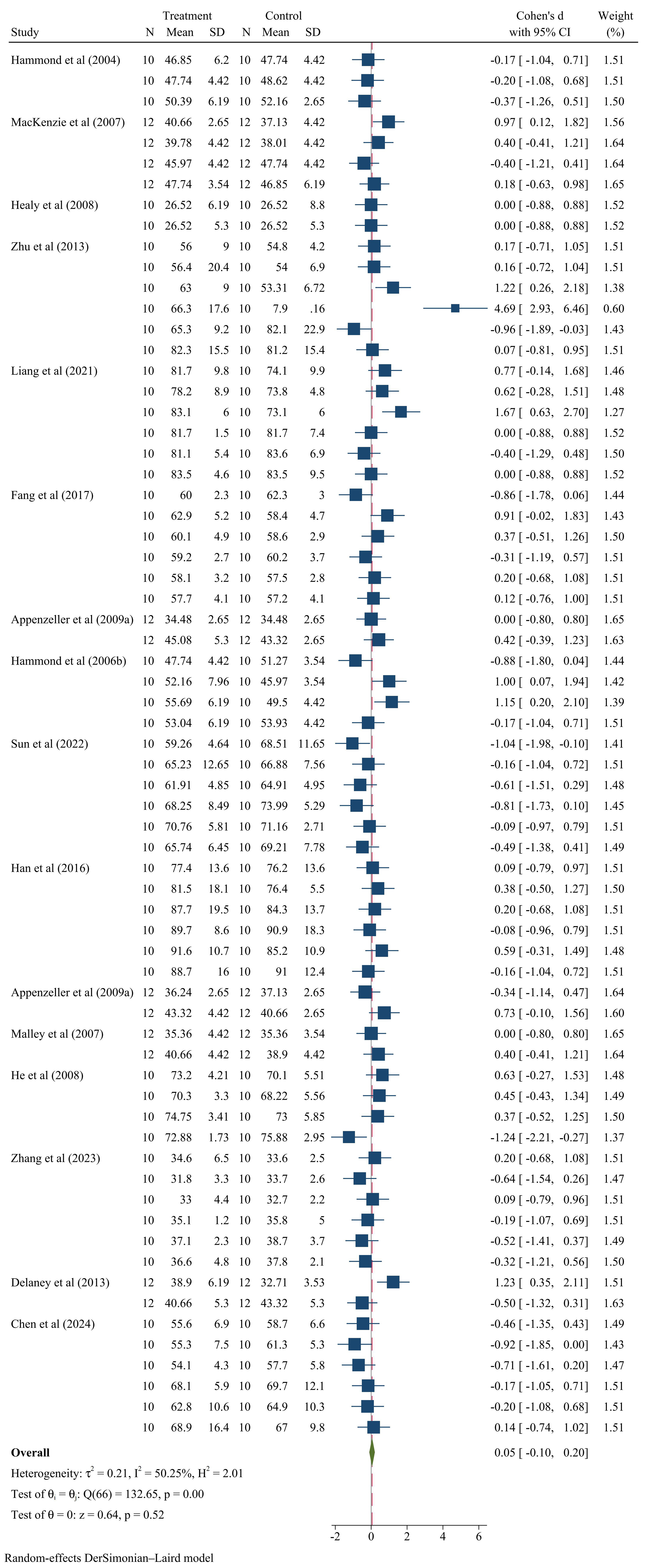


**Figure S34** Consuming GM maize led to statistically significant increase on mammalian GLU concentration.


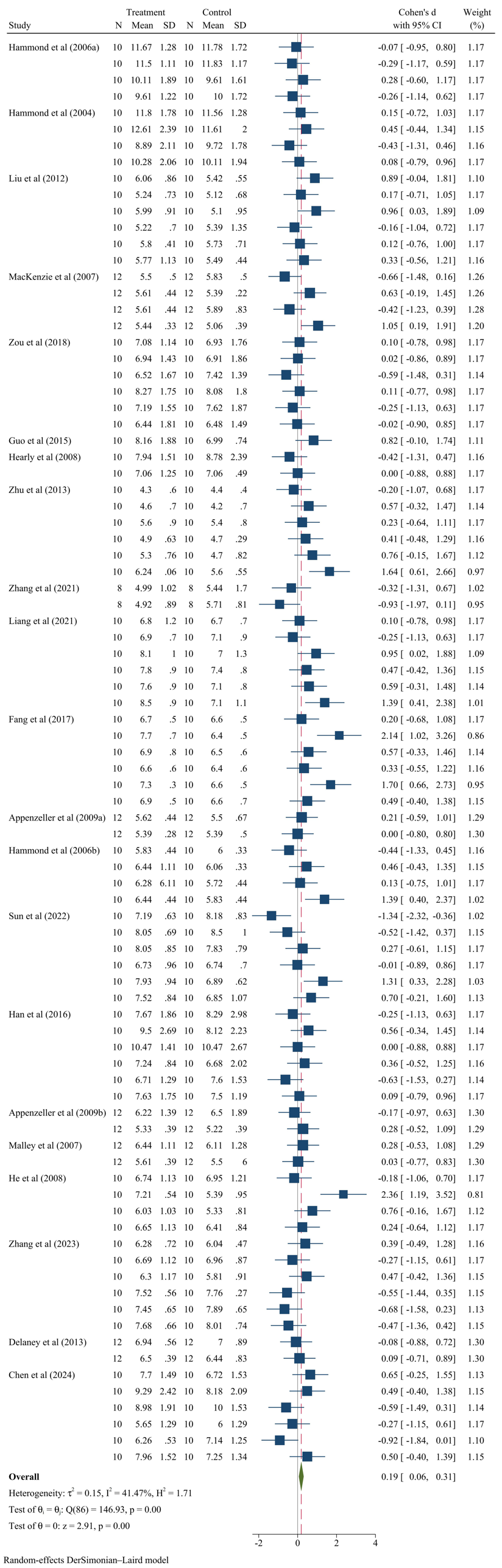


**Figure S35** Consuming low dose of GM maize showed no statistically significant impact on mammalian GLU concentration.


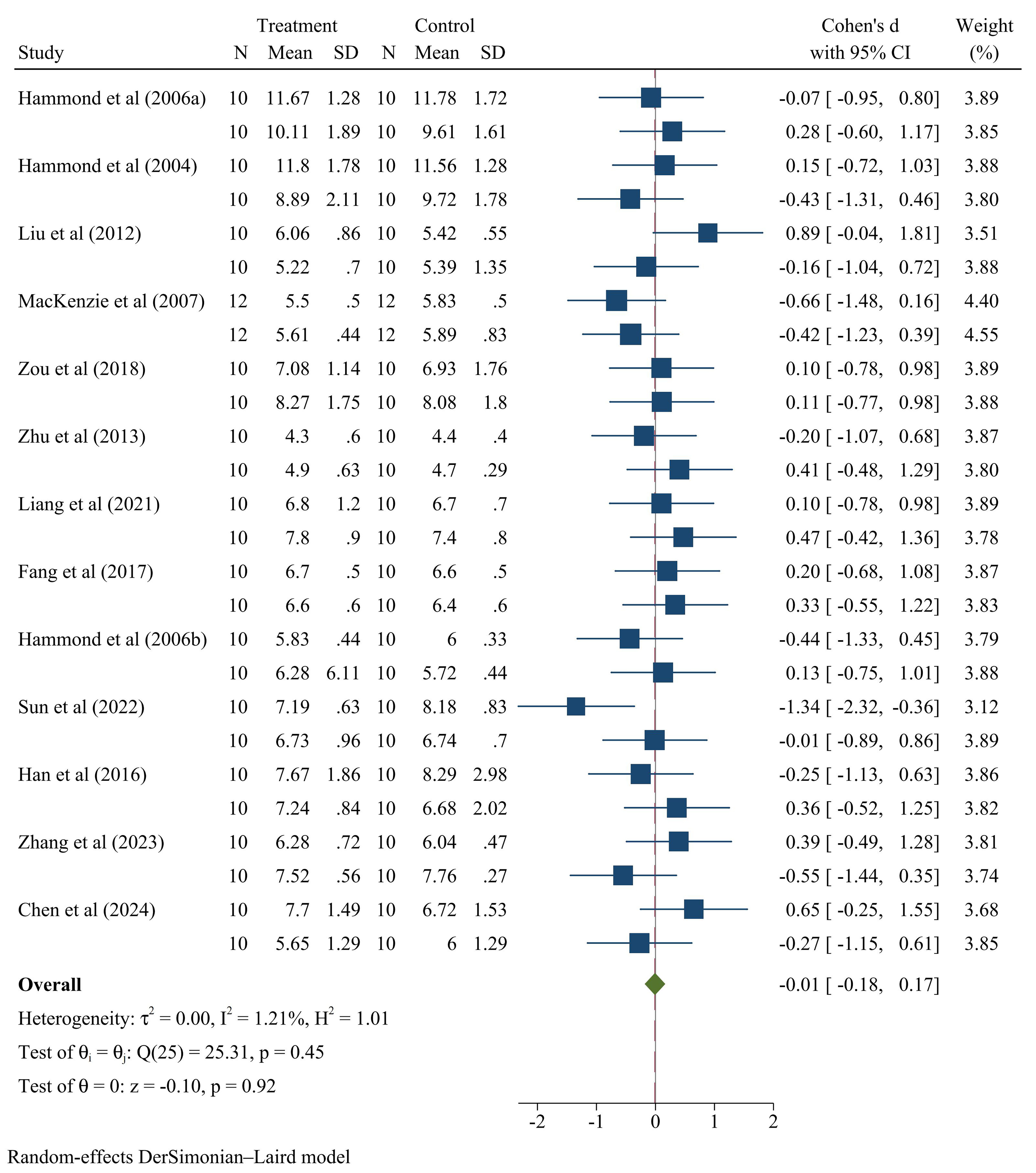


**Figure S36** Consuming medium dose of GM maize led to statistically significant increase on mammalian GLU concentration.


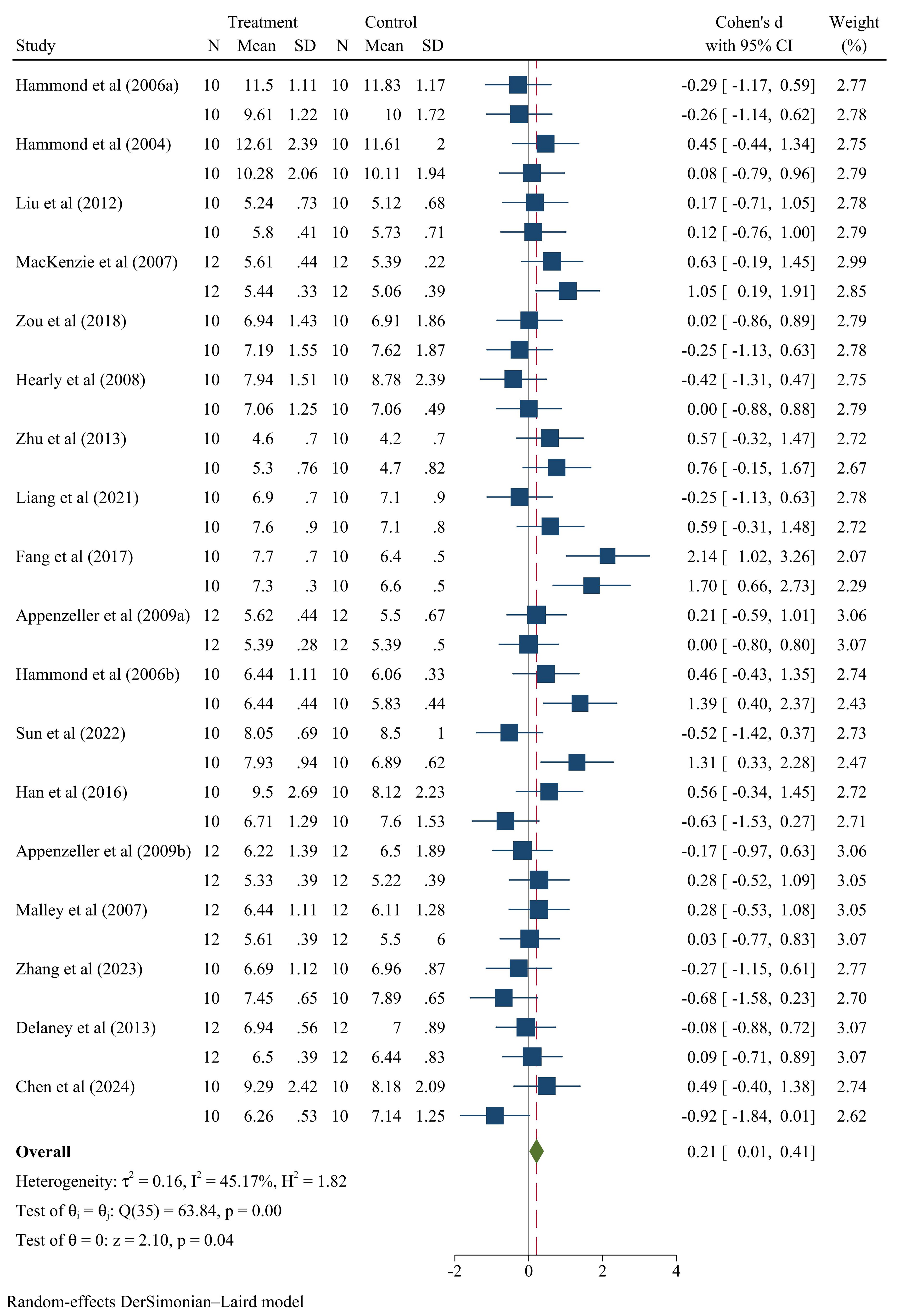


**Figure S37** Consuming high dose of GM maize led to statistically significant increase on mammalian GLU concentration.


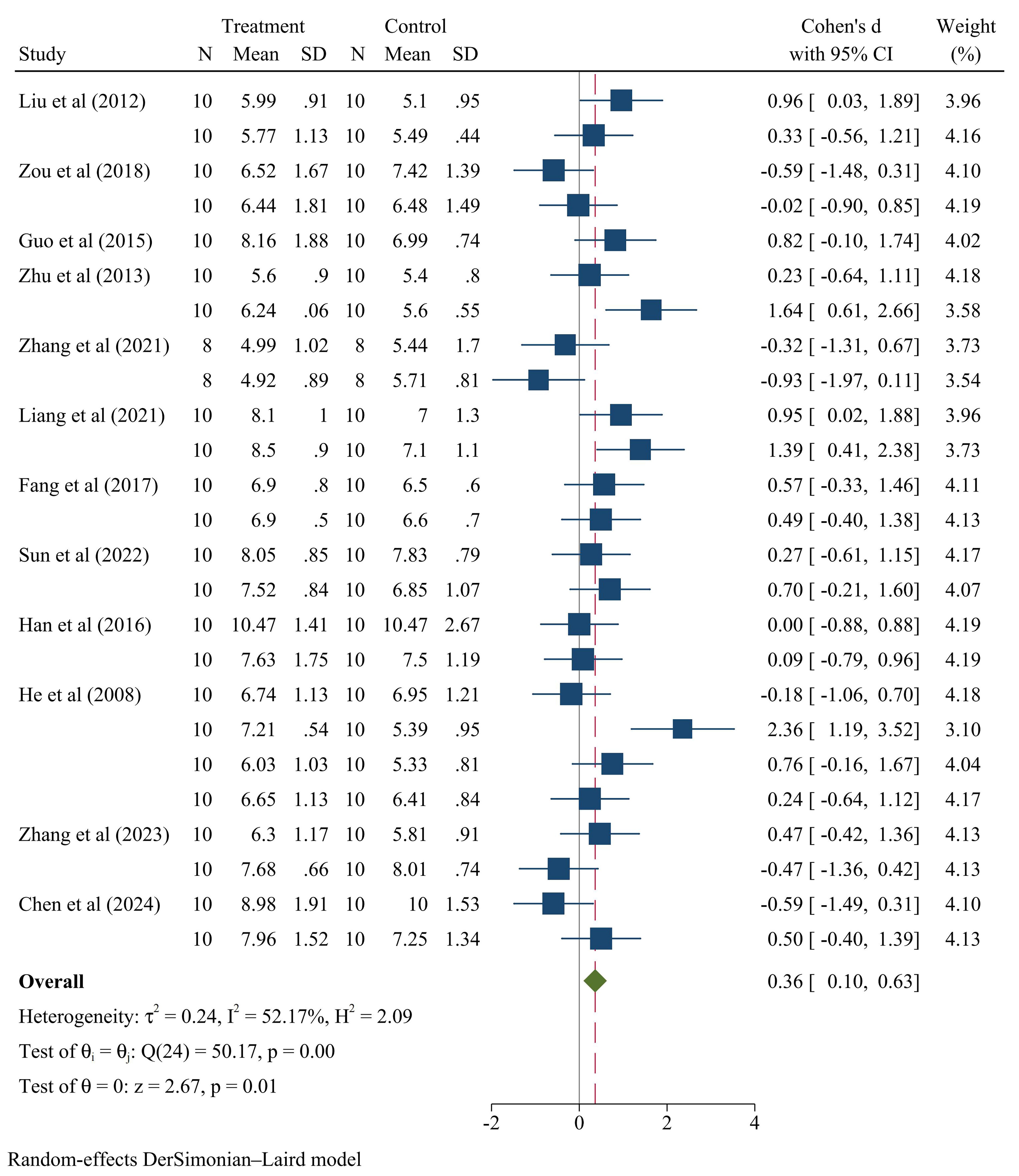


**Figure S38** Consuming high dose of non-nutritional GM maize showed no statistically significant impact on mammalian GLU concentration


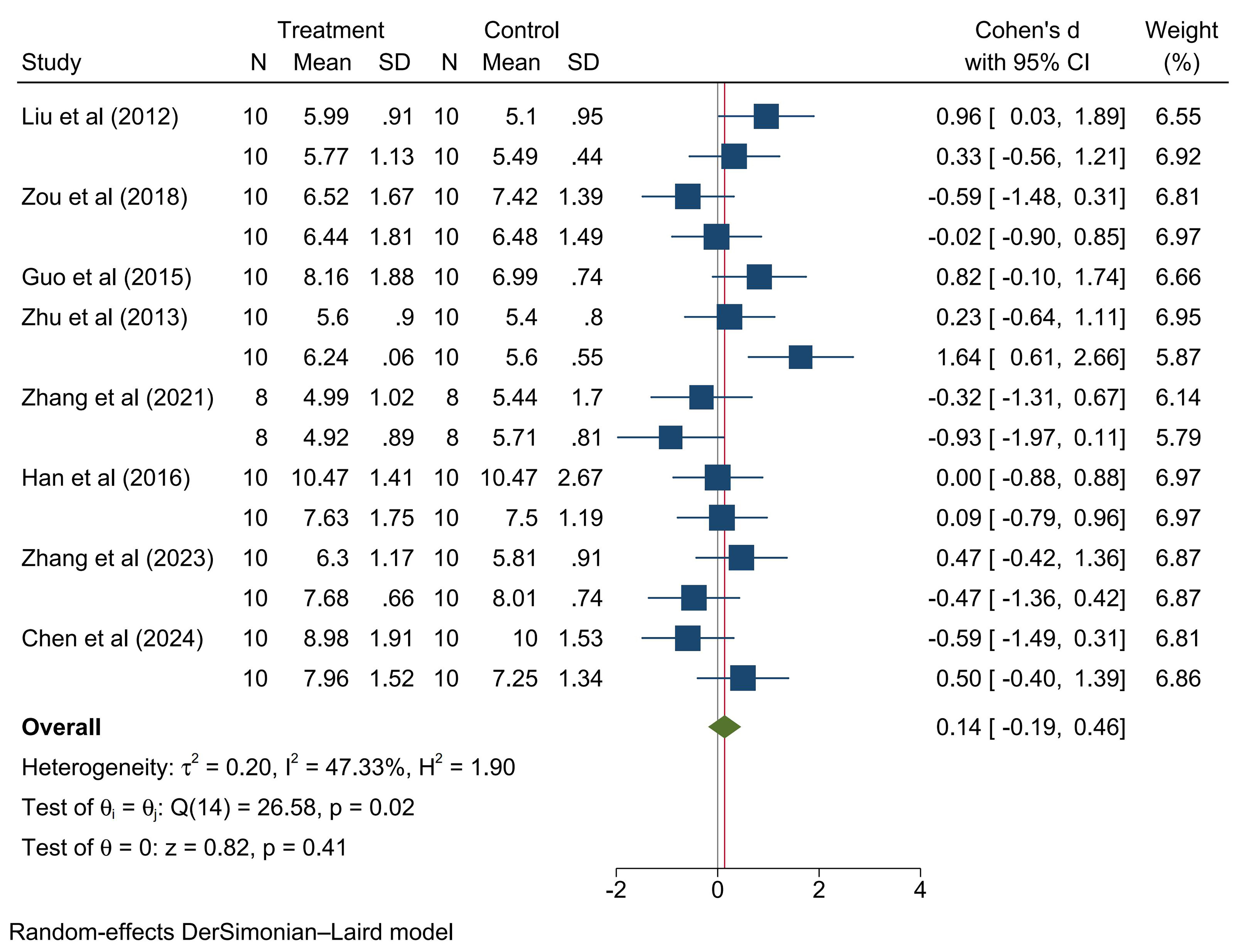

Supplement: Supplementary Figure S32 to S38.docx [file KGMC_A_2603726_SM6469.docx]
