# Supplementary material for: Comprehensive risk assessment revealed some physiological indicators responding to various GM-crop consumption
Source: GM Crops Food. 2025 Dec 19;17(1):2603726. doi: 10.1080/21645698.2025.2603726 (PMC12721096; doi:10.1080/21645698.2025.2603726)
Supplement: Supplementary Figure S45 to S49.docx [file KGMC_A_2603726_SM6468.docx]

**Electrolyte concentrations after GM-maize consumption**

**Figure S45** Consuming GM maize showed no statistically significant impact on mammalian serum K^+^ concentration.


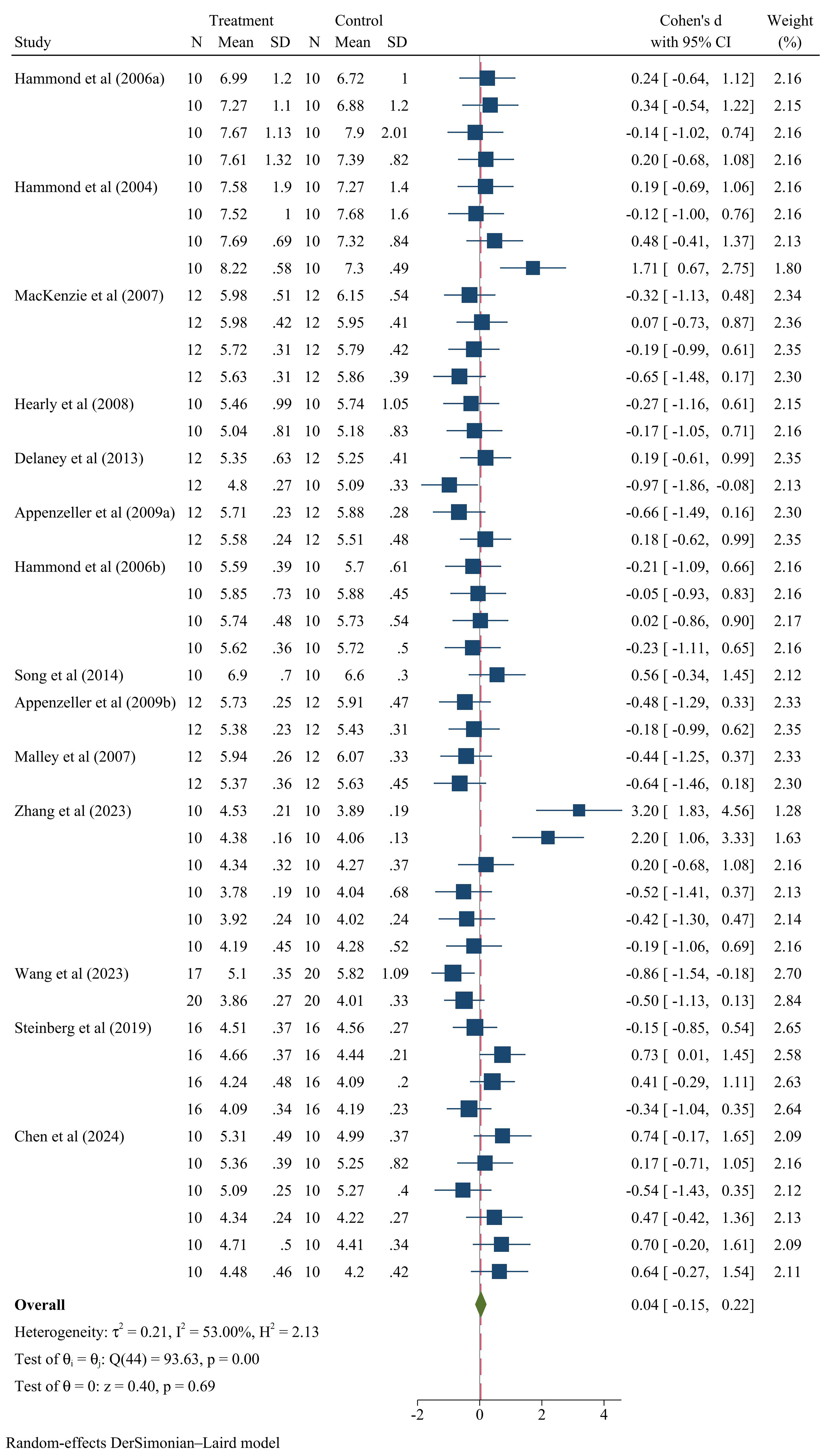


**Figure S46** Consuming GM maize showed no statistically significant impact on mammalian serum Ca^2+^ concentration.


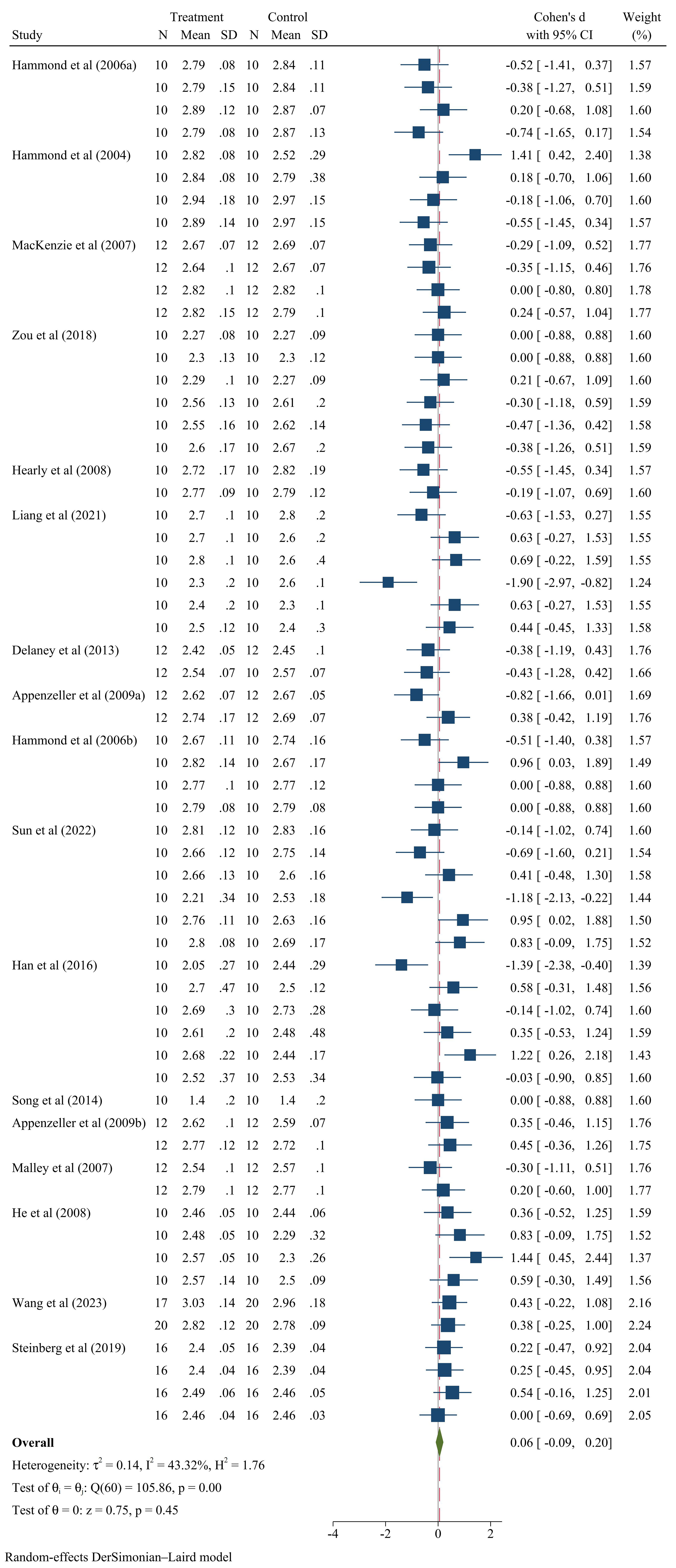


**Figure S47** Consuming GM maize showed no statistically significant impact on mammalian serum P concentration.


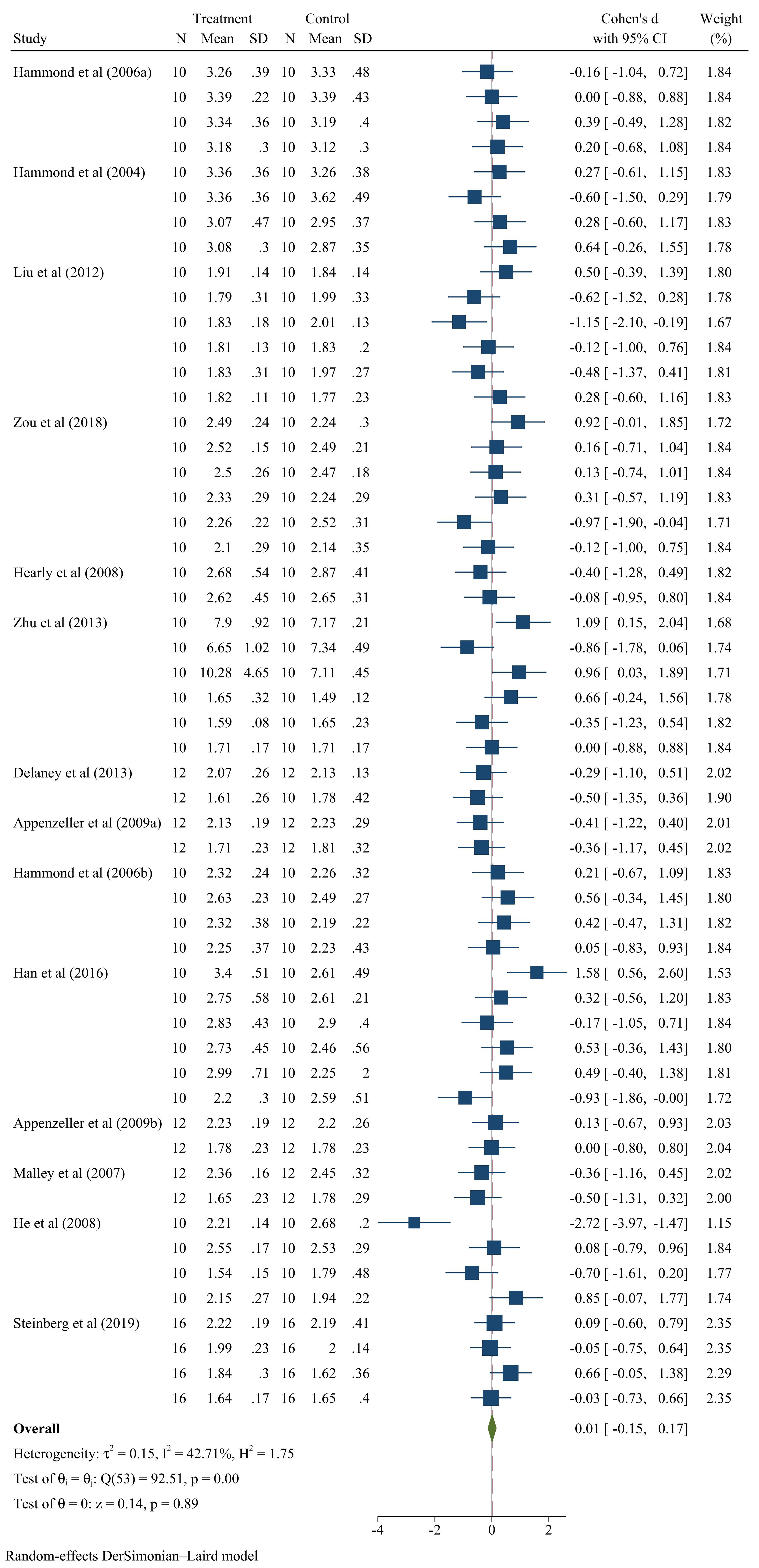


**Figure S48** Consuming GM maize showed no statistically significant impact on mammalian serum Na^+^ concentration.


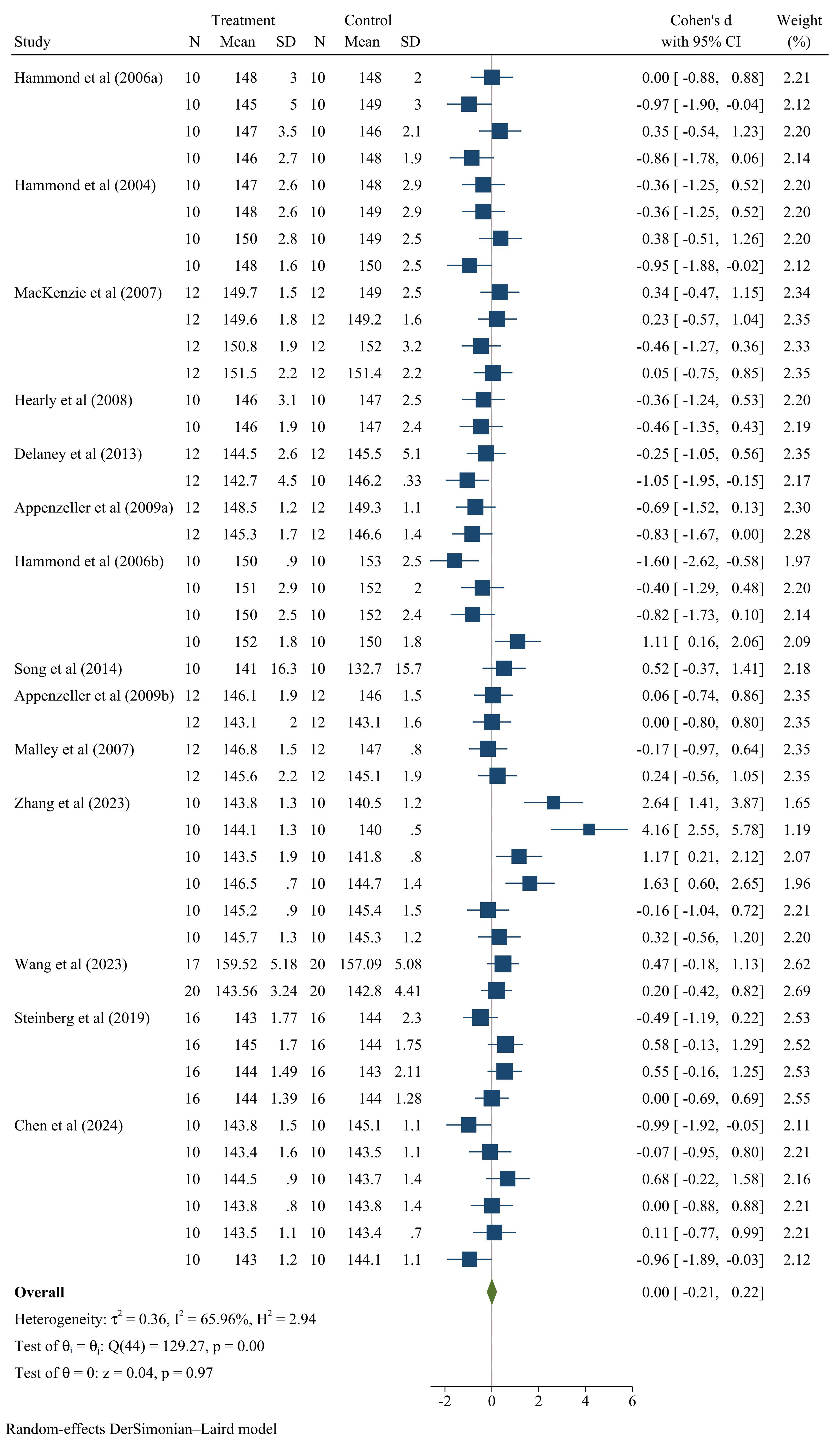


**Figure S49** Consuming GM maize showed no statistically significant impact on mammalian serum Cl concentration.

**
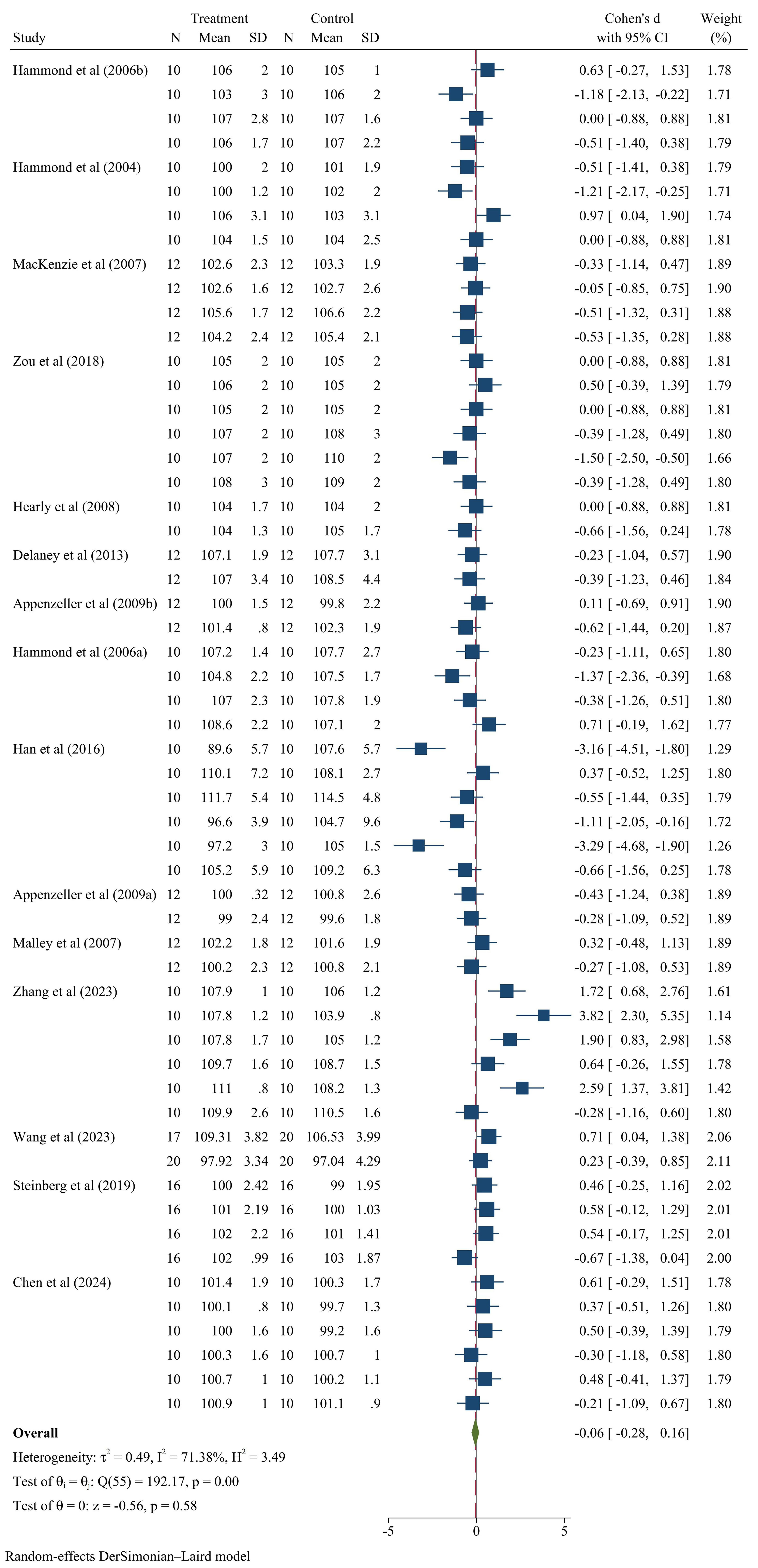
**
