# Supplementary material for: Comprehensive risk assessment revealed some physiological indicators responding to various GM-crop consumption
Source: GM Crops Food. 2025 Dec 19;17(1):2603726. doi: 10.1080/21645698.2025.2603726 (PMC12721096; doi:10.1080/21645698.2025.2603726)
Supplement: Supplementary Figure S12 to S25.docx [file KGMC_A_2603726_SM6466.docx]

**Relative organ weight after GM-rice consumption**

**Figure S12** Consuming GM rice showed no statistically significant impact on mammalian relative brain weight


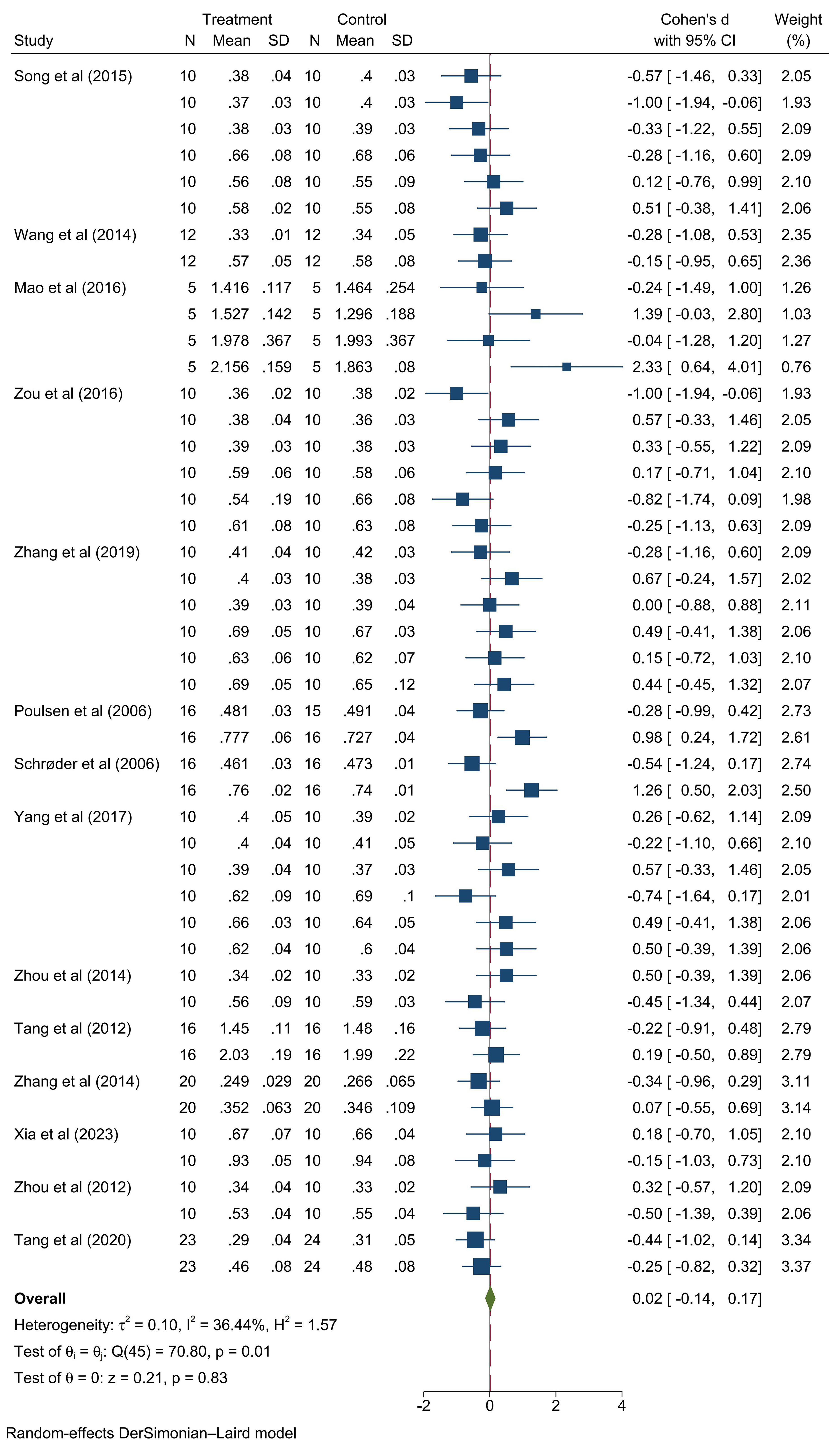


**Figure S13** Consuming GM rice showed no statistically significant impact on mammalian relative heart weight


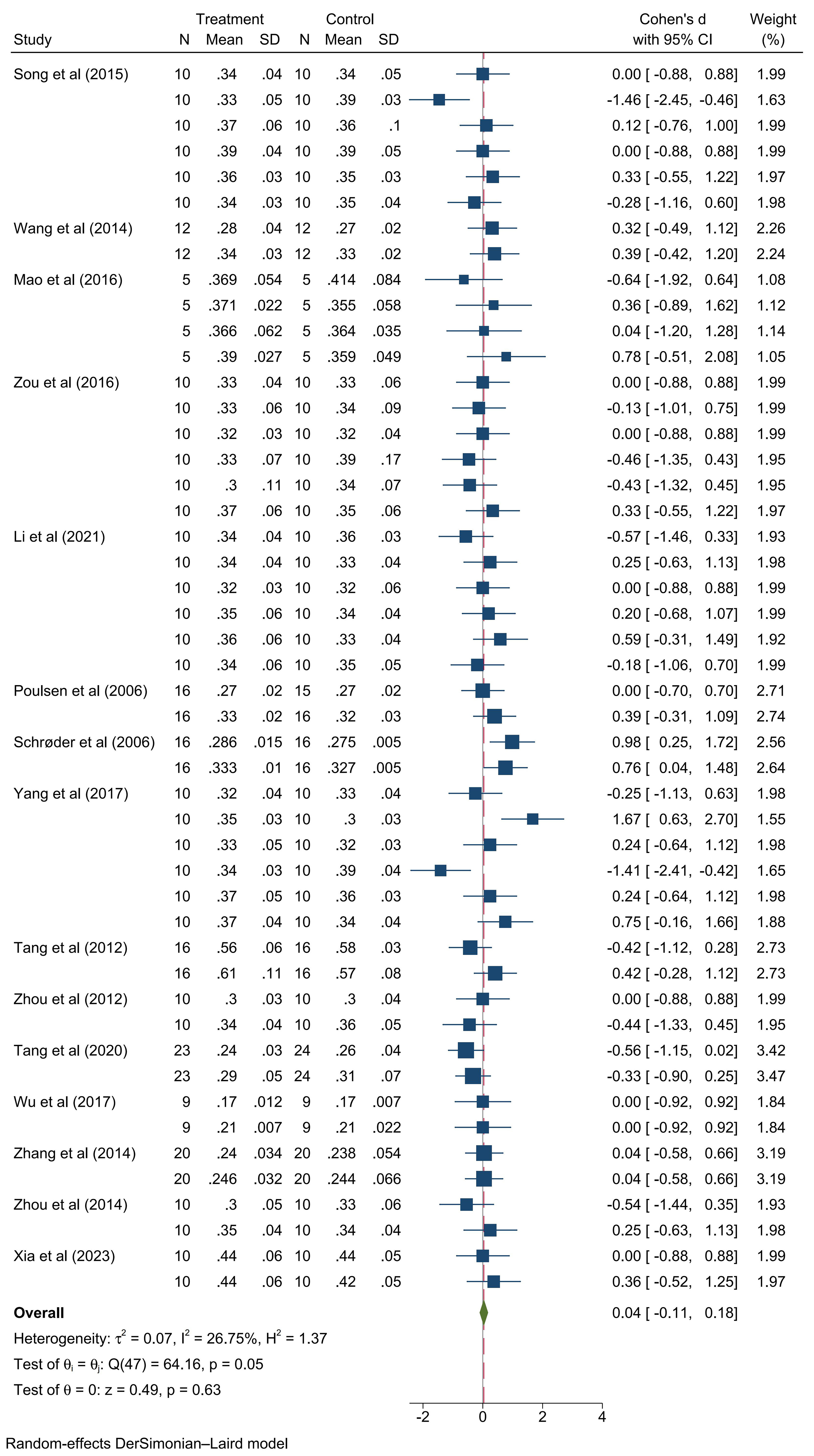


**Figure S14** Consuming GM rice showed no statistically significant impact on mammalian relative liver weight


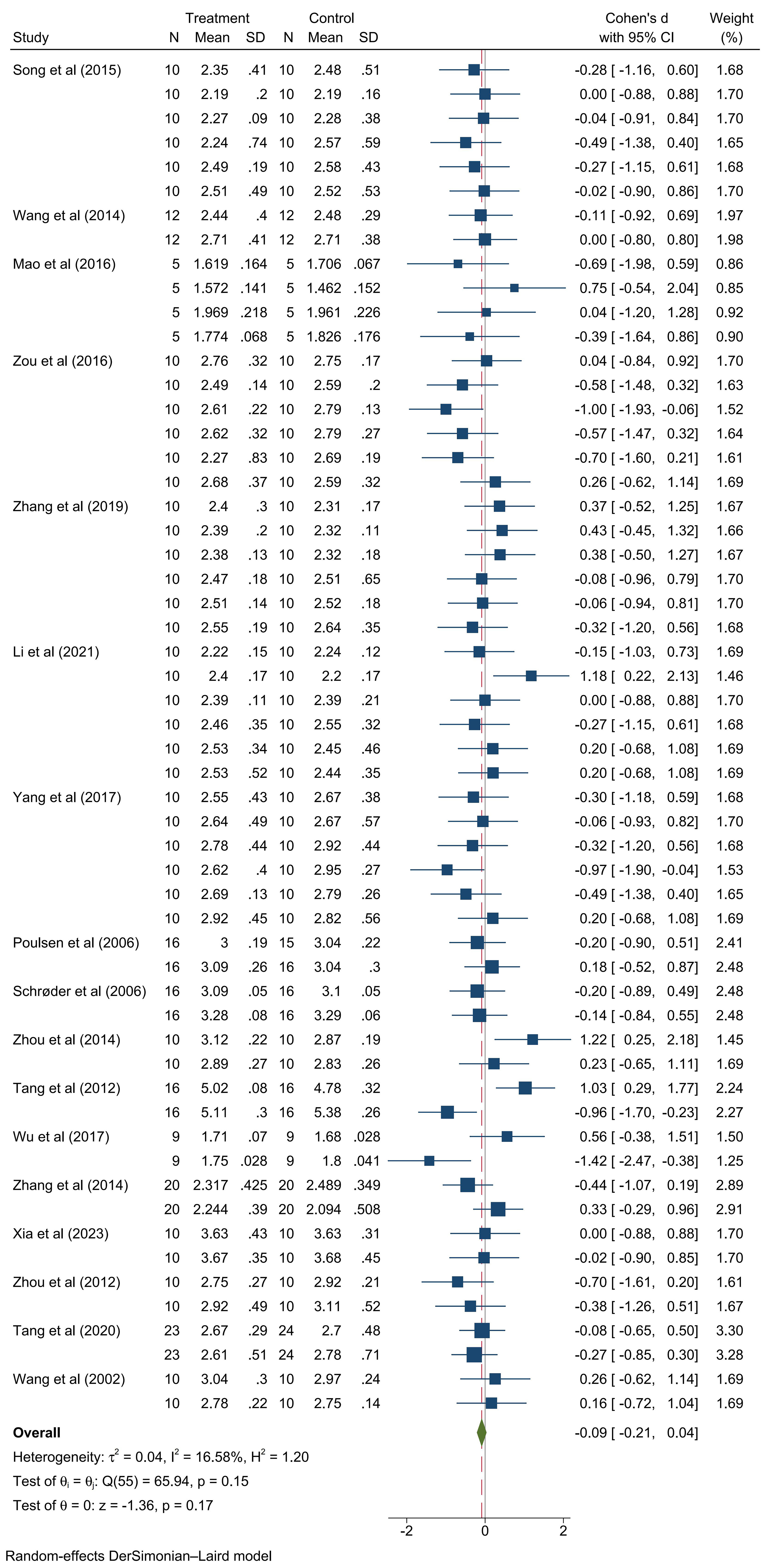


**Figure S15** Consuming GM rice showed no statistically significant impact on mammalian relative spleen weight


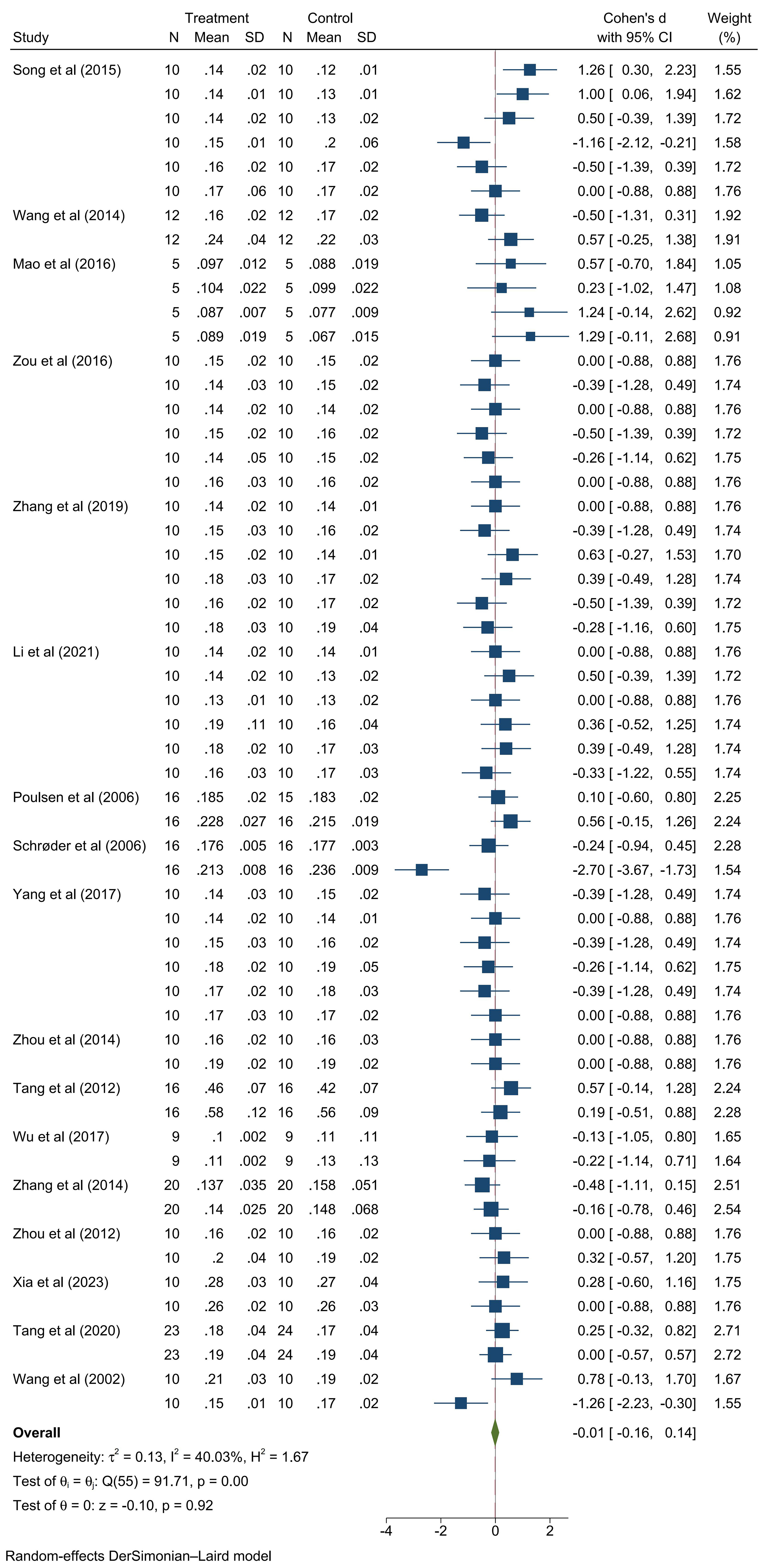


**Figure S16** Consuming GM rice led to statistically significant increase on mammalian relative kidney weight


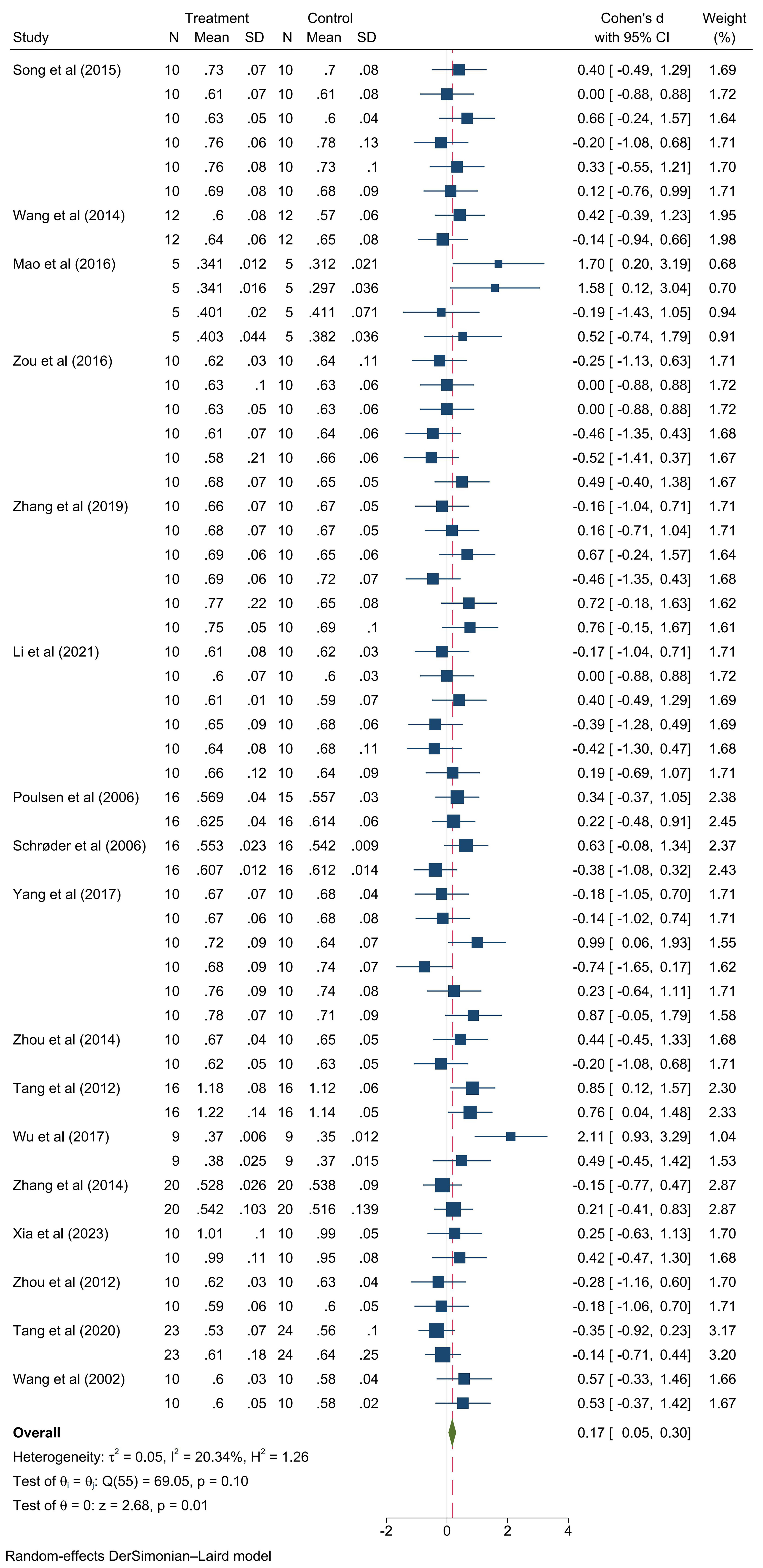


**Figure S17** Consuming low dose of GM rice showed no statistically significant impact on mammalian relative kidney weight


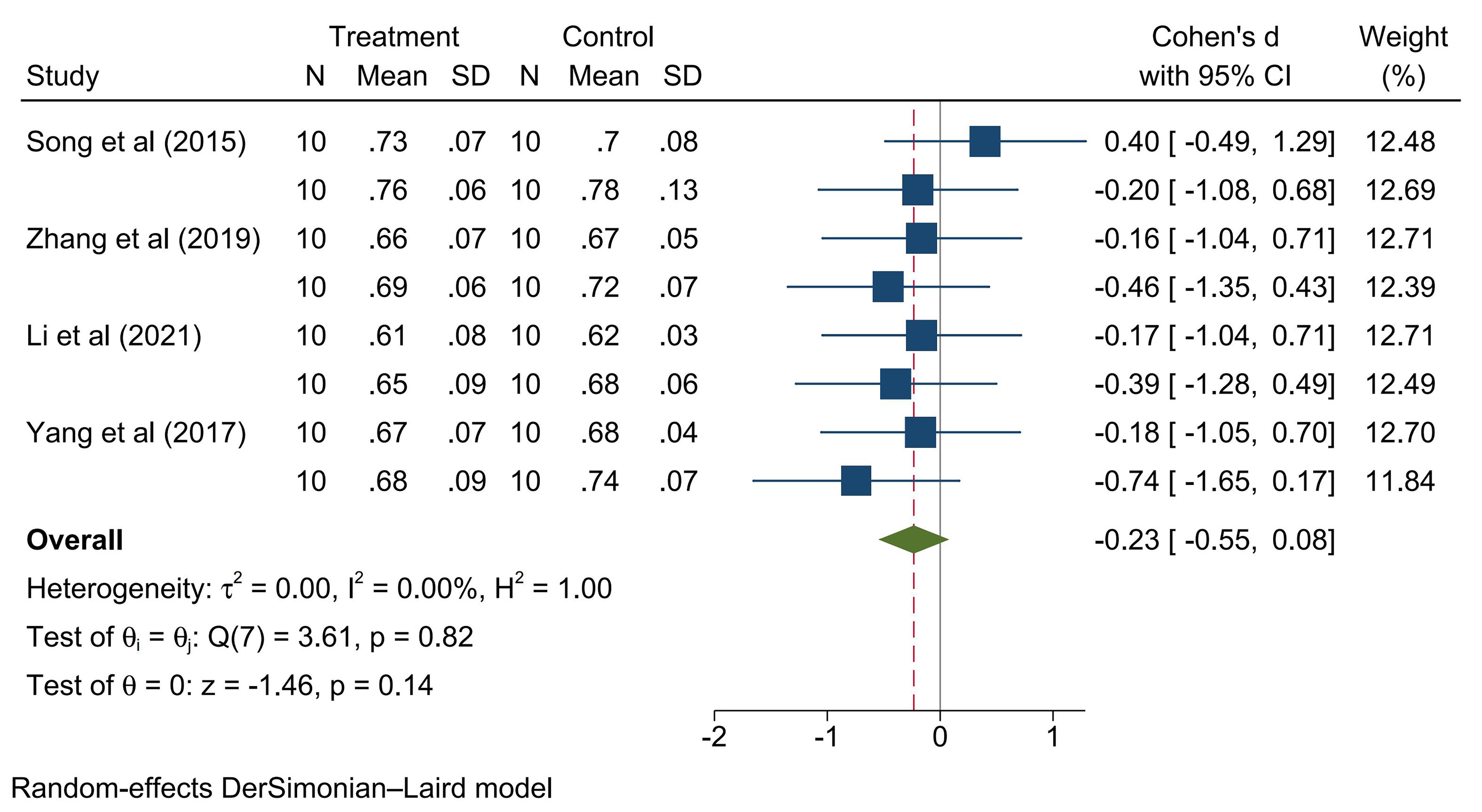


**Figure S18** Consuming medium dose of GM rice led to statistically significant increase on mammalian relative kidney weight


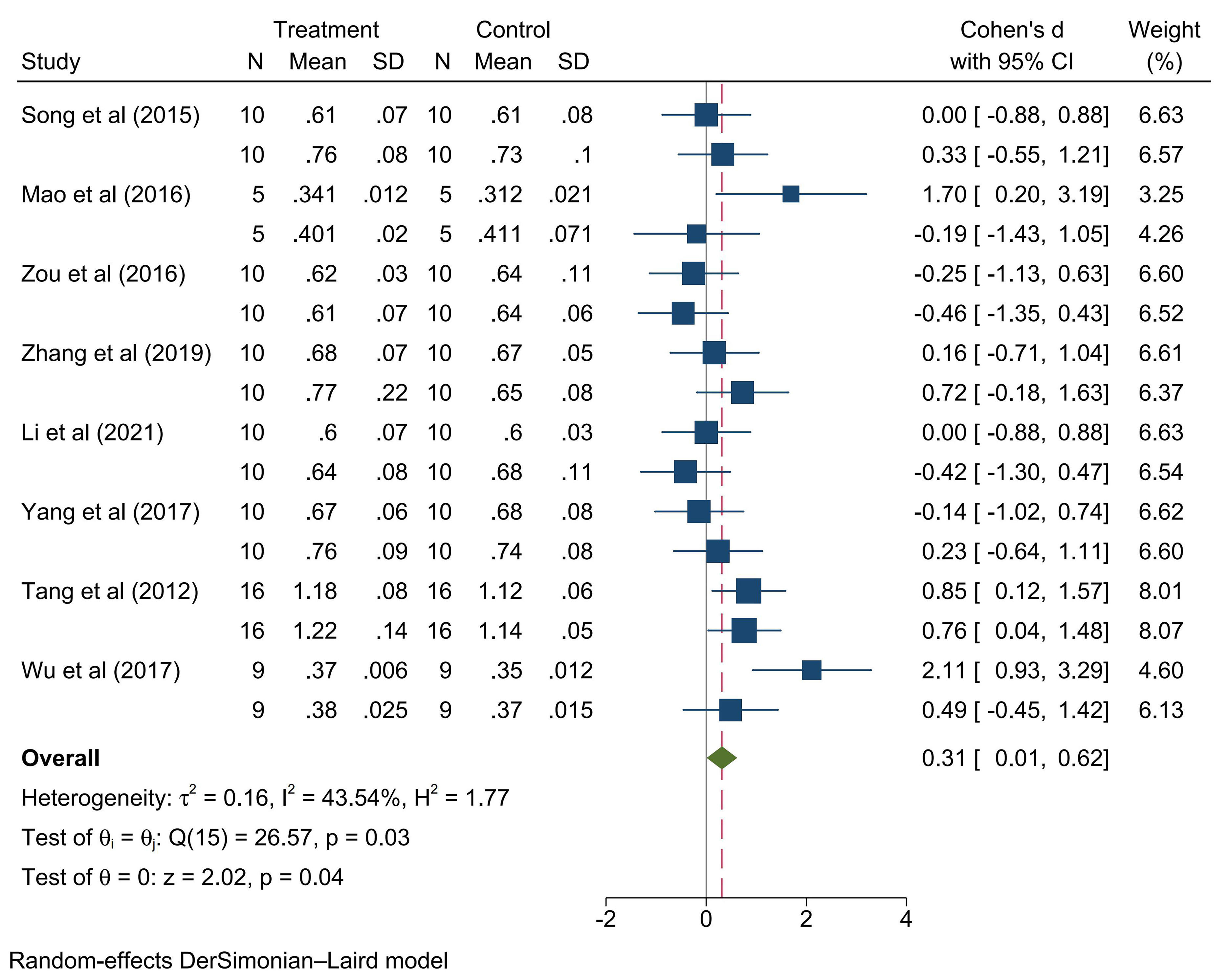


**Figure S19** Consuming high dose of GM rice led to statistically significant increase on mammalian relative kidney weight


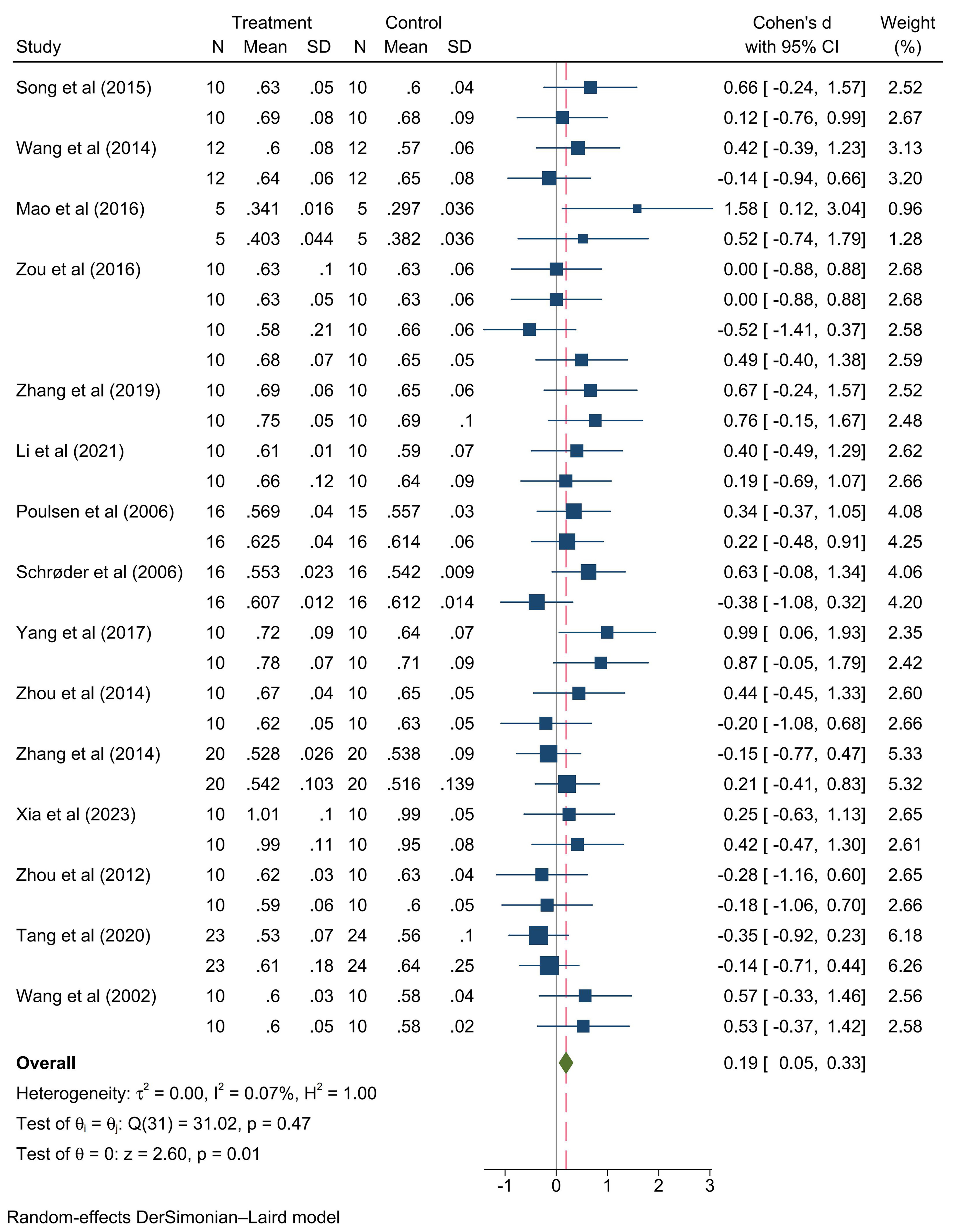


**Figure S20** Consuming medium dose of non-nutritional GM rice showed no statistically significant impact on mammalian relative kidney weight

**
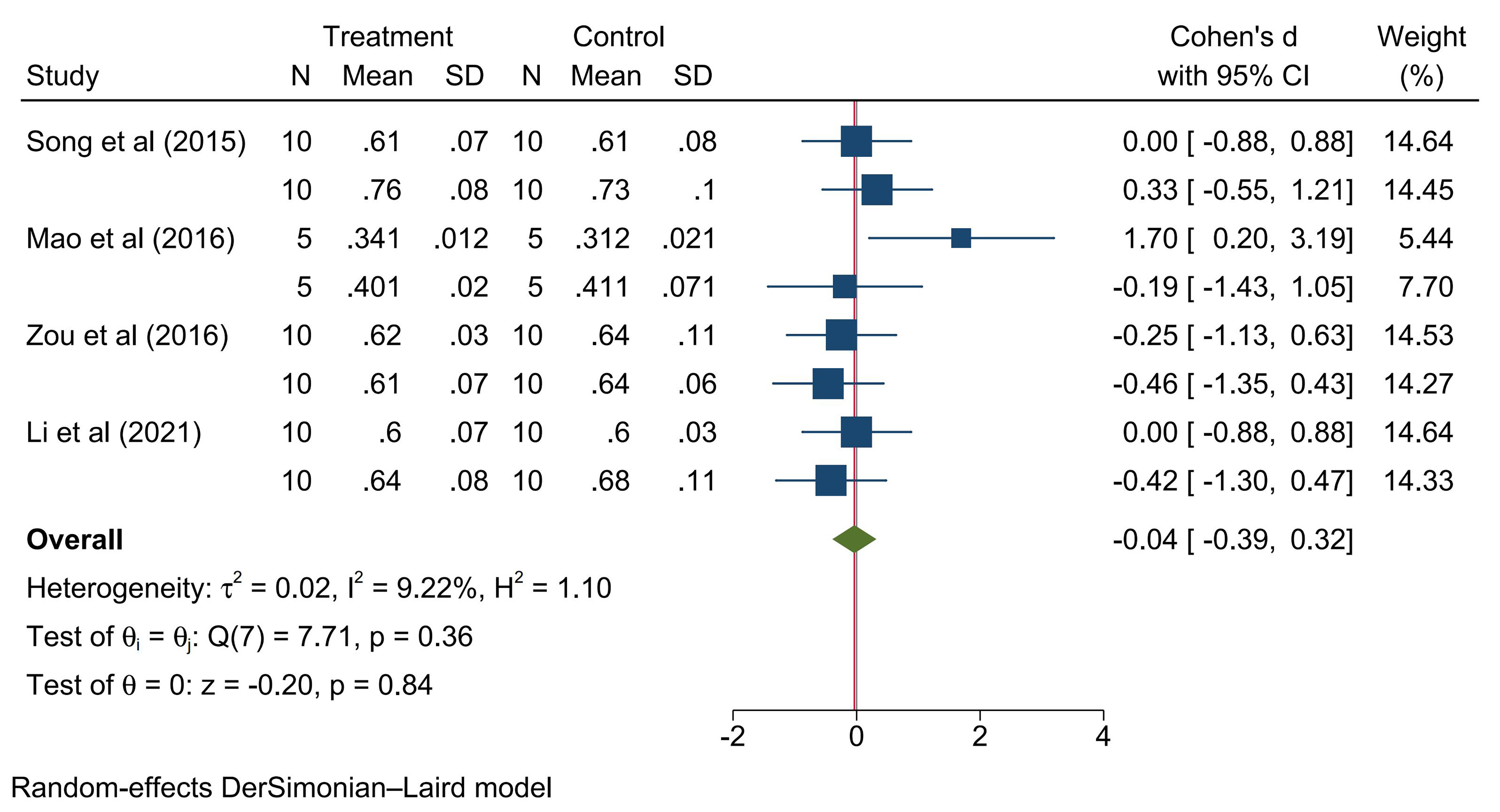
**

**Figure S21** Consuming high dose of non-nutritional GM rice showed no statistically significant impact on mammalian relative kidney weight

**
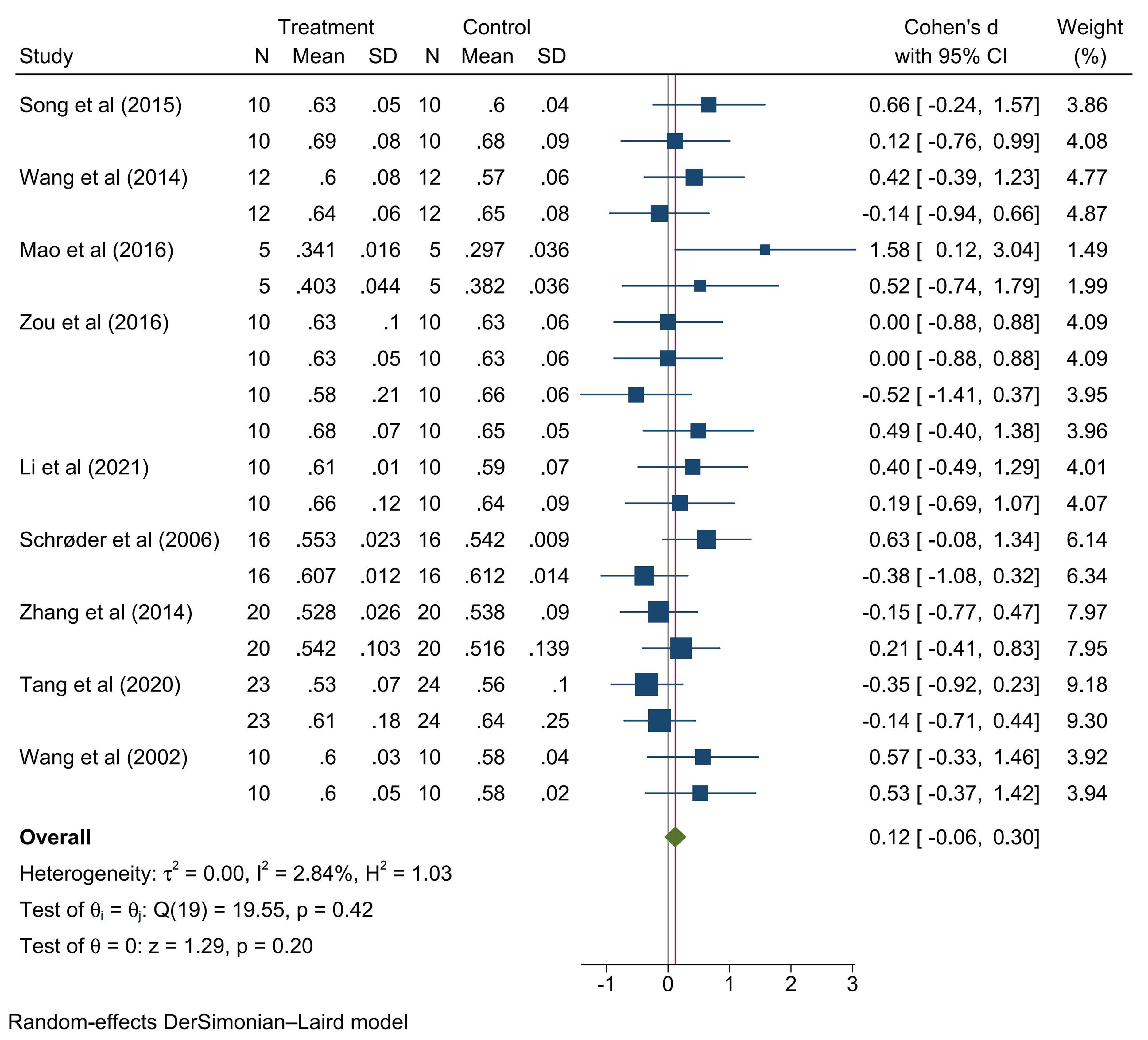
**

**Figure S22** Consuming GM rice led to statistically significant decrease on mammalian relative lung weight


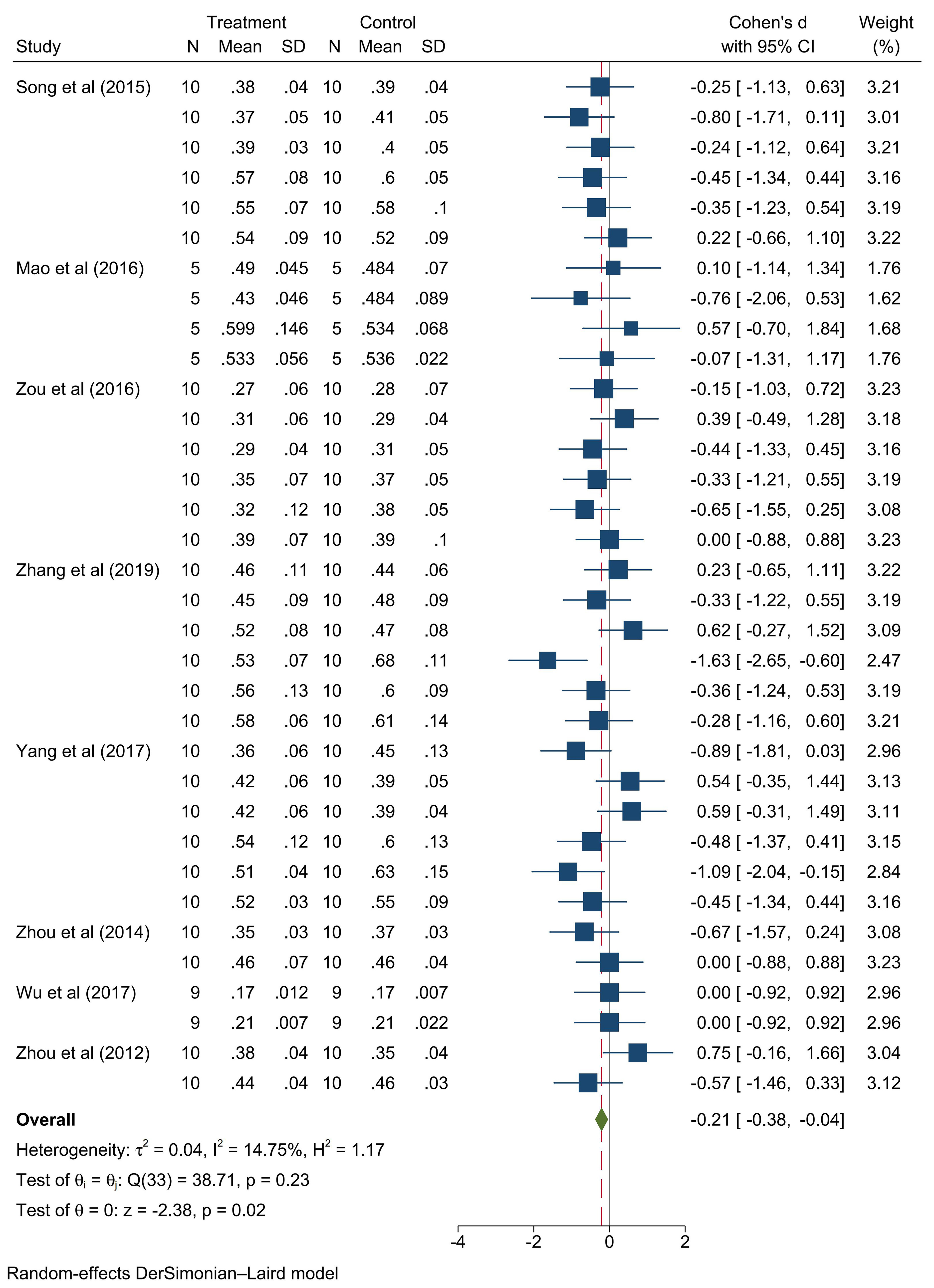


**Figure S23** Consuming low dose of GM rice led to statistically significant decrease on mammalian relative lung weight


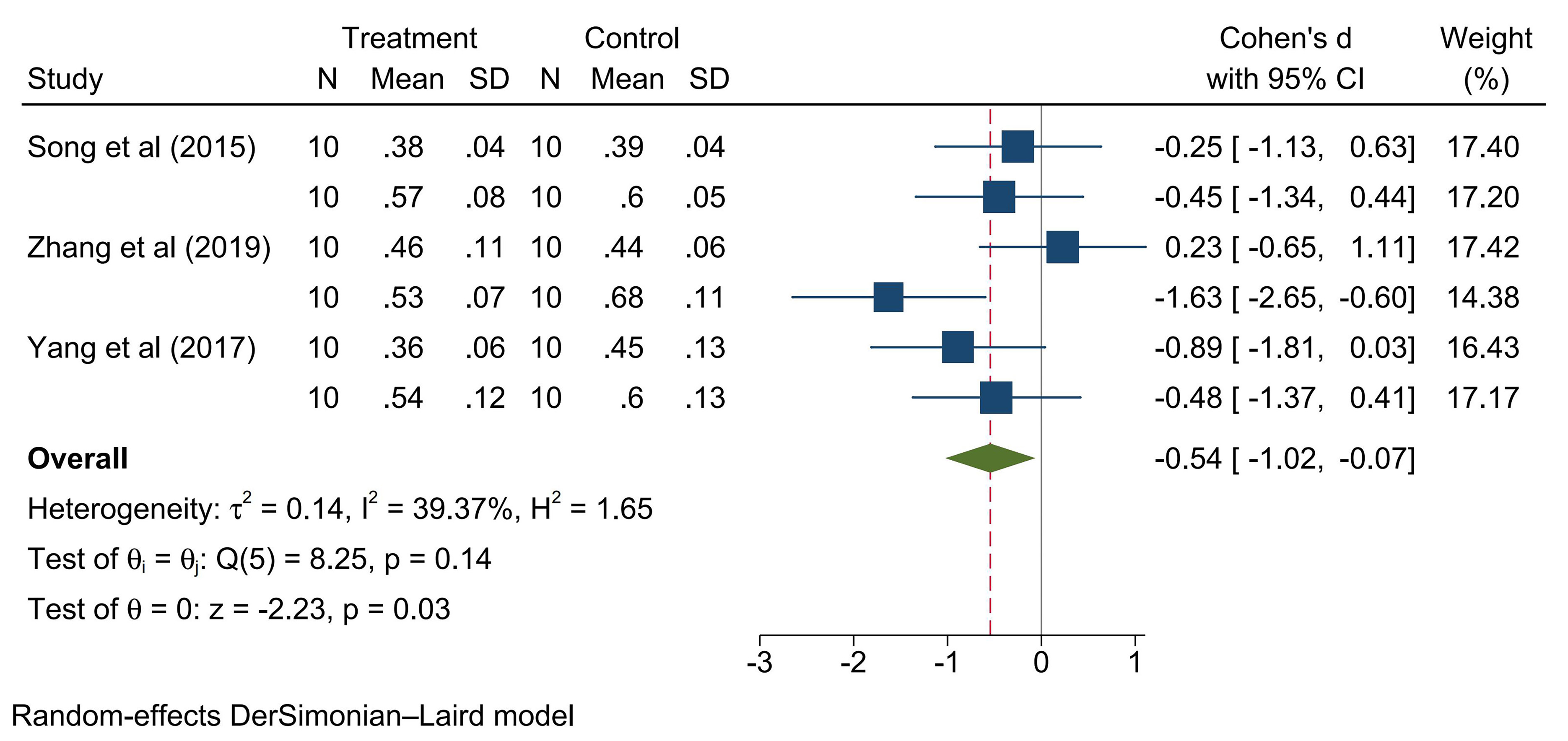


**Figure S24** Consuming medium dose of GM rice showed no statistically significant impact on mammalian relative lung weight


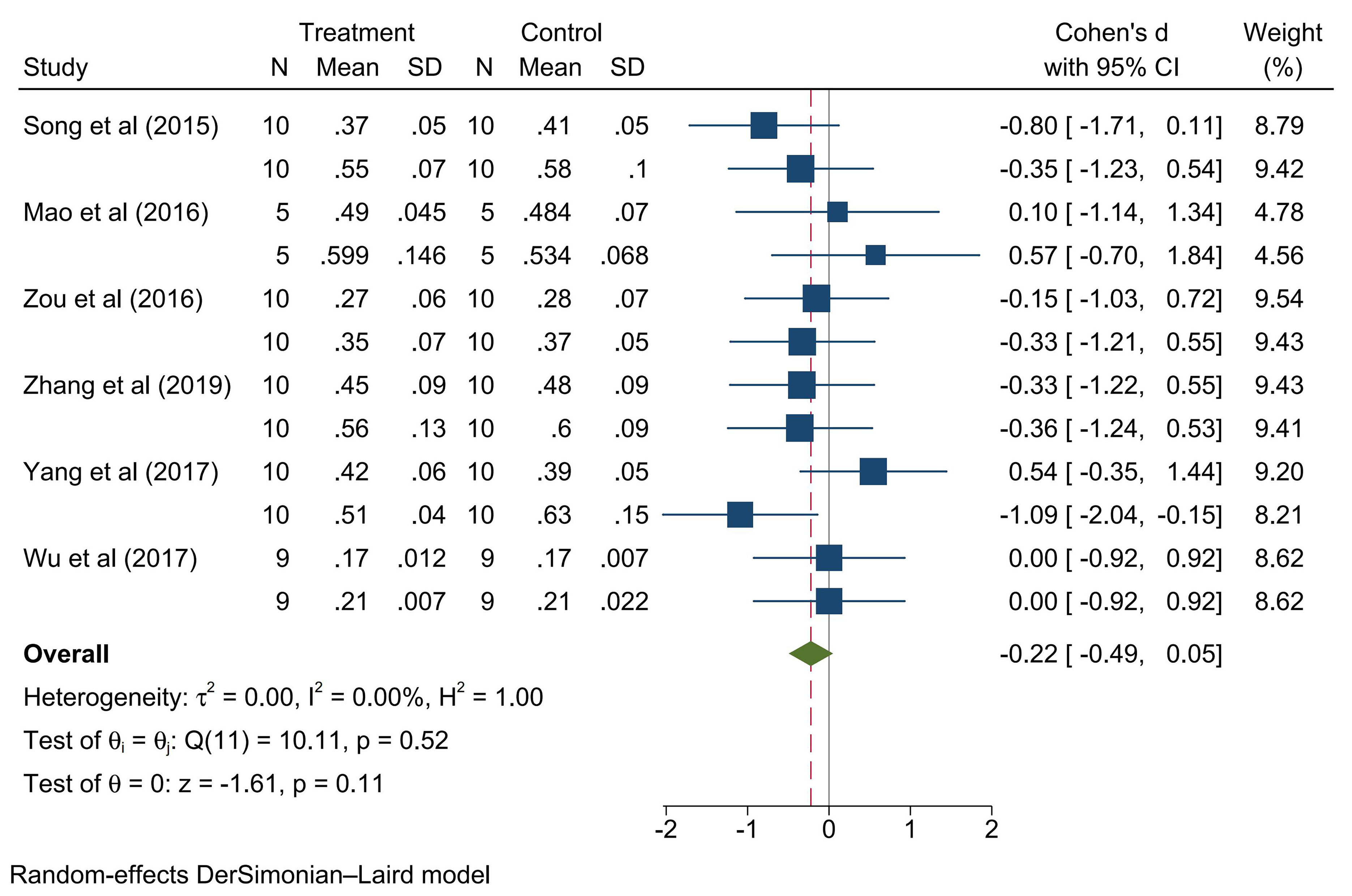


**Figure S25** Consuming high dose of GM rice showed no statistically significant impact on mammalian relative lung weight

**
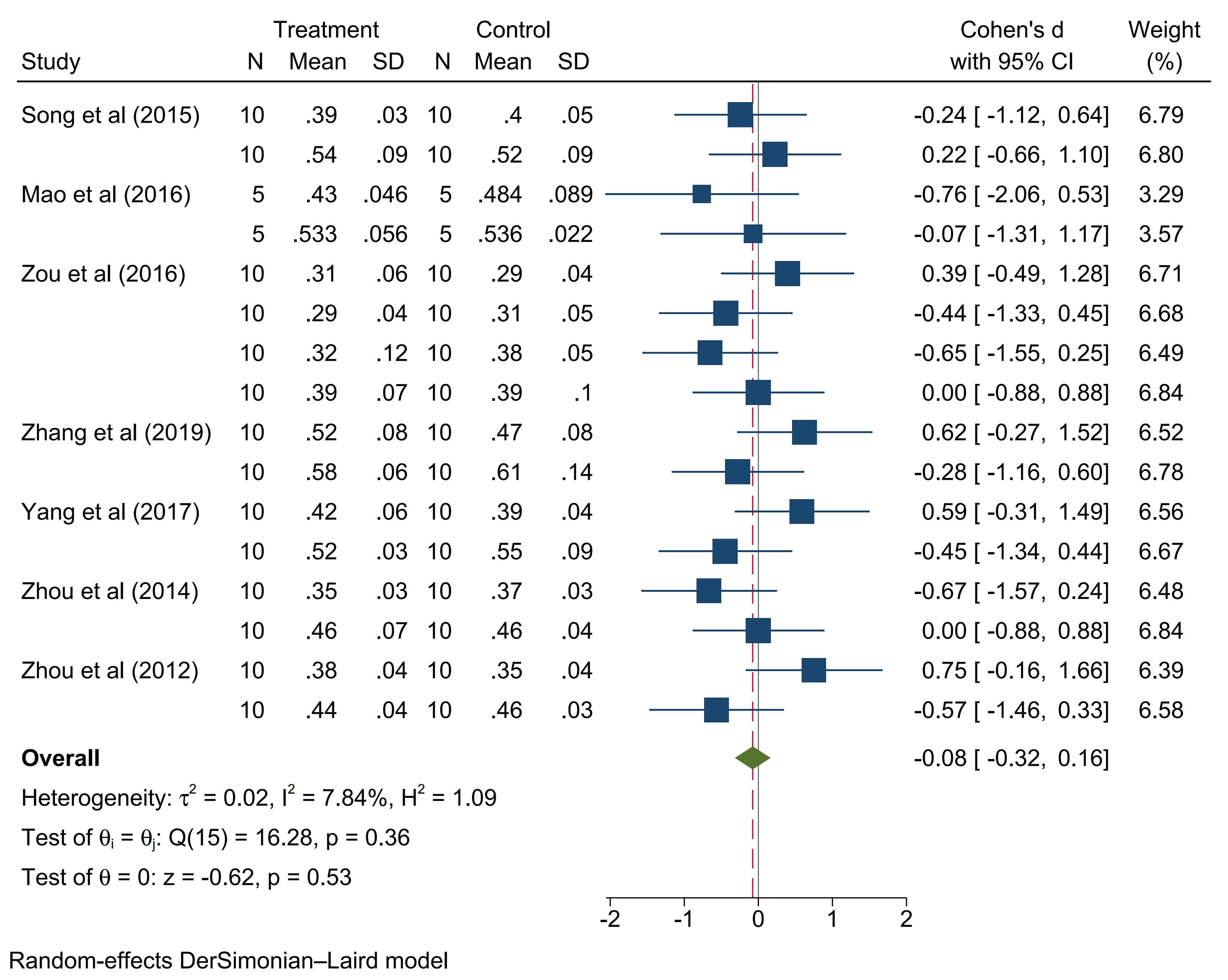
**
