# Supplementary material for: Comprehensive risk assessment revealed some physiological indicators responding to various GM-crop consumption
Source: GM Crops Food. 2025 Dec 19;17(1):2603726. doi: 10.1080/21645698.2025.2603726 (PMC12721096; doi:10.1080/21645698.2025.2603726)
Supplement: Supplementary Tables.docx [file KGMC_A_2603726_SM6465.docx]

**Table-S1** Consumption of GM maize showed no statistically significant impact on mammalian weight gain.

1. Comparison of body weight gain between GM-maize group and non-GM maize group

| **Crops** | **Study** | **Ages (weeks)** | **ΔGM-mean** | **GM-N** | **ΔCK-mean** | **CK-N** | **Sex** | **Dosage (%)** | **Kinds of Animals** |
| --- | --- | --- | --- | --- | --- | --- | --- | --- | --- |
| maize | Zhang et al (2022) | 9-18 | 186.9 | 10 | 176.3 | 10 | male | 33 | Rats |
| maize |  | 9-18 | 194.5 | 10 | 171.8 | 10 | male | 66 | Rats |
| maize |  | 9-18 | 64.9 | 20 | 42.6 | 20 | female | 33 | Rats |
| maize |  | 9-18 | 50 | 20 | 45.94 | 20 | female | 66 | Rats |
| maize |  | 4-17 | 186.4 | 10 | 186.9 | 10 | male | 33 | Rats |
| maize |  | 4-17 | 197.9 | 10 | 183 | 10 | male | 66 | Rats |
| maize |  | 4-17 | 142.2 | 20 | 145.2 | 20 | female | 33 | Rats |
| maize |  | 4-17 | 149.2 | 20 | 149.8 | 20 | female | 66 | Rats |
| maize |  | 4-17 | 261.2 | 10 | 255.5 | 10 | male | 33 | Rats |
| maize |  | 4-17 | 282.04 | 10 | 272.8 | 10 | male | 66 | Rats |
| maize |  | 4-17 | 164.84 | 20 | 193.3 | 20 | female | 33 | Rats |
| maize |  | 4-17 | 215 | 20 | 189.64 | 20 | female | 66 | Rats |
| maize | Smith et al (2021) | 7-19 | 351.9 | 16 | 363.9 | 16 | male | 50 | Rats |
| maize |  | 7-19 | 128.2 | 16 | 142.4 | 16 | female | 50 | Rats |
| maize | Carlson et al (2020) | 7-19 | 353.3 | 16 | 324.2 | 16 | male | 50 | Rats |
| maize |  | 7-19 | 122.9 | 15 | 123 | 16 | female | 50 | Rats |
| maize | Wang et al (2023) | 4-13 | 434.8 | 20 | 449.87 | 20 | male | 60 | Rats |
| maize |  | 4-13 | 233.11 | 20 | 232.78 | 20 | female | 60 | Rats |
| maize | Chen et al (2024) | 4-17 | 573.1 | 10 | 566.1 | 10 | male | 17.5 | Rats |
| maize |  | 4-17 | 603.2 | 10 | 563.8 | 10 | male | 35 | Rats |
| maize |  | 4-17 | 634.5 | 10 | 595 | 10 | male | 70 | Rats |
| maize |  | 4-17 | 276.9 | 10 | 288.2 | 10 | female | 17.5 | Rats |
| maize |  | 4-17 | 280.2 | 10 | 281.4 | 10 | female | 35 | Rats |
| maize |  | 4-17 | 288.8 | 10 | 261.9 | 10 | female | 70 | Rats |

ΔGM mean, the average weight gain after consuming GM maize; GM-N, the number of mammals in GM-maize group; ΔCK-mean, the average weight gain after consuming non-GM maize; CK-N, the number of mammals in non-GM group; dosage, percentage of GM maize in feedstock.

**Table-S1** Consumption of GM maize showed no statistically significant impact on mammalian weight gain (continued)

1. Weight gain data for mammals fed GM maize and mammals non-GM maize fits normal distribution test

| **Variable** | **Obs** | **Pr(skewness)** | **Pr(kurtosis)** | **—Joint teat—** | |
| --- | --- | --- | --- | --- | --- |
|  |  |  |  | Adj chi2(2) | Prob>chi2 |
| ΔGM mean | 24 | 0.0236 | 0.3514 | 5.67 | 0.0587 **^#^** |
| ΔCK mean | 24 | 0.0394 | 0.5051 | 4.72 | 0.0944 **^#^** |

Obs, objectives; Adj chi2(2), the p value of the adjusted Chi-square test; Pr(skewness), probability of skewness; Pr(kurtosis), probability of kurtosis; **#**: p>0.05, weight gain data for mammals fed GM maize and mammals fed non-GM maize fits a normal distribution.

1. Consumption of GM maize showed no statistically significant impact on mammalian weight gain.

| **Variable** | **Obs** | **Mean** | **Std. err.** | **Std. dev.** | **[95 % conf. interval]** | |
| --- | --- | --- | --- | --- | --- | --- |
| ΔGM mean | 24 | 265.6662 | 32.42073 | 158.8285 | 198.5989 | 332.7336 |
| ΔCK mean | 24 | 258.5554 | 31.21278 | 152.9108 | 193.9869 | 323.124 |
| Combined | 48 | 262.1108 | 22.26727 | 154.2721 | 217.3149 | 306.9068 |
| diff. |  | 7.110833 | 45.00379 |  | -83.37886 | 97.60053 |

Obs, objectives; diff, mean (Δ GM mean)-mean (Δ CK-mean); Std. err., standard error Welch`s degrees of freedom = 47.9281; Std. dev., standard Deviation; t = 0.1580, consumption of GM maize showed no statistically significant impact on mammalian weight gain.

**Table-S2** Consumption of GM rice showed no statistically significant impact on mammalian weight gain.

1. Comparison of body weight gain between GM rice intake mammals and non-GM rice intake mammals

| **Crops** | **Study** | **Ages (weeks)** | **ΔGM-mean** | **GM-N** | **ΔCK-mean** | **CK-N** | **Sex** | **Dosage (%)** | **Kinds of Animals** |
| --- | --- | --- | --- | --- | --- | --- | --- | --- | --- |
| rice | Li et al (2021) | 4-16 | 228.9 | 10 | 232.2 | 10 | female | 15 | Rats |
| rice |  | 4-16 | 234.8 | 10 | 215.1 | 10 | female | 30 | Rats |
| rice |  | 4-16 | 227.5 | 10 | 228.4 | 10 | female | 60 | Rats |
| rice |  | 4-16 | 451.6 | 10 | 479.5 | 10 | male | 15 | Rats |
| rice |  | 4-16 | 472.1 | 10 | 464.4 | 10 | male | 30 | Rats |
| rice |  | 4-16 | 458.4 | 10 | 479.4 | 10 | male | 60 | Rats |
| rice | Yang et al (2017) | 5-17 | 405 | 10 | 406 | 10 | male | 17.5 | Rats |
| rice |  | 5-17 | 407 | 10 | 394 | 10 | male | 35 | Rats |
| rice |  | 5-17 | 415 | 10 | 448 | 10 | male | 70 | Rats |
| rice |  | 5-17 | 188 | 10 | 165 | 10 | female | 17.5 | Rats |
| rice |  | 5-17 | 170 | 10 | 178 | 10 | female | 35 | Rats |
| rice |  | 5-17 | 194 | 10 | 198 | 10 | female | 70 | Rats |
| rice |  | 5-17 | 447.2 | 10 | 445.2 | 10 | male | 17.5 | Rats |
| rice |  | 5-17 | 465.4 | 10 | 440.8 | 10 | male | 35 | Rats |
| rice |  | 5-17 | 446.3 | 10 | 469 | 10 | male | 70 | Rats |
| rice |  | 5-17 | 197.5 | 10 | 201.6 | 10 | female | 17.5 | Rats |
| rice |  | 5-17 | 230.1 | 10 | 234 | 10 | female | 35 | Rats |
| rice |  | 5-17 | 205.7 | 10 | 197.3 | 10 | female | 70 | Rats |
| rice |  | 5-17 | 339.4 | 10 | 353.4 | 10 | male | 64 | Rats |
| rice |  | 5-17 | 189.1 | 10 | 196.4 | 10 | female | 64 | Rats |
| rice | MOMMA et al (2020) | 4-11 | 176 | 7 | 175 | 7 | male | 25 | Rats |

ΔGM mean, the average weight gain after consuming GM rice; GM-N, the number of mammals in GM-rice group; ΔCK-mean, the average weight gain after consuming non-GM rice; CK-N, the number of mammals in non-GM rice group; dosage, percentage of GM rice in feedstock.

**Table-S2** Consumption of GM rice showed no statistically significant impact on mammalian weight gain (continued)

1. Weight gain data for mammals fed GM rice and mammals non-GM maize does not fit normal distribution test

| **Variable** | **Obs** | **Pr(skewness)** | **Pr(kurtosis)** | **—Joint teat—** | |
| --- | --- | --- | --- | --- | --- |
|  |  |  |  | Adj chi2(2) | Prob>chi2 |
| ΔGM mean | 21 | 0.7027 | 0.0000 | 17.03 | 0.0002 * |
| ΔCK mean | 21 | 0.6942 | 0.0000 | 16.44 | 0.0003 * |

Obs, objectives; Adj chi2(2), the p value of the adjusted Chi-square test; Pr(skewness), probability of skewness; Pr(kurtosis), probability of kurtosis; *: p<0.05, weight gain data for mammals fed GM rice and mammals fed non-GM rice does not fit a normal distribution.

1. Consumption of GM rice showed no statistically significant impact on mammalian weight gain.

| **Group** | **Obs** | **Rank sum** | **Expected** |
| --- | --- | --- | --- |
| ΔGM mean | 21 | 447 | 451.5 |
| ΔCK mean | 21 | 456 | 451.5 |
| combined | 42 | 903 | 903 |

Adjusted variance: 1580.25; rank sum, Mann-Whitney U test. z = -0.113; p = 0.9207. Consumption of GM rice showed no statistically significant impact on mammalian weight gain.

**Table-S3** Consumption of GM soybean showed no statistically significant impact on mammalian weight gain.

1. Comparison of body weight gain between GM soybean intake mammals and non-GM soybean intake mammals

| **Crops** | **Study** | **Ages (weeks)** | **ΔGM-mean** | **GM-N** | **ΔCK-mean** | **CK-N** | **Sex** | **Dosage (%)** | **Kinds of Animals** |
| --- | --- | --- | --- | --- | --- | --- | --- | --- | --- |
| soybean | Qian et al (2018) | 10-17 | 453.4 | 10 | 422.5 | 10 | male | 7.5 | Rats |
| soybean |  | 10-17 | 419.8 | 10 | 428.9 | 10 | male | 15 | Rats |
| soybean |  | 10-17 | 415 | 10 | 427.3 | 10 | male | 30 | Rats |
| soybean |  | 10-17 | 161.3 | 10 | 211.1 | 10 | female | 7.5 | Rats |
| soybean |  | 10-17 | 155.7 | 10 | 218.8 | 10 | female | 15 | Rats |
| soybean |  | 10-17 | 162.1 | 10 | 221.6 | 10 | female | 30 | Rats |
| soybean | Yang et al（2017） | 10-17 | 284.8 | 10 | 391.8 | 10 | male | 7.5 | Rats |
| soybean |  | 10-17 | 288 | 10 | 365.6 | 10 | male | 15 | Rats |
| soybean |  | 10-17 | 293 | 10 | 393.9 | 10 | male | 30 | Rats |
| soybean |  | 10-17 | 180.9 | 10 | 191.1 | 10 | female | 7.5 | Rats |
| soybean |  | 10-17 | 180.7 | 10 | 197.9 | 10 | female | 15 | Rats |
| soybean |  | 10-17 | 177.1 | 10 | 196.2 | 10 | female | 30 | Rats |
| soybean |  | 7-18 | 39.6 | 16 | 45.4 | 16 | male | 30 | Rats |
| soybean | MOMMA et al (2020) | 7-18 | 90.5 | 12 | 89.9 | 12 | female | 30 | Rats |

ΔGM mean, the average weight gain after consuming GM soybean; GM-N, the number of mammals in GM-soybean group; ΔCK-mean, the average weight gain after consuming non-GM soybean; CK-N, the number of mammals in non-GM group; dosage, percentage of GM soybean in feedstock.

**Table-S3** Consumption of GM soybean showed no statistically significant impact on mammalian weight gain (continued)

1. Weight gain data for mammals fed GM soybean and mammals non-GM soybean fits normal distribution test

| **Variable** | **Obs** | **Pr(skewness)** | **Pr(kurtosis)** | **—Joint teat—** | |
| --- | --- | --- | --- | --- | --- |
|  |  |  |  | Adj chi2(2) | Prob>chi2 |
| ΔGM mean | 14 | 0.4291 | 0.5499 | 1.09 | 0.0507 **^#^** |
| ΔCK mean | 14 | 0.7802 | 0.1590 | 2.39 | 0.3022 **^#^** |

Obs, objectives; Adj chi2(2), the p value of the adjusted Chi-square test; Pr(skewness), probability of skewness; Pr(kurtosis), probability of kurtosis; **#**: p>0.05, weight gain data for mammals fed GM soybean and mammals fed non-GM soybean fits a normal distribution.

1. Consumption of GM soybean showed no statistically significant impact on mammalian weight gain.

| **Variable** | **Obs** | **Mean** | **Std. err.** | **Std. dev.** | **[95 % conf. interval]** | |
| --- | --- | --- | --- | --- | --- | --- |
| ΔGM mean | 14 | 235.85 | 33.82374 | 126.5568 | 162.7783 | 308.9217 |
| ΔCK mean | 14 | 271.5714 | 34.82487 | 130.3027 | 196.3369 | 346.806 |
| Combined | 28 | 253.7107 | 24.06651 | 127.348 | 204.3303 | 303.0911 |
| diff. |  | -35.72143 | 48.54706 |  | -135.1696 | 63.72679 |

Obs, objectives; diff, mean (Δ GM mean)-mean (Δ CK-mean); Std. err., standard error Welch`s degrees of freedom = 27.9745; Std. dev., standard Deviation; t = -0.7358, consumption of GM soybean no statistically significant impact on mammalian weight gain.
