# Supplementary material for: Comprehensive risk assessment revealed some physiological indicators responding to various GM-crop consumption
Source: GM Crops Food. 2025 Dec 19;17(1):2603726. doi: 10.1080/21645698.2025.2603726 (PMC12721096; doi:10.1080/21645698.2025.2603726)

**Relative organ weight after GM-maize consumption**

**Figure S1** Consuming GM maize showed no statistically significant impact on mammalian relative brain weight


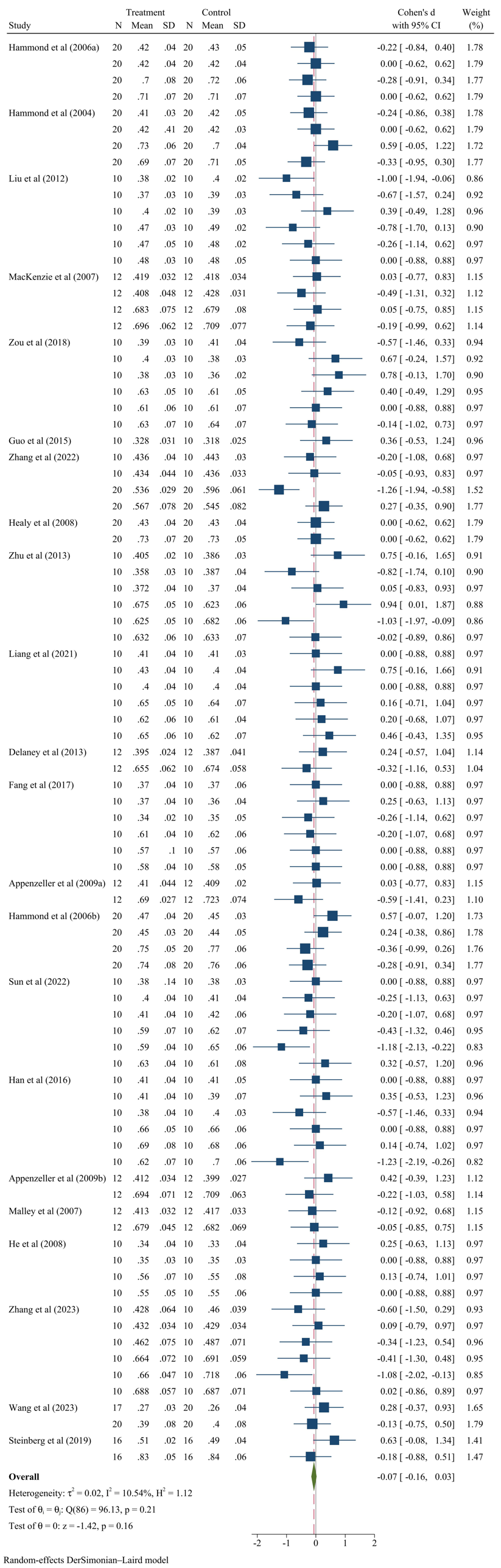


**Figure S2** Consuming GM maize showed no statistically significant impact on mammalian relative lung weight


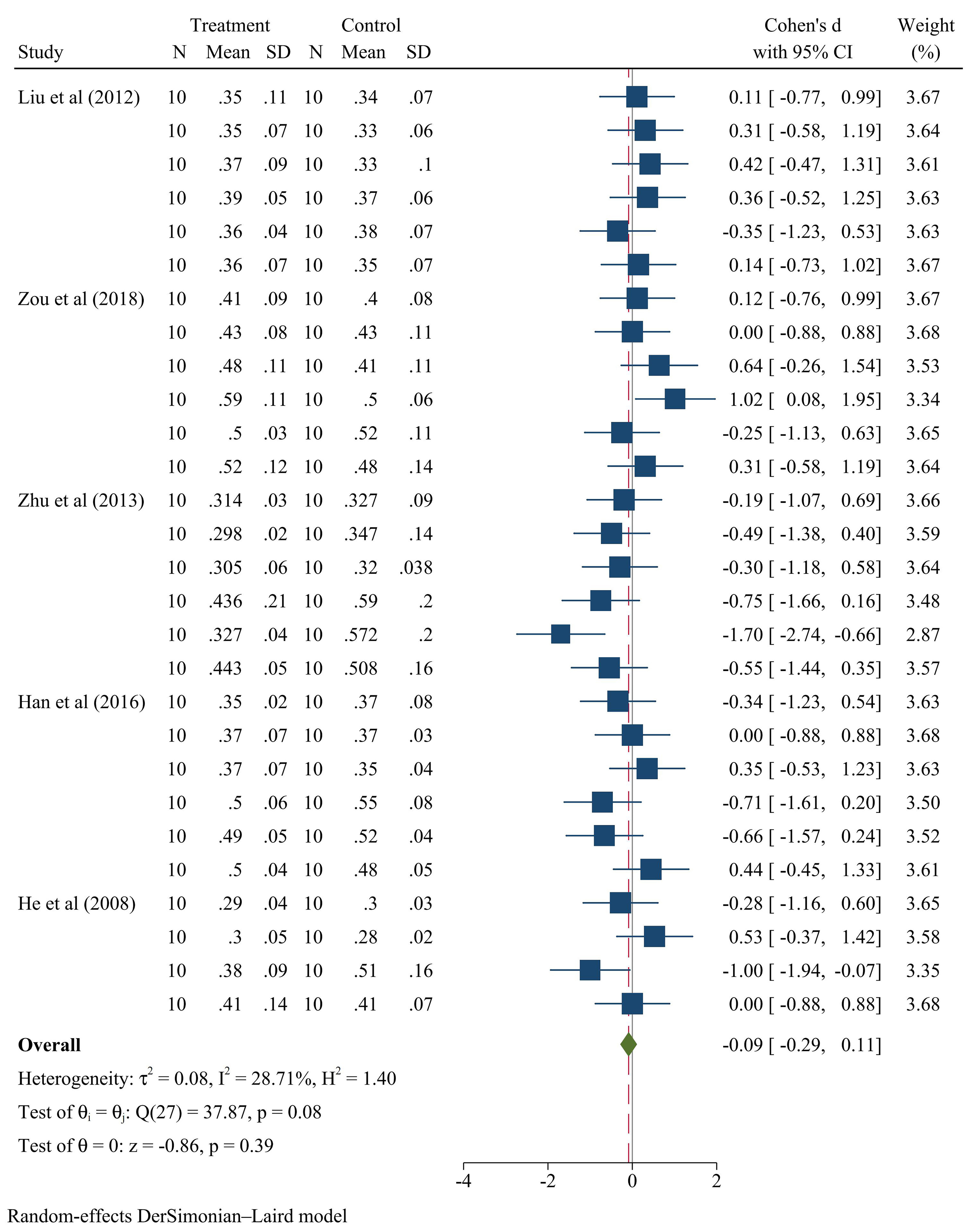


**Figure S3** Consuming GM maize showed no statistically significant impact on mammalian relative kidney weight


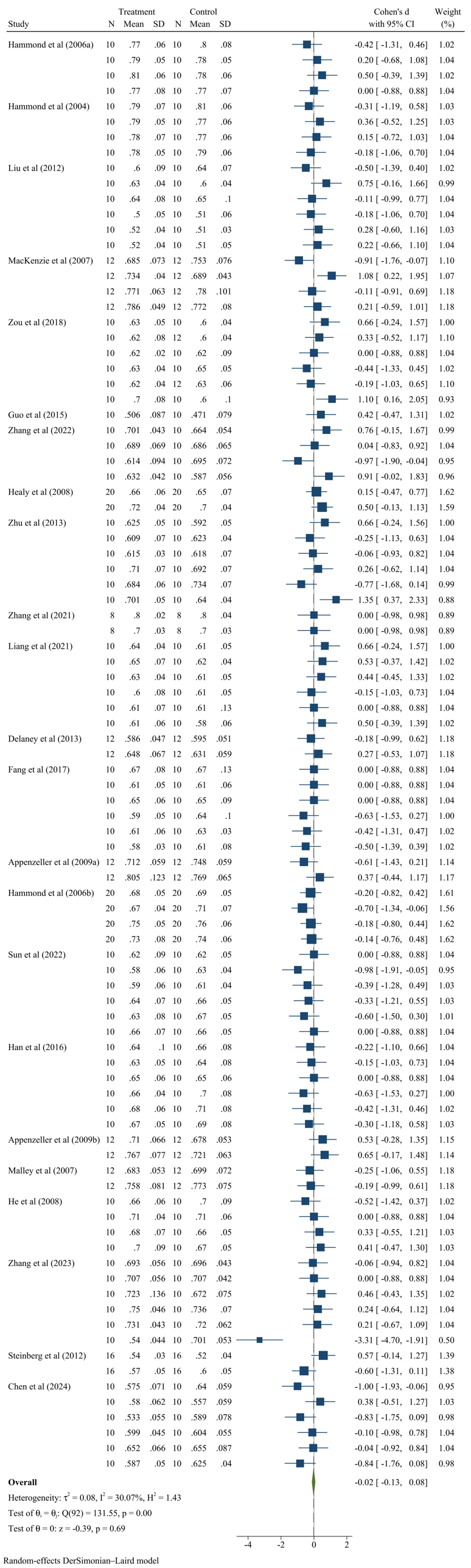


**Figure S4** Consuming GM maize showed no statistically significant impact on mammalian relative spleen weight


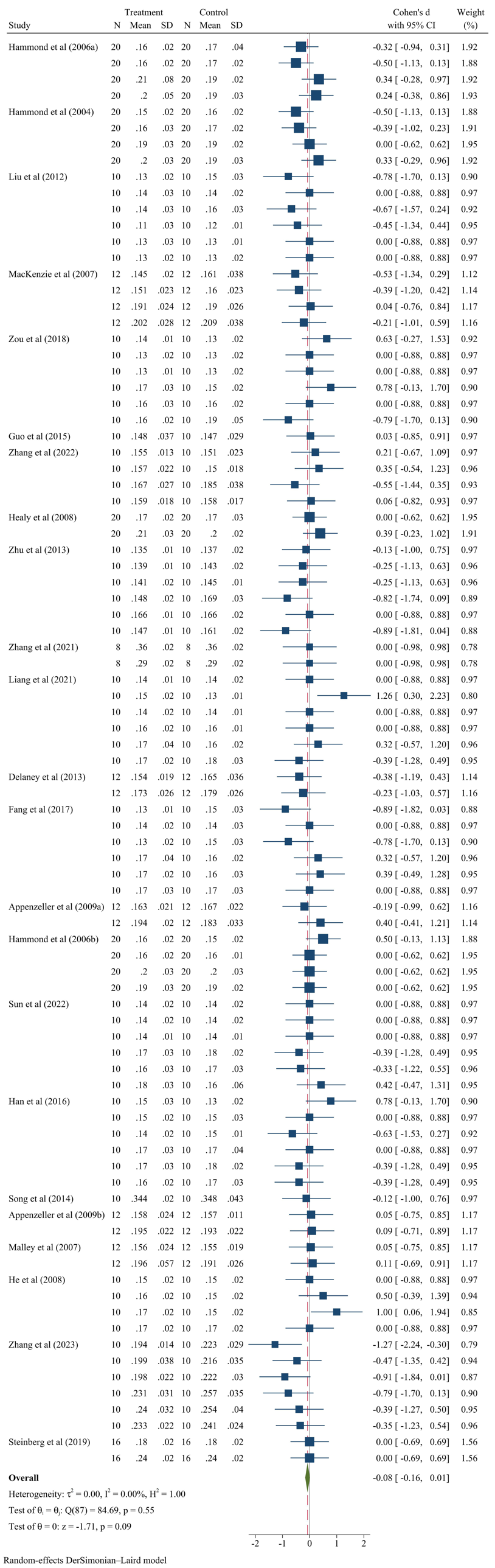


**Figure S5** Consuming GM maize showed no statistically significant impact on mammalian relative heart weight


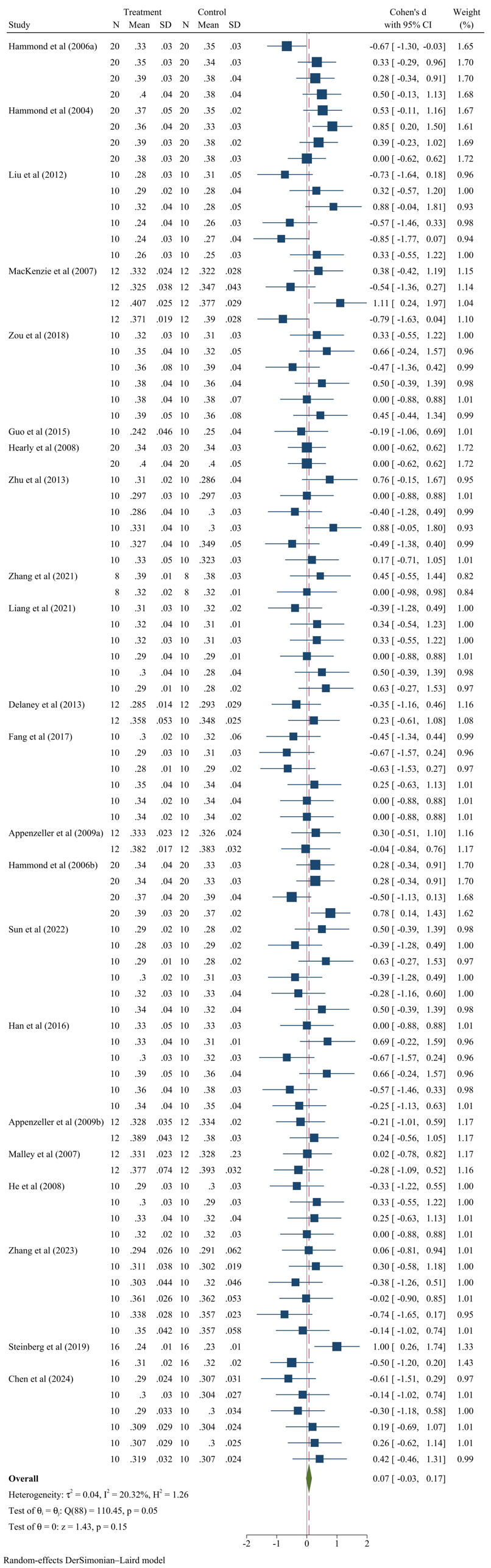


**Figure S6** Consuming GM maize led to statistically significant increase on mammalian relative liver weight


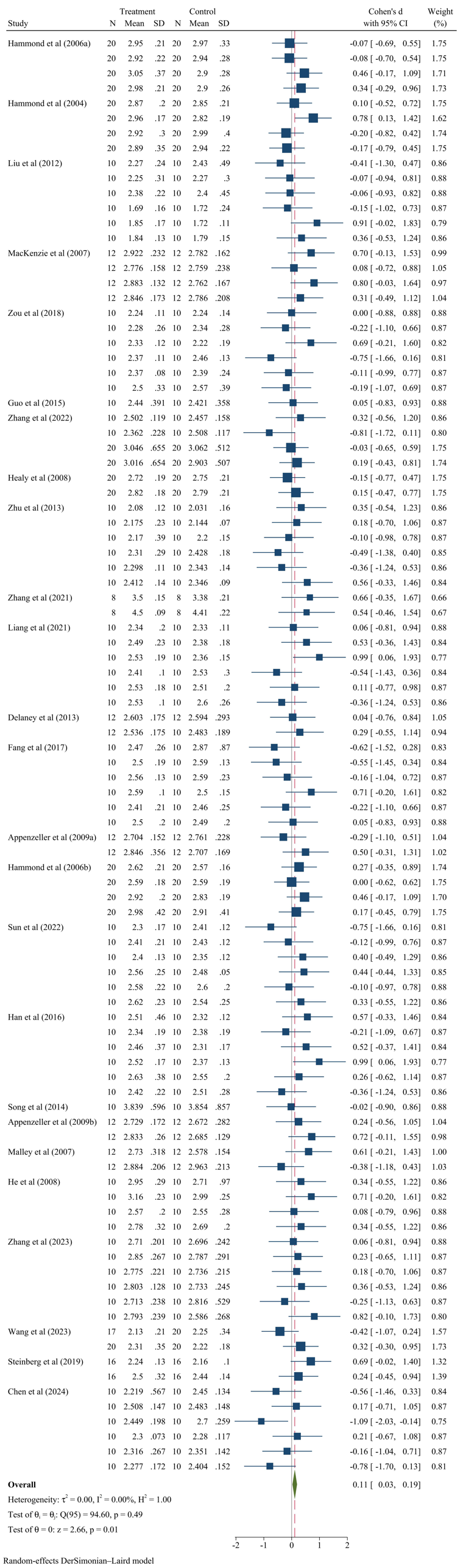


**Figure S7** Consuming low dose of GM maize showed no statistically significant impact on mammalian relative liver weight


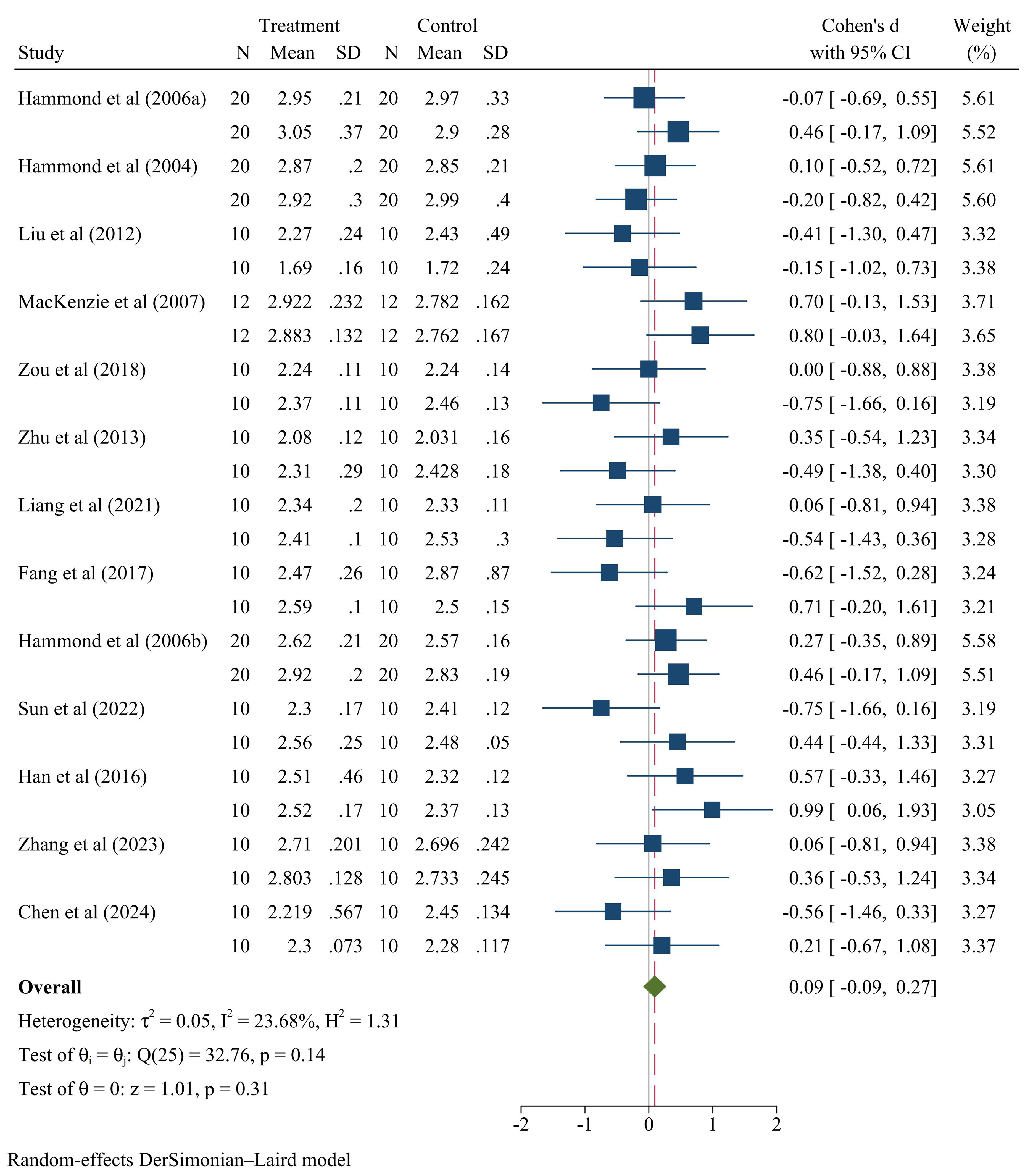


**Figure S8** Consuming medium dose of GM maize showed no statistically significant impact on mammalian relative liver weight


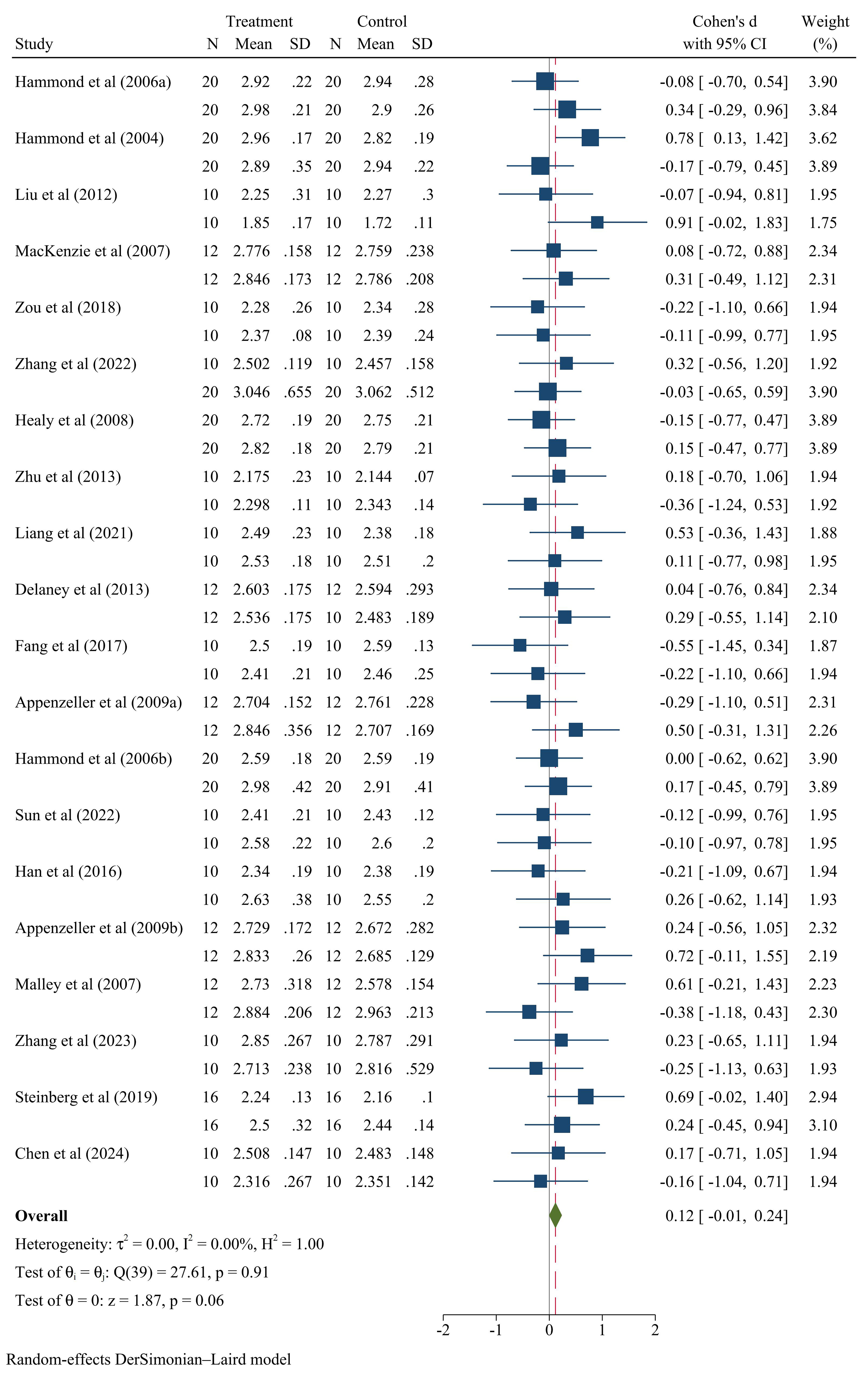


**Figure S9** Consuming high dose of GM maize showed no statistically significant impact on mammalian relative liver weight


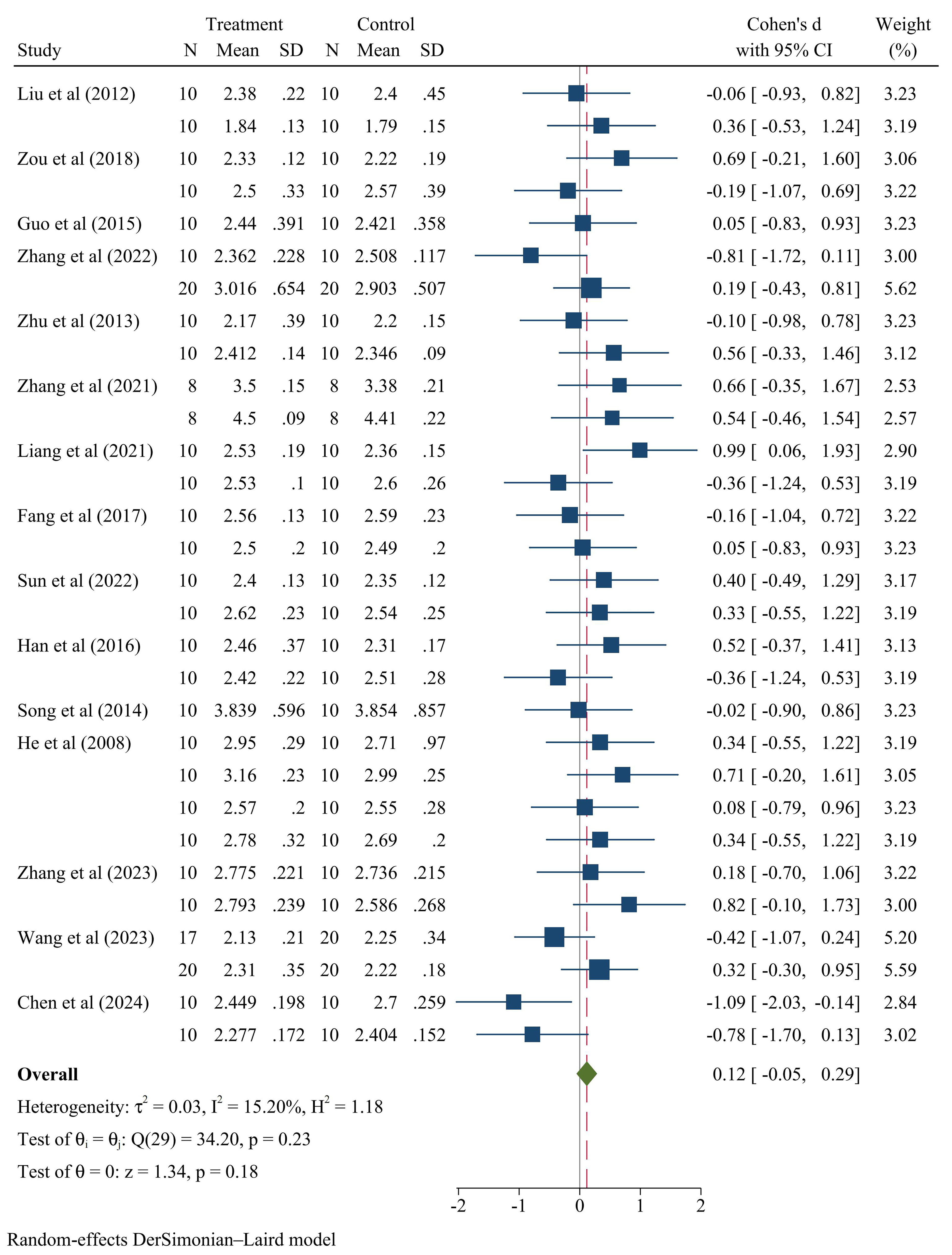


**Figure S10** Consuming GM maize showed no statistically significant impact on male mammalian relative liver weight


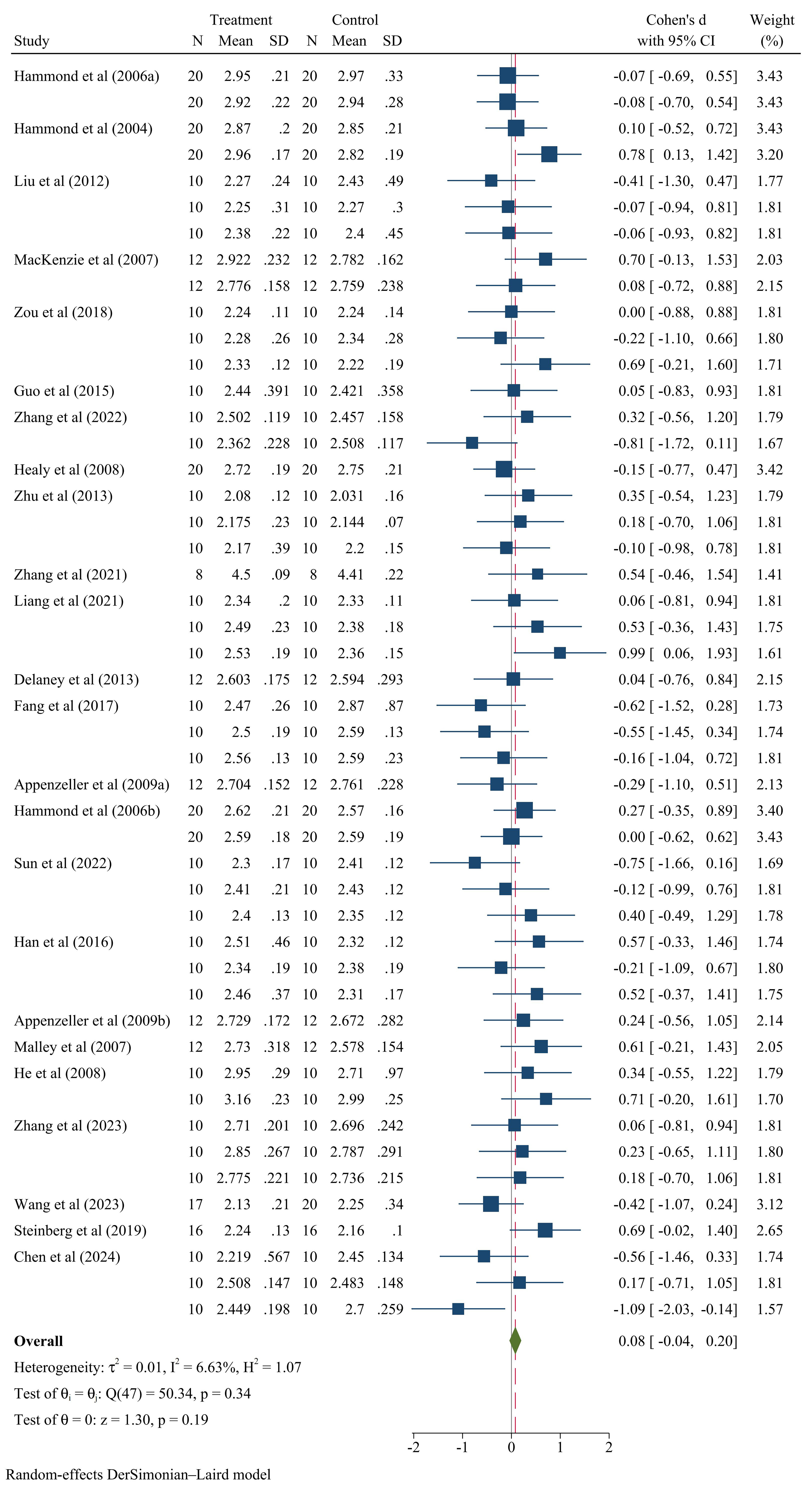


**Figure S11** Consuming GM maize led to statistically significant increase on female mammalian relative liver weight


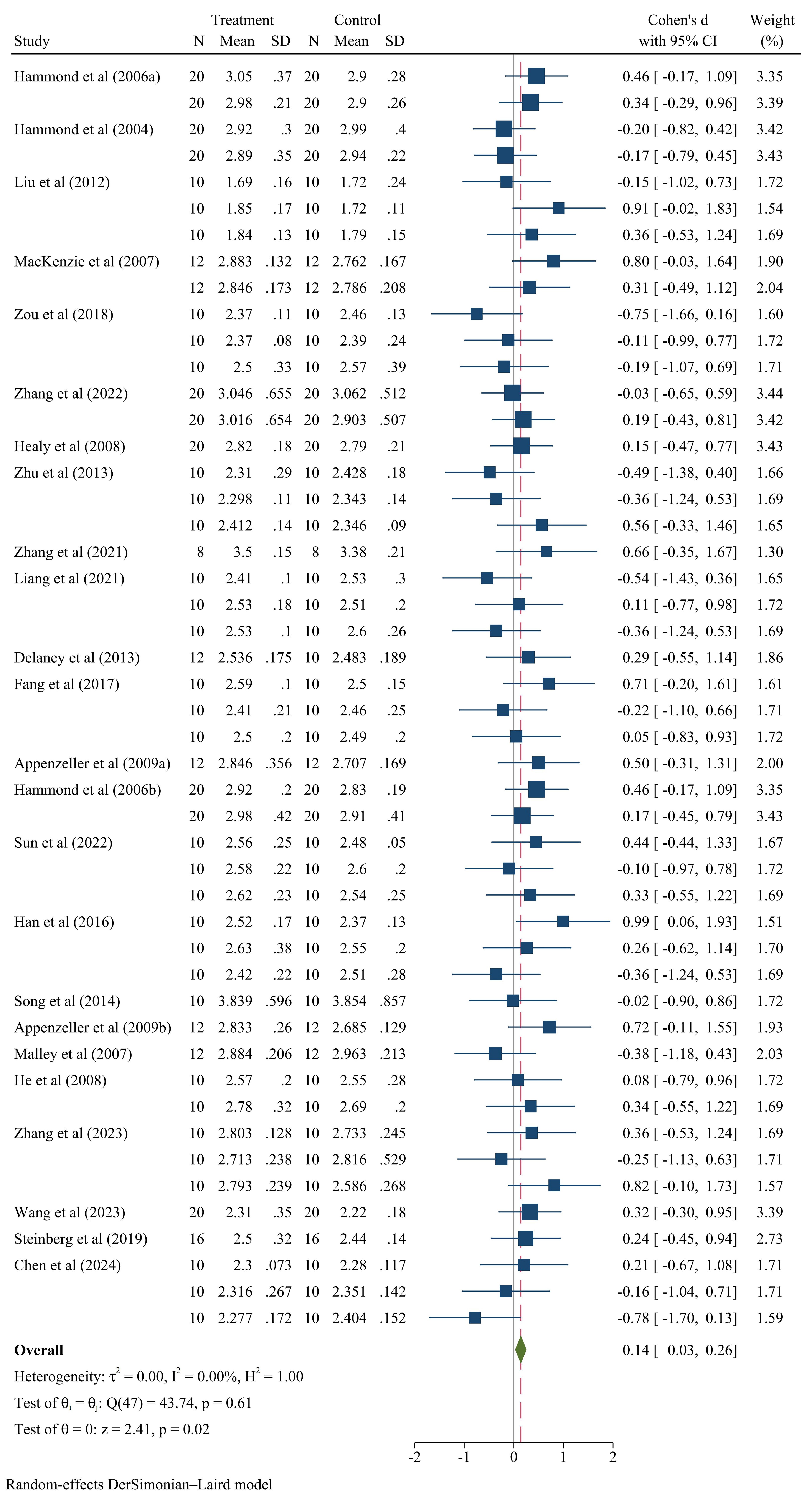

Supplement: Supplementary Figure S1 to S11.docx [file KGMC_A_2603726_SM6464.docx]
