# Supplementary material for: Comprehensive risk assessment revealed some physiological indicators responding to various GM-crop consumption
Source: GM Crops Food. 2025 Dec 19;17(1):2603726. doi: 10.1080/21645698.2025.2603726 (PMC12721096; doi:10.1080/21645698.2025.2603726)

**Indicators of liver function after GM crops consumption**

**Figure S85** Consuming GM maize showed no statistically significant impact on mammalian ALT concentration.


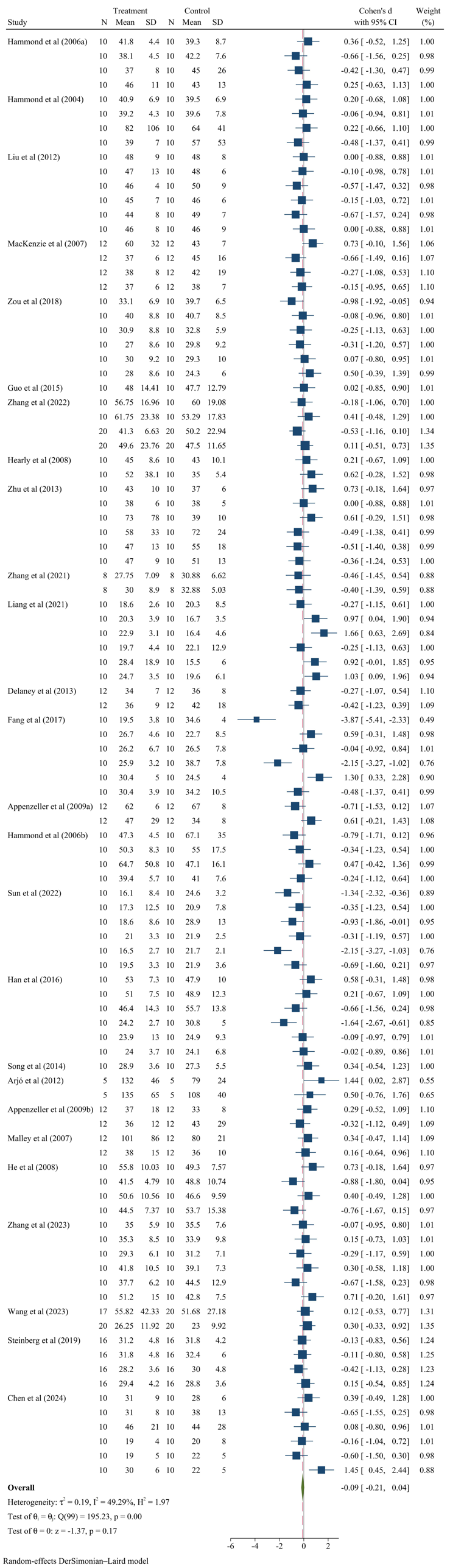


**Figure S86** Consuming GM maize showed no statistically significant impact on mammalian AST concentration.


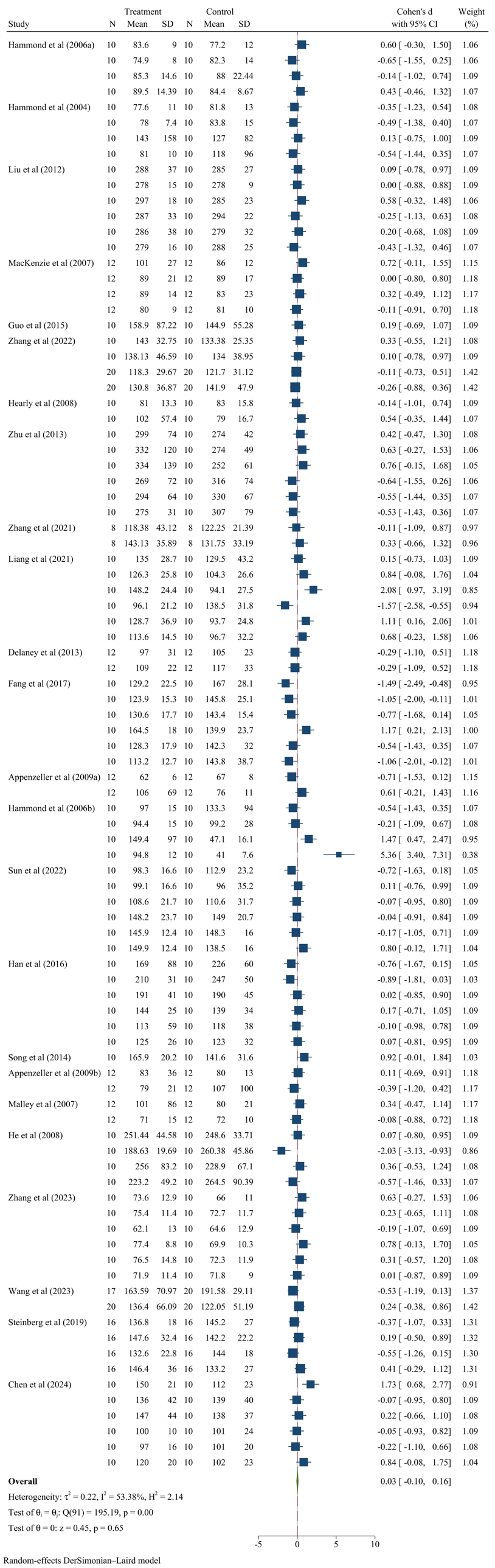


**Figure S87** Consuming GM rice showed no statistically significant impact on mammalian ALT concentration.


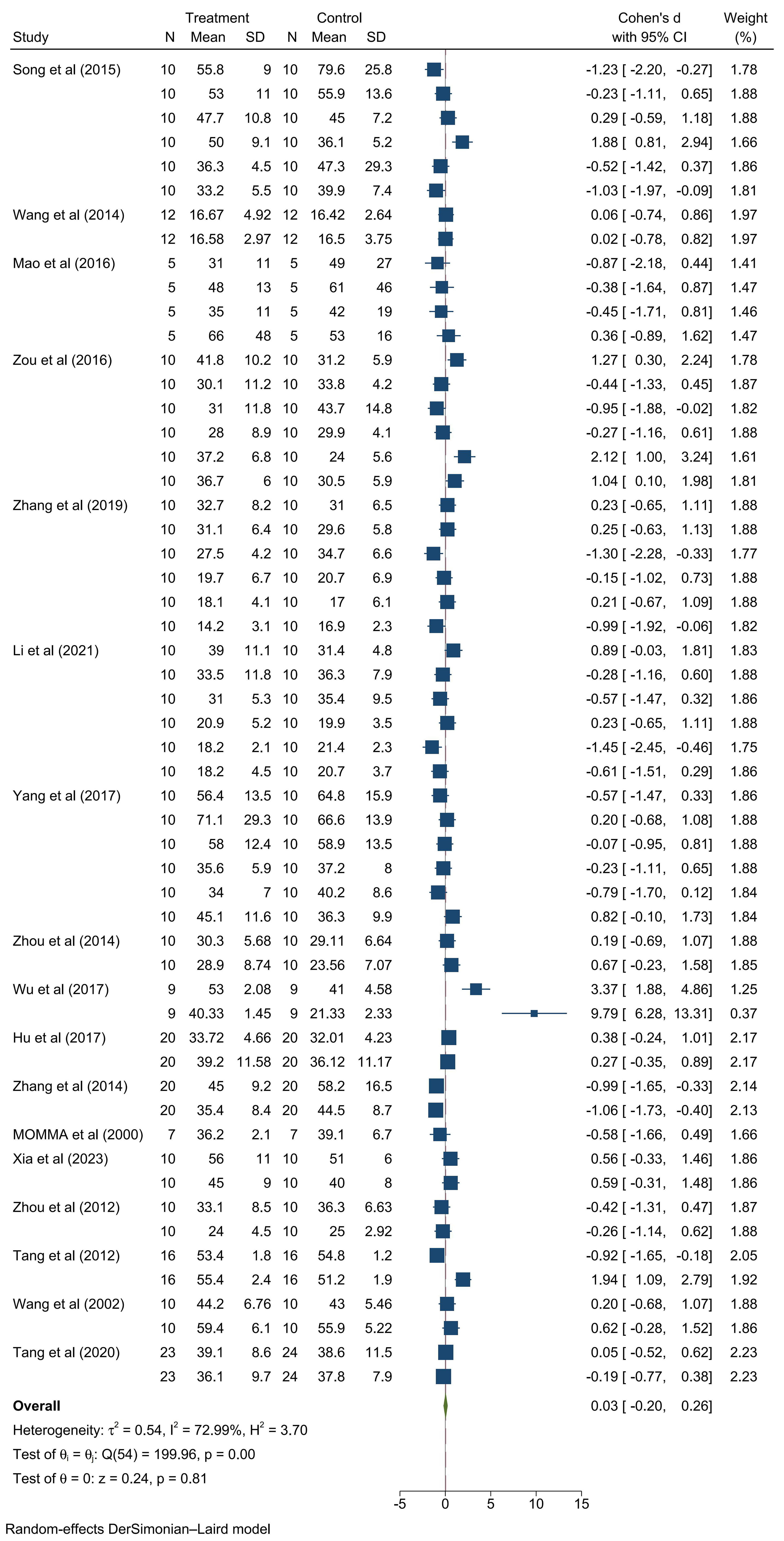


**Figure S88** Consuming GM rice showed no statistically significant impact on mammalian AST concentration.


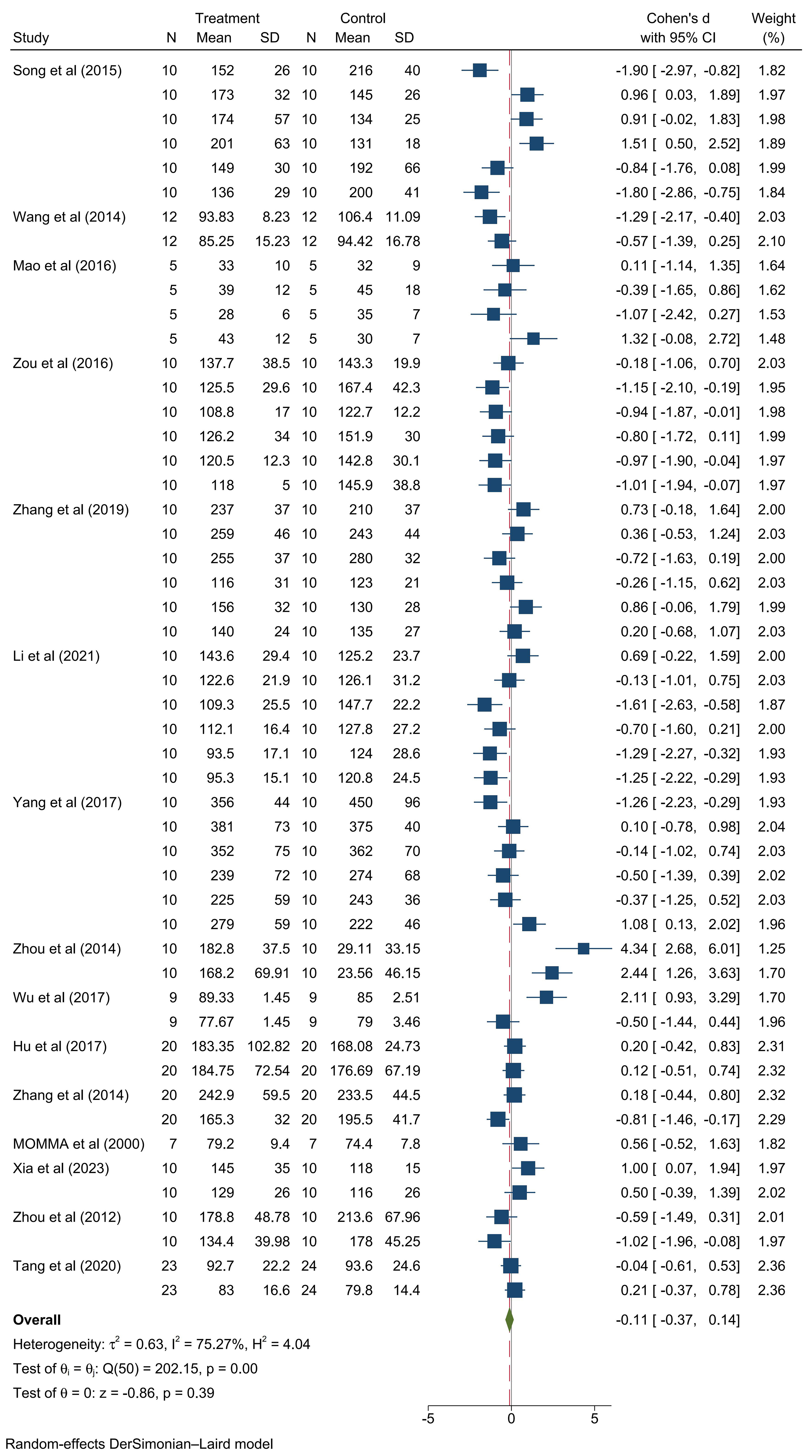


**Figure S89** Consuming GM soybean showed no statistically significant impact on mammalian ALT concentration.


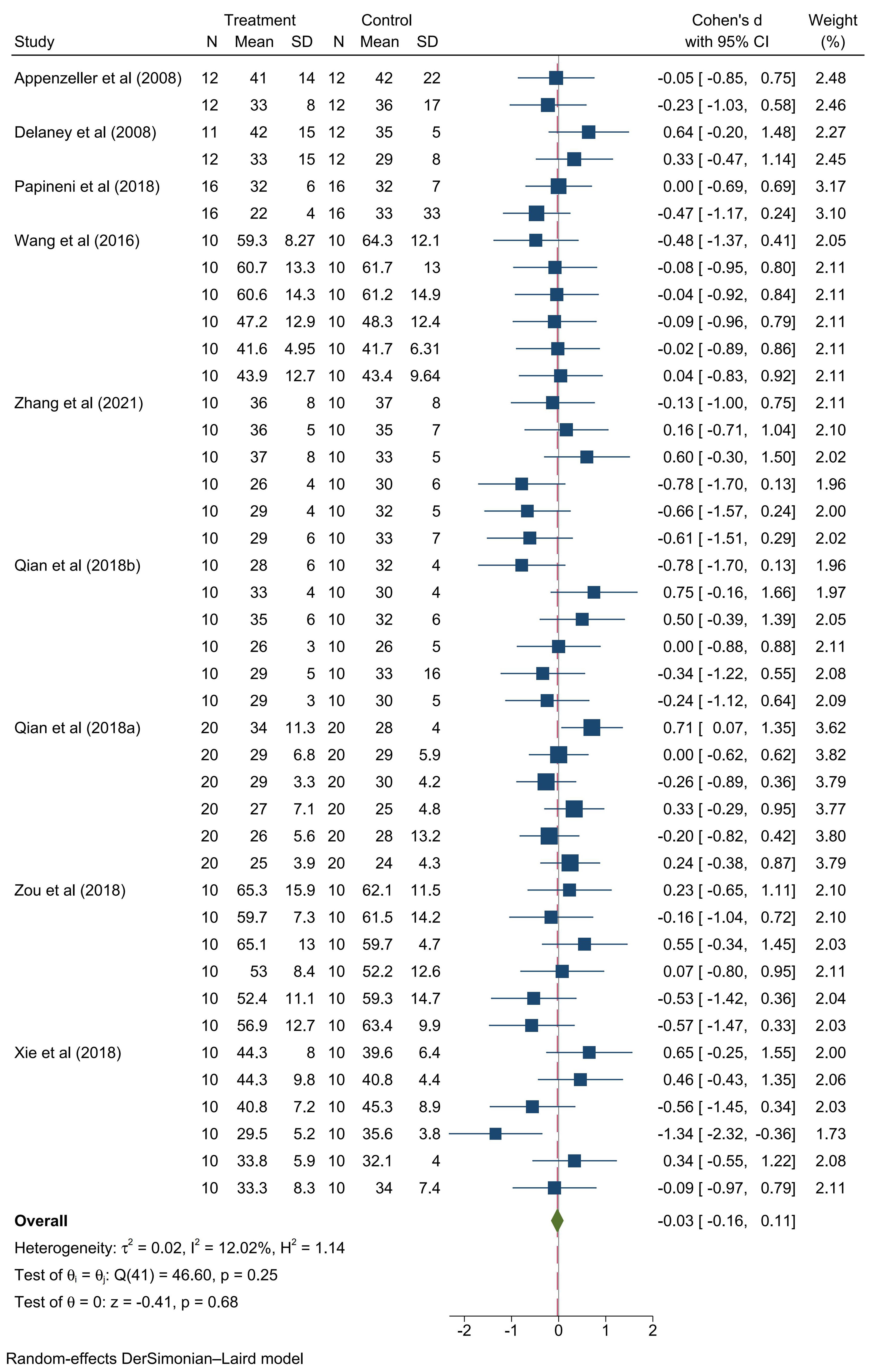


**Figure S90** Consuming GM soybean showed no statistically significant impact on mammalian AST concentration.


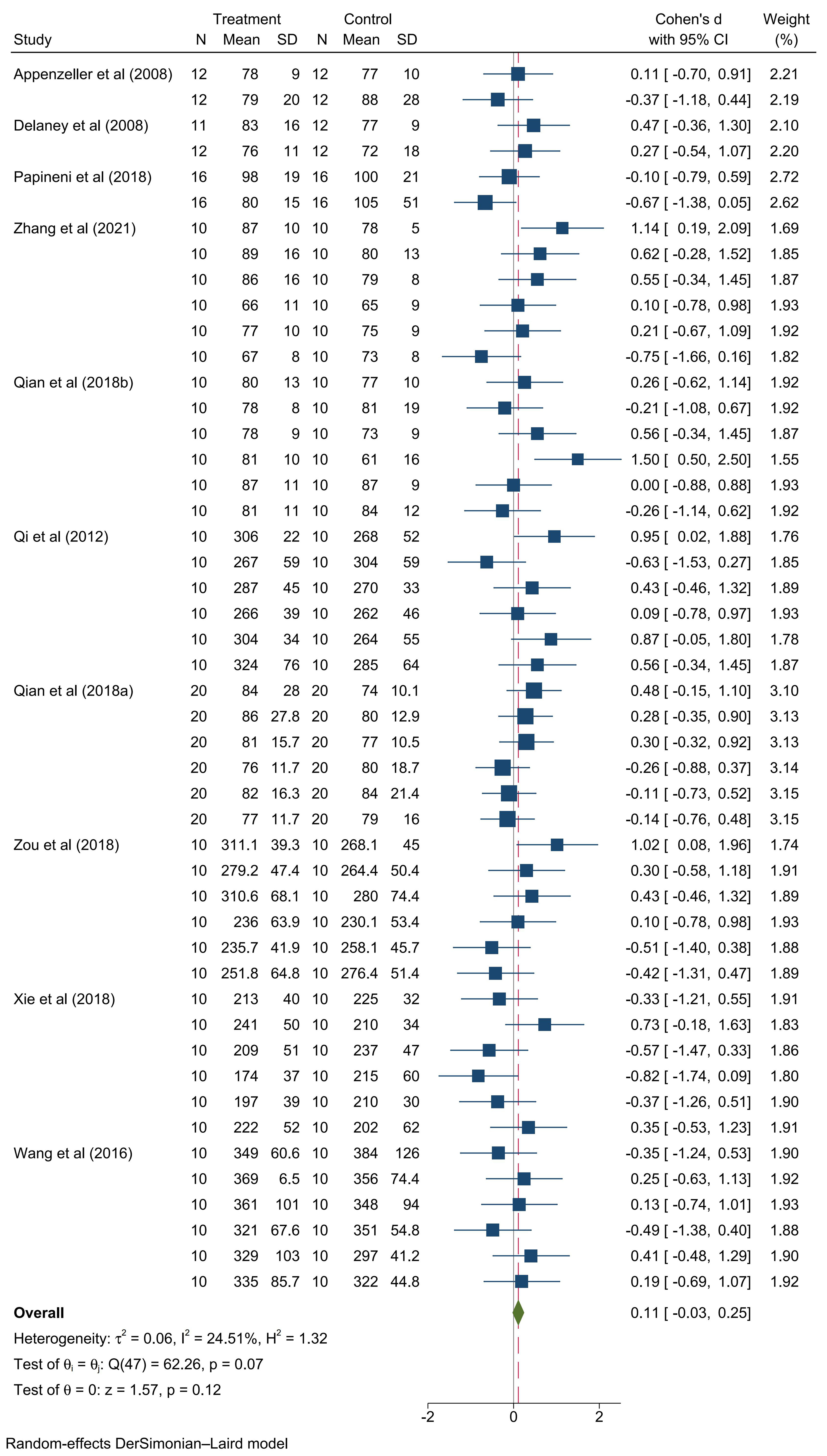


**Figure S91** Consuming GM maize showed no statistically significant impact on mammalian TBIL concentration.


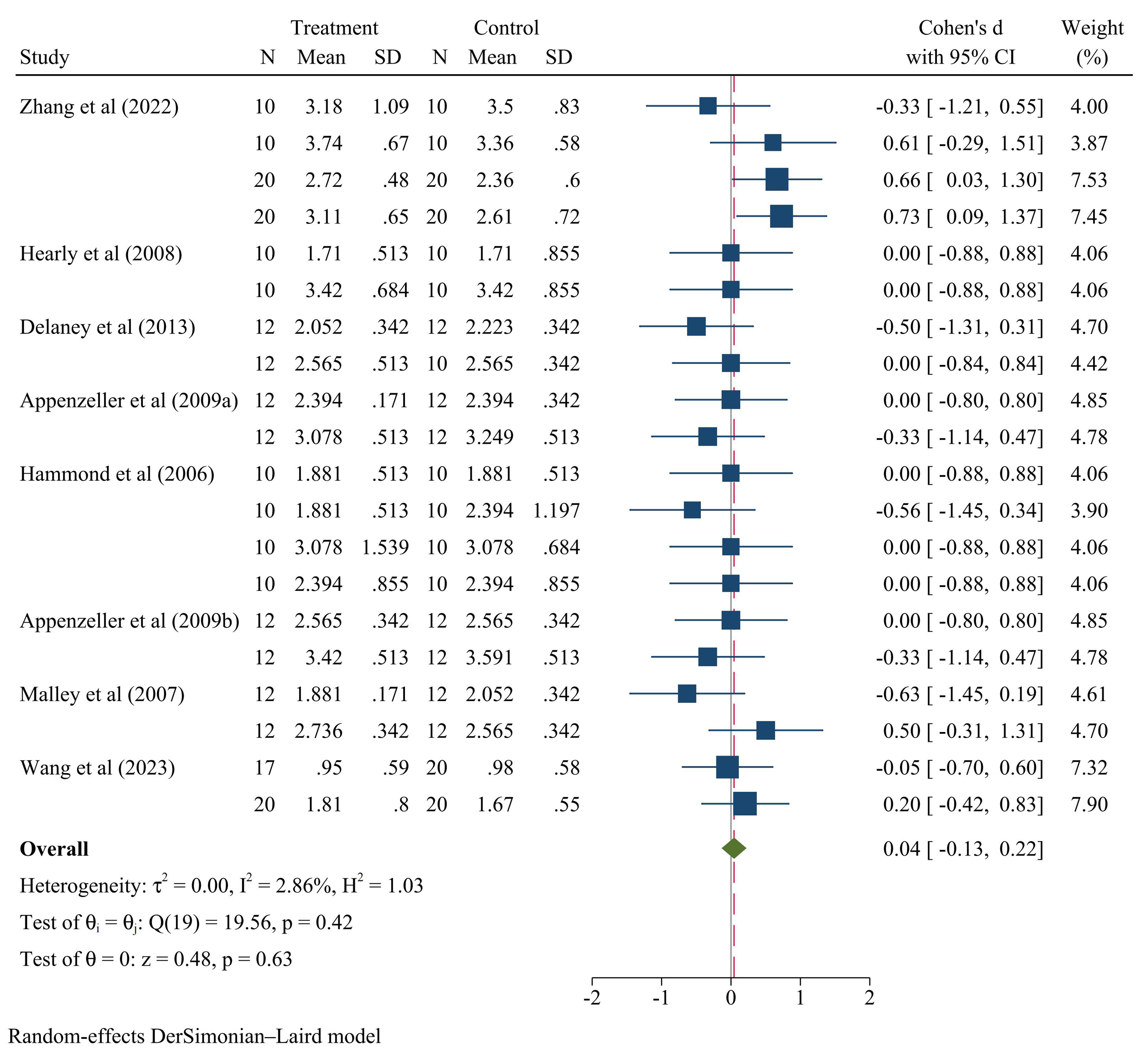

Supplement: Supplementary Figure S85 to S91.docx [file KGMC_A_2603726_SM6463.docx]
