# Supplementary material for: Comprehensive risk assessment revealed some physiological indicators responding to various GM-crop consumption
Source: GM Crops Food. 2025 Dec 19;17(1):2603726. doi: 10.1080/21645698.2025.2603726 (PMC12721096; doi:10.1080/21645698.2025.2603726)
Supplement: Supplementary Figure S60 to S79.docx [file KGMC_A_2603726_SM6462.docx]

**Blood cells concentrations after GM crops consumption**

**Figure S60** Consuming GM maize showed no statistically significant impact on mammalian WBC concentration


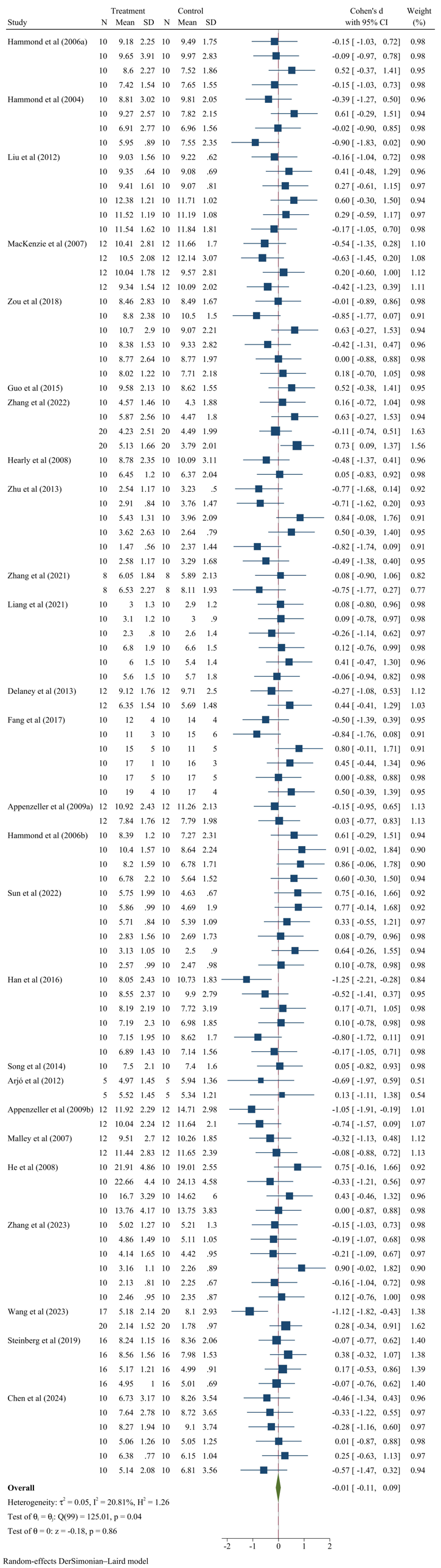


**Figure S61** Consuming GM rice showed no statistically significant impact on mammalian WBC concentration


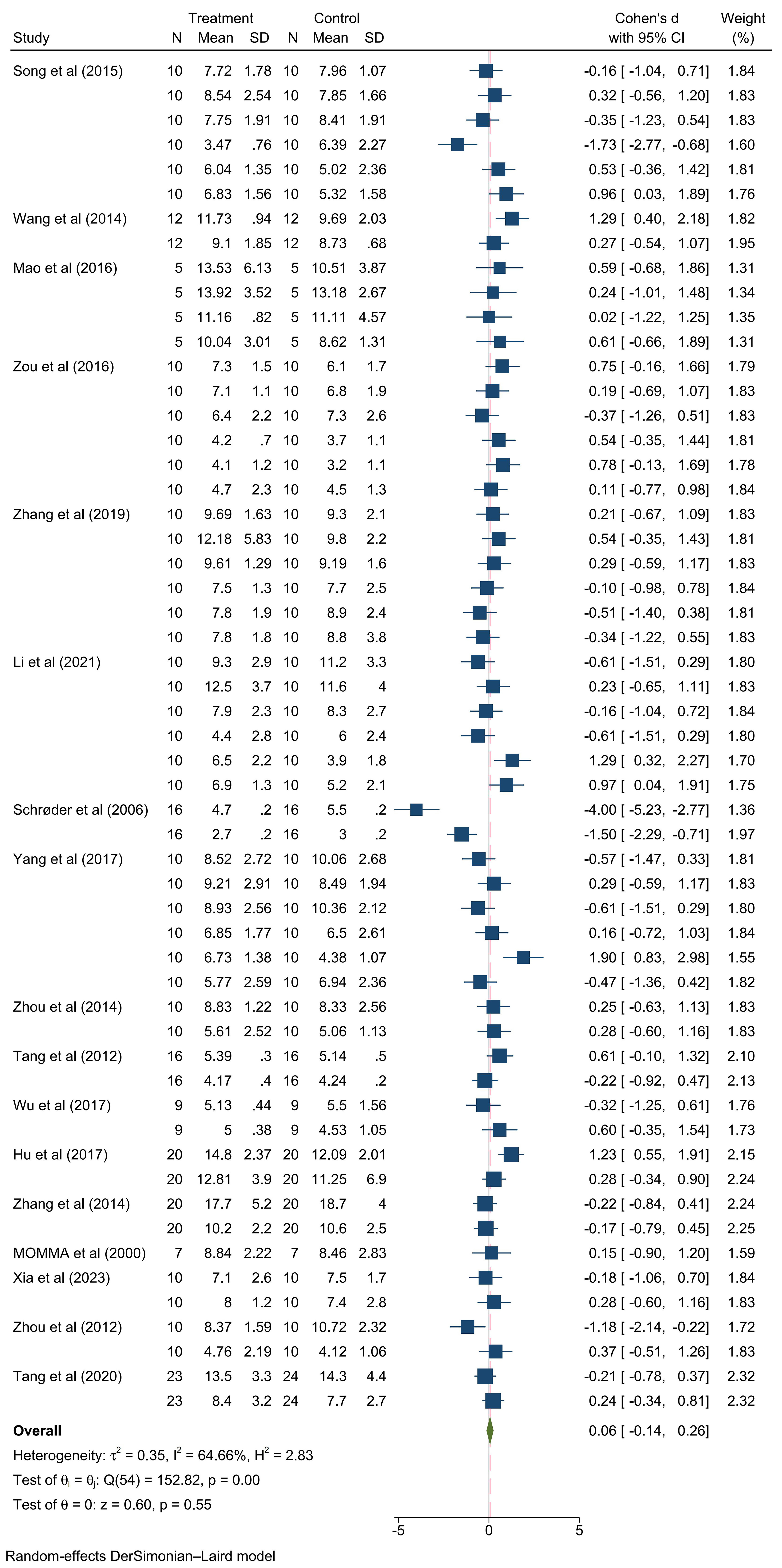


**Figure S62** Consuming GM soybean showed no statistically significant impact on mammalian WBC concentration


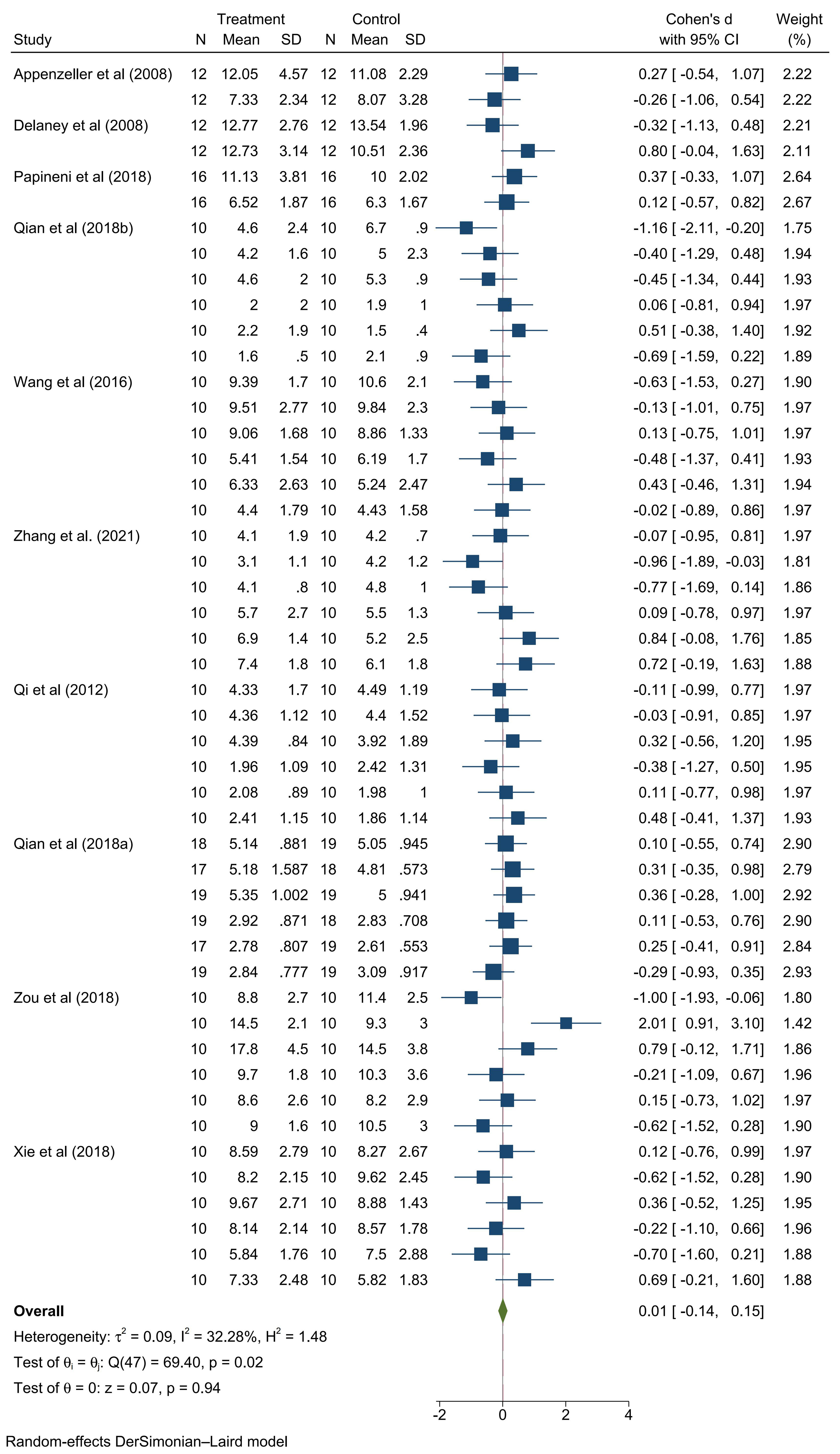


**Figure S63** Consuming GM maize showed no statistically significant impact on mammalian LYM concentration


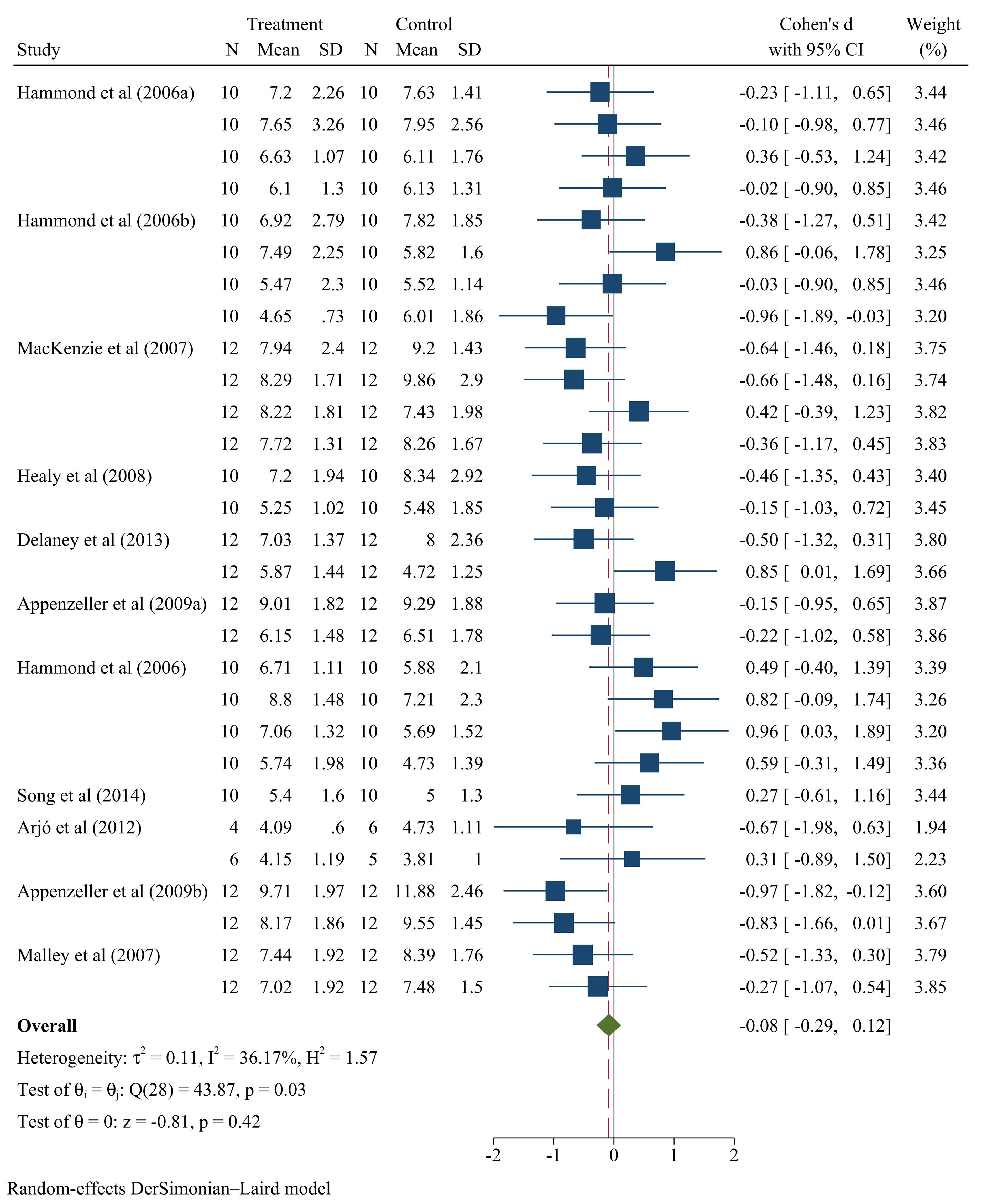


**Figure S64** Consuming GM rice showed no statistically significant impact on mammalian LYM concentration


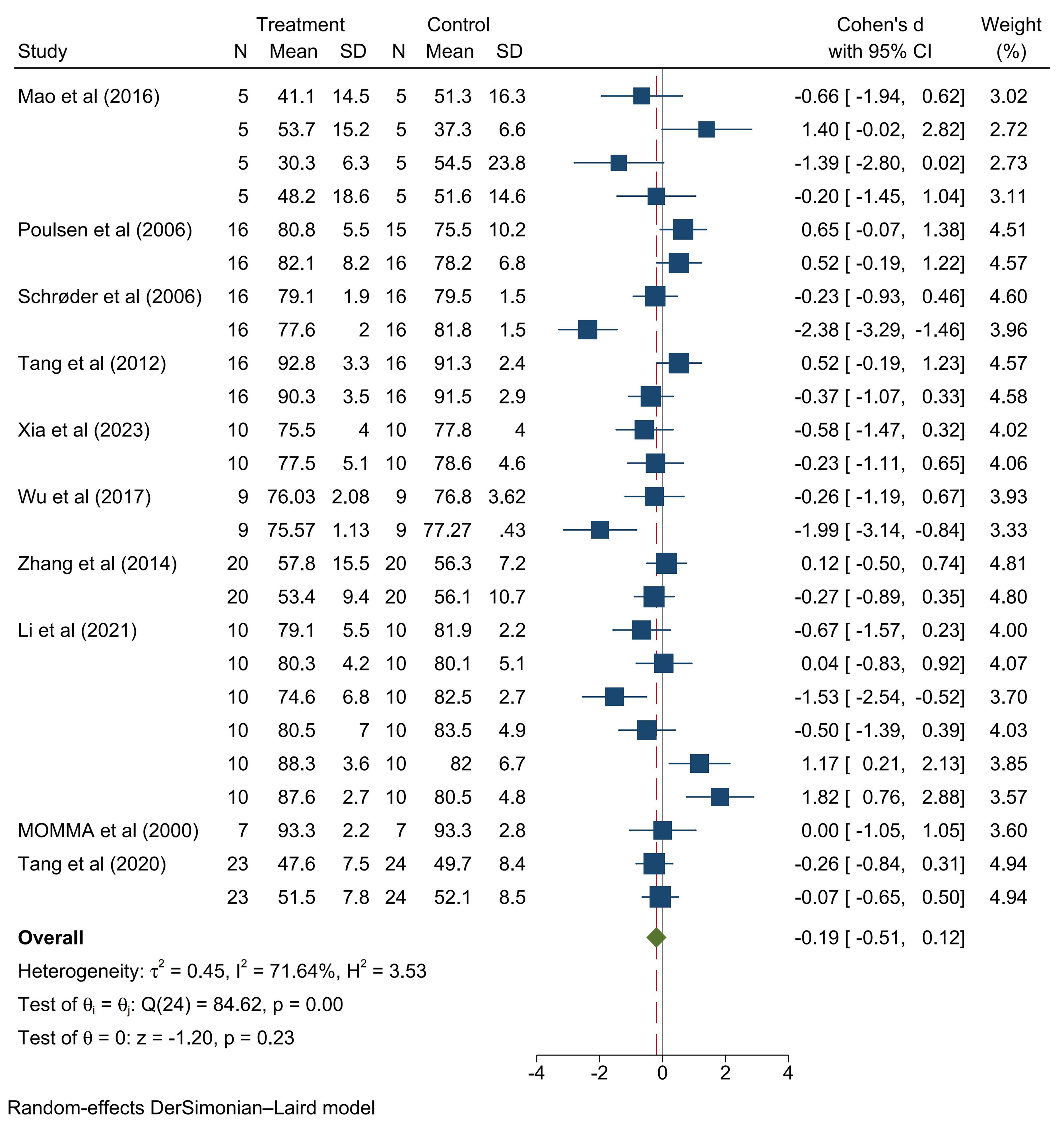


**Figure S65** Consuming GM soybean showed no statistically significant impact on mammalian LYM concentration


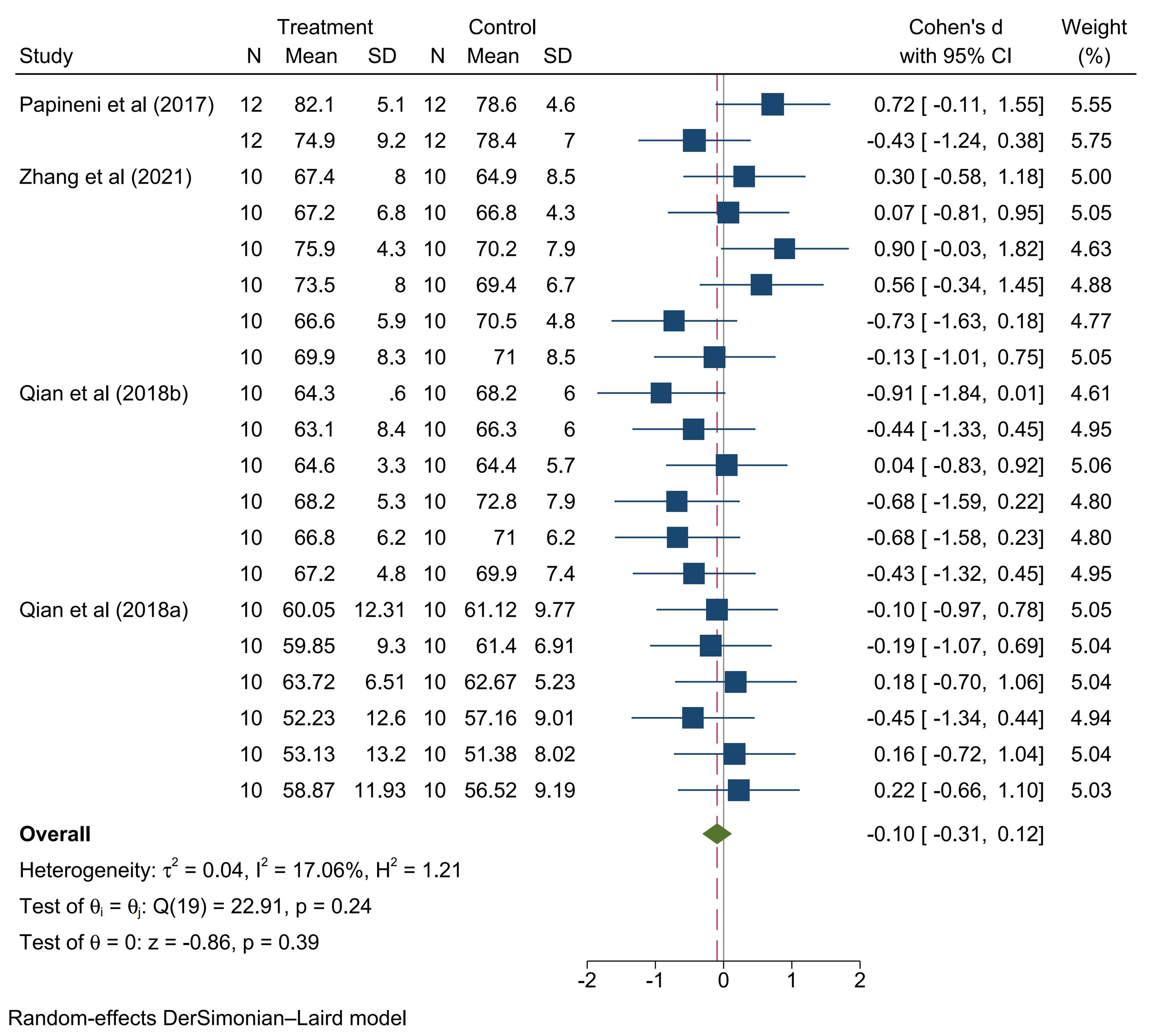


**Figure S66** Consuming GM maize showed no statistically significant impact on mammalian Neu concentration


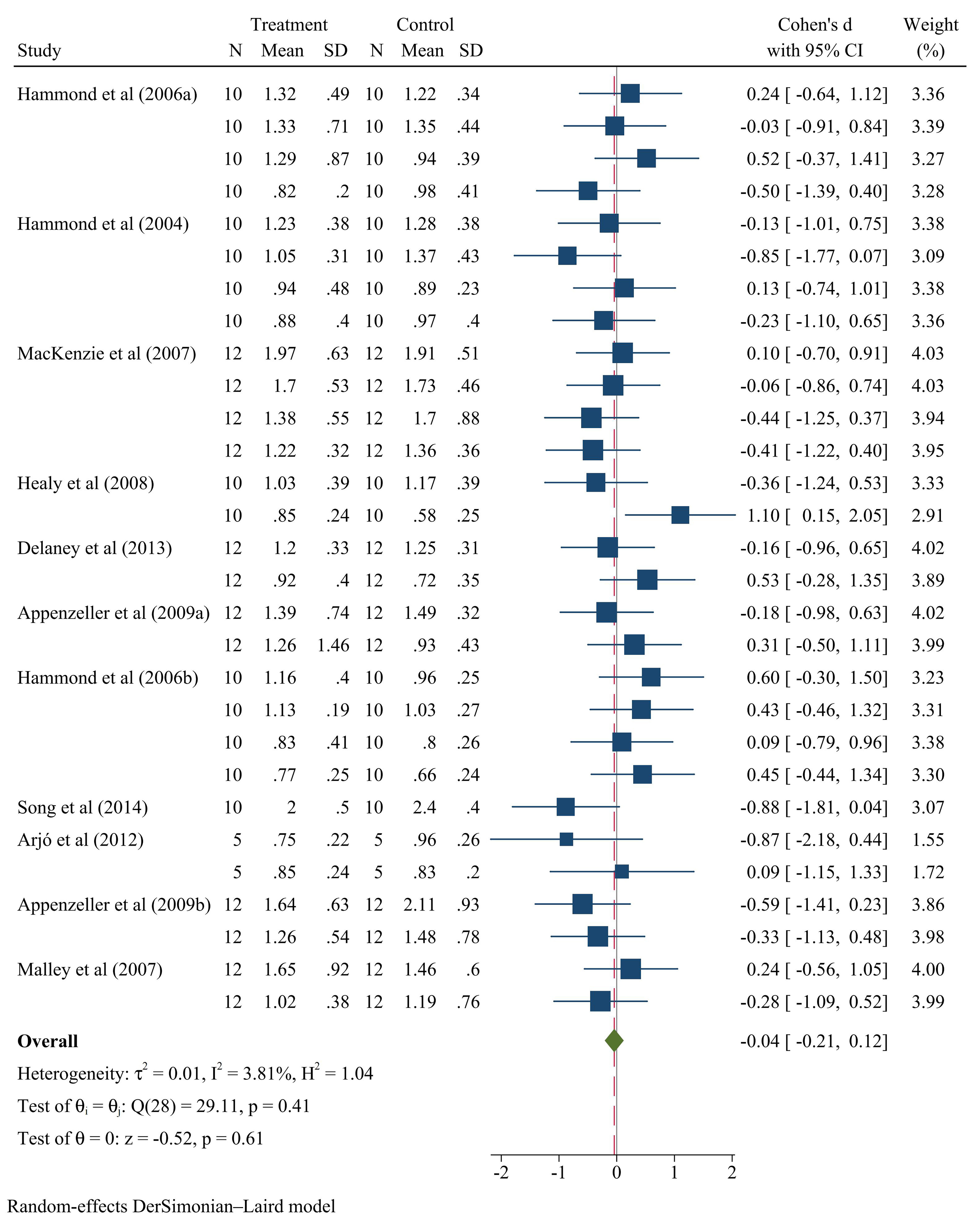


**Figure S67** Consuming GM rice showed no statistically significant impact on mammalian Neu concentration


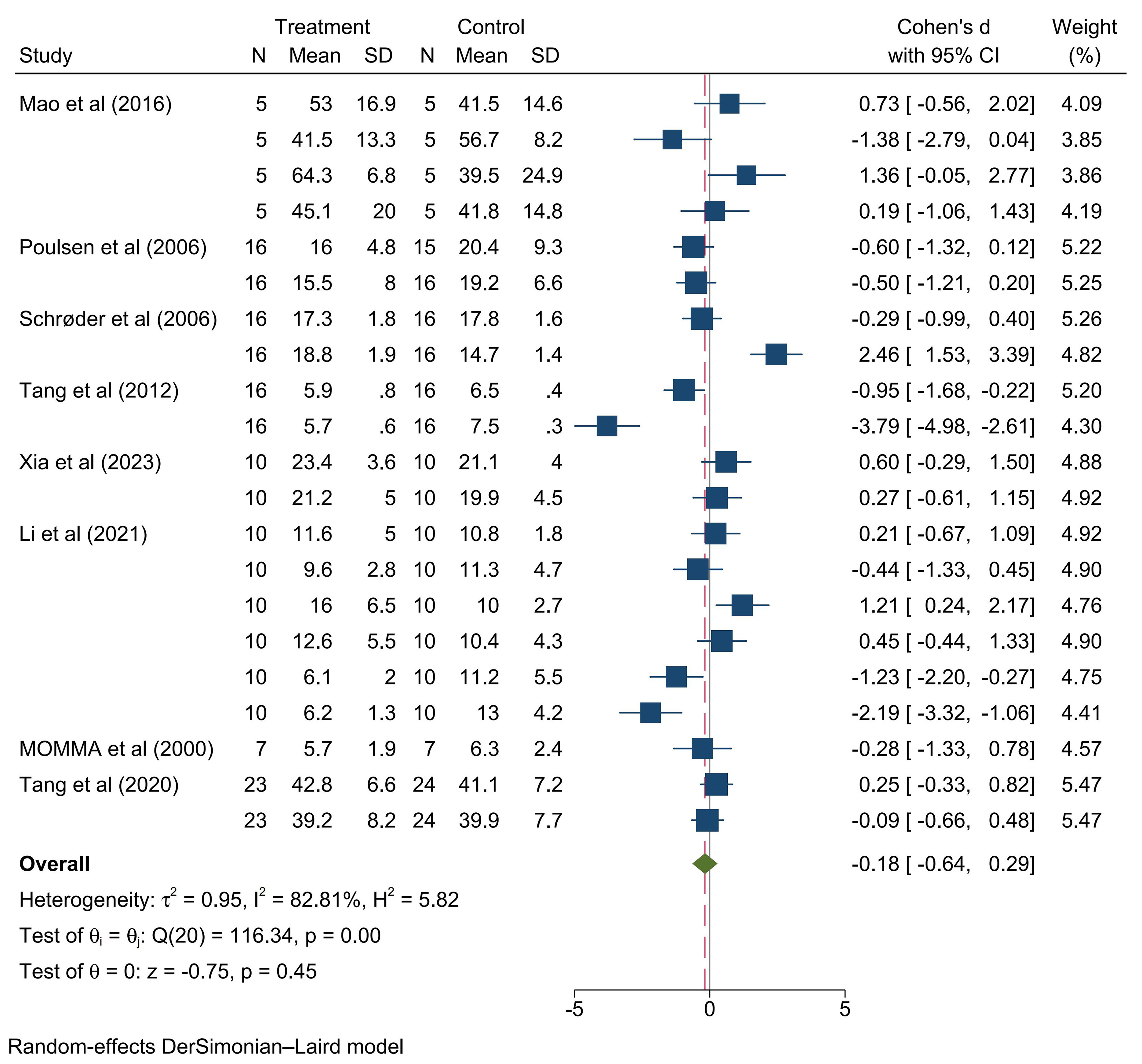


**Figure S68** Consuming GM soybean showed no statistically significant impact on mammalian Neu concentration


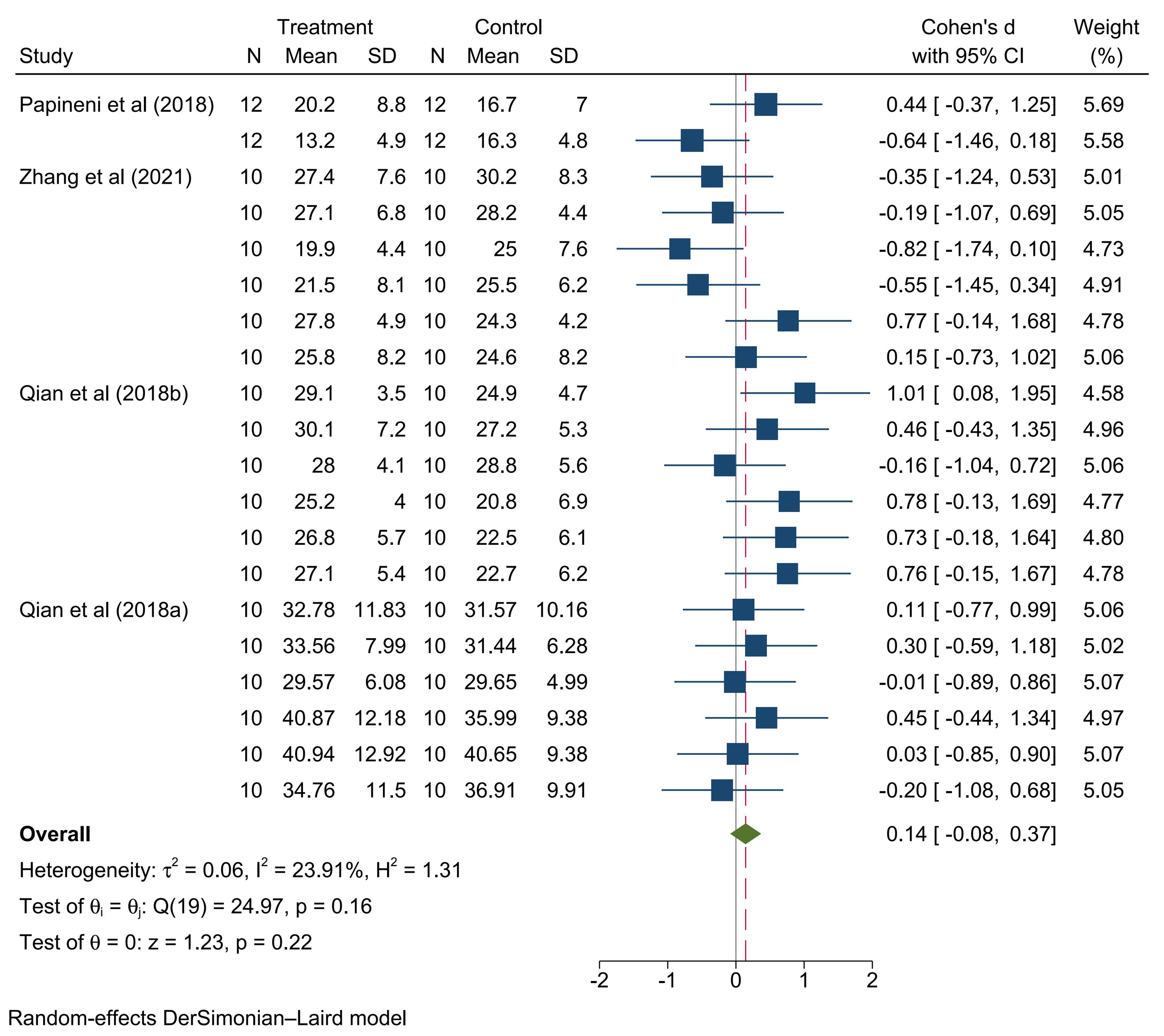


**Figure S69** Consuming GM maize showed no statistically significant impact on mammalian MON concentration


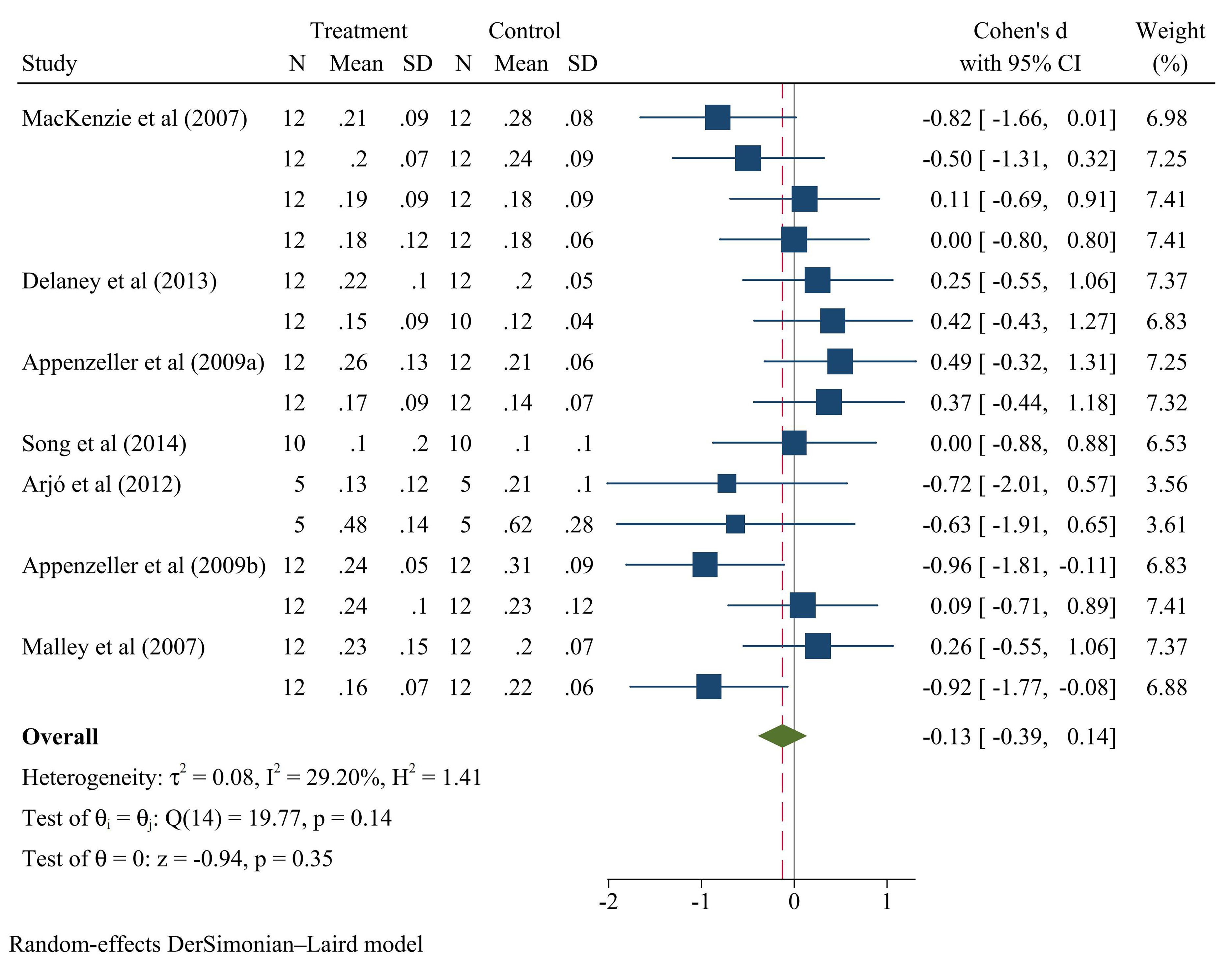


**Figure S70** Consuming GM rice showed no statistically significant impact on mammalian MON concentration


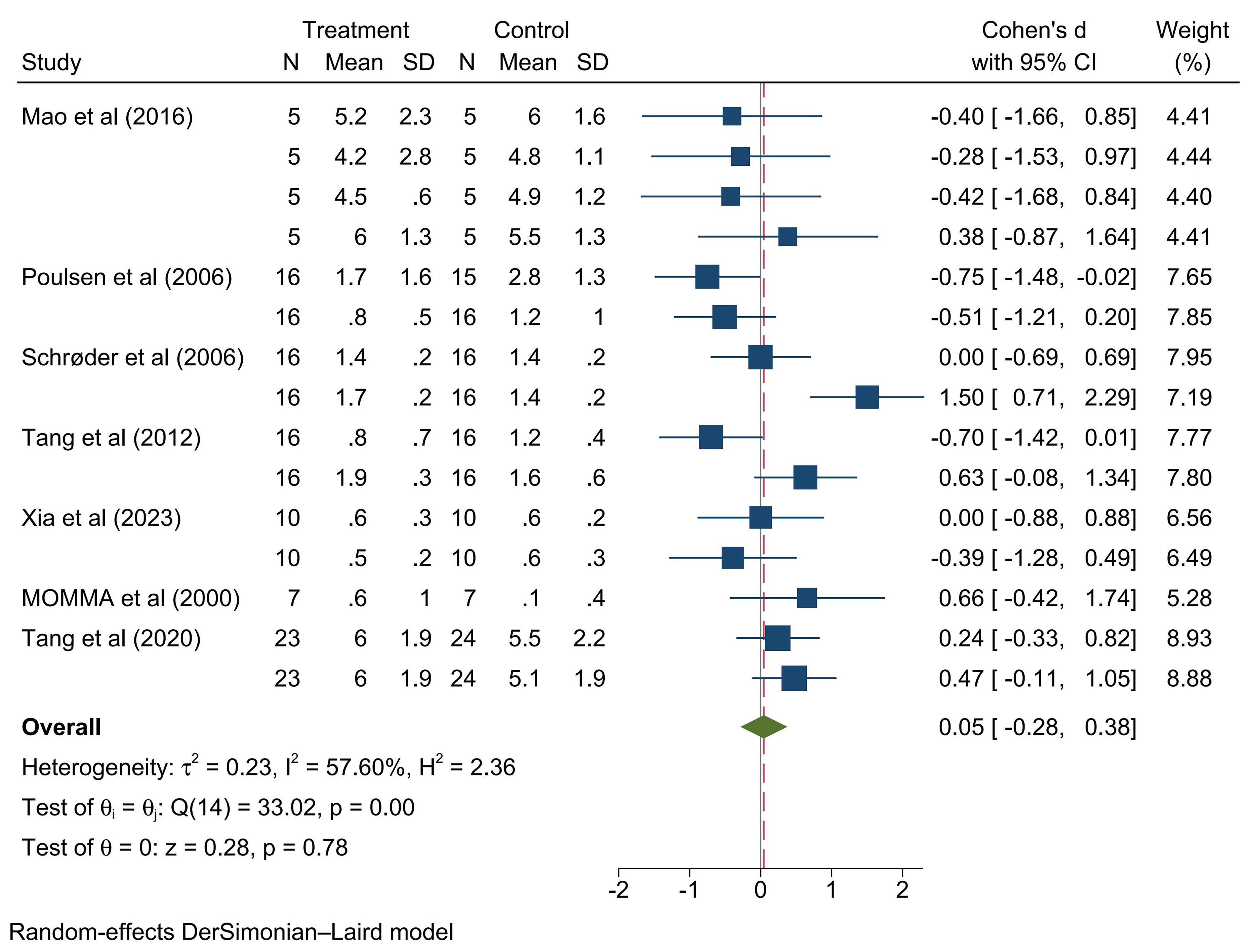


**Figure S71** Consuming GM maize showed no statistically significant impact on mammalian PLT concentration


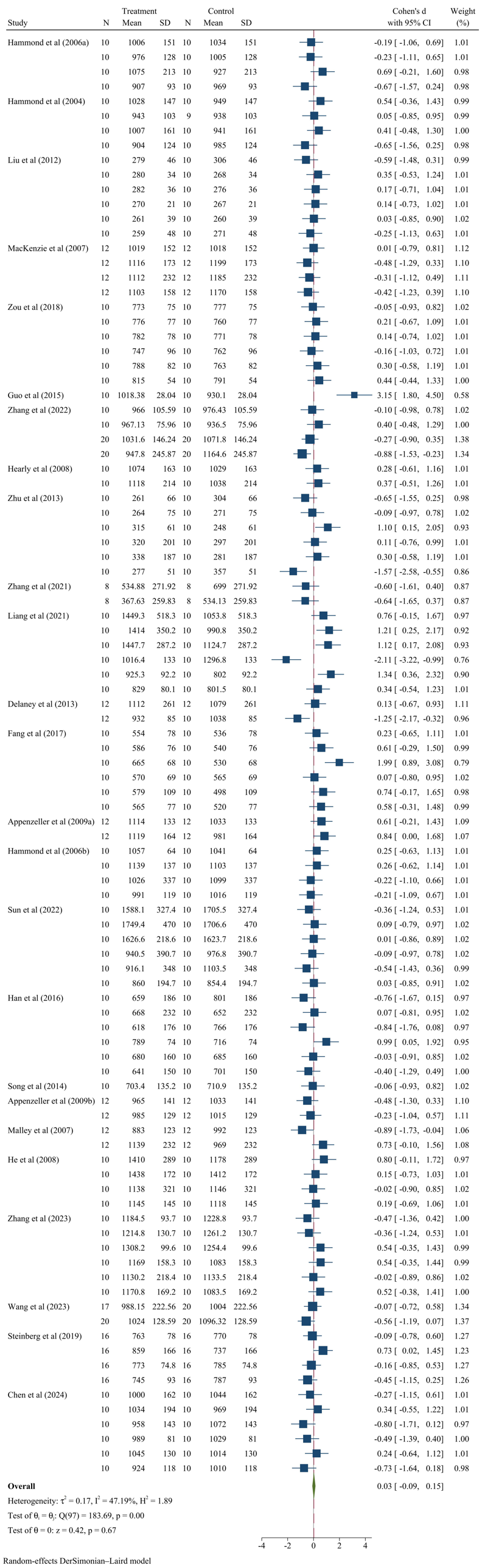


**Figure S72** Consuming GM rice showed no statistically significant impact on mammalian PLT concentration


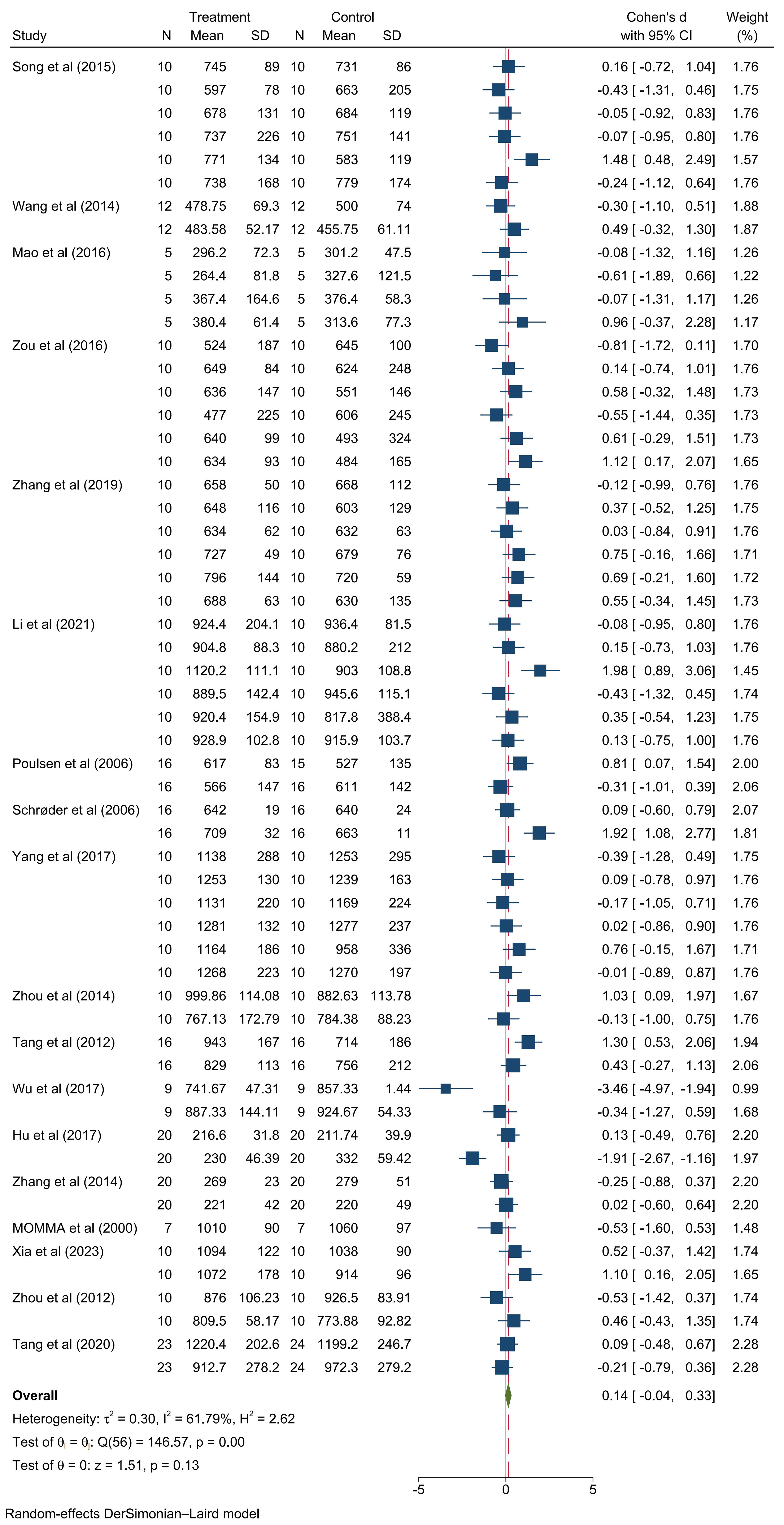


**Figure S73** Consuming GM soybean showed no statistically significant impact on mammalian PLT concentration


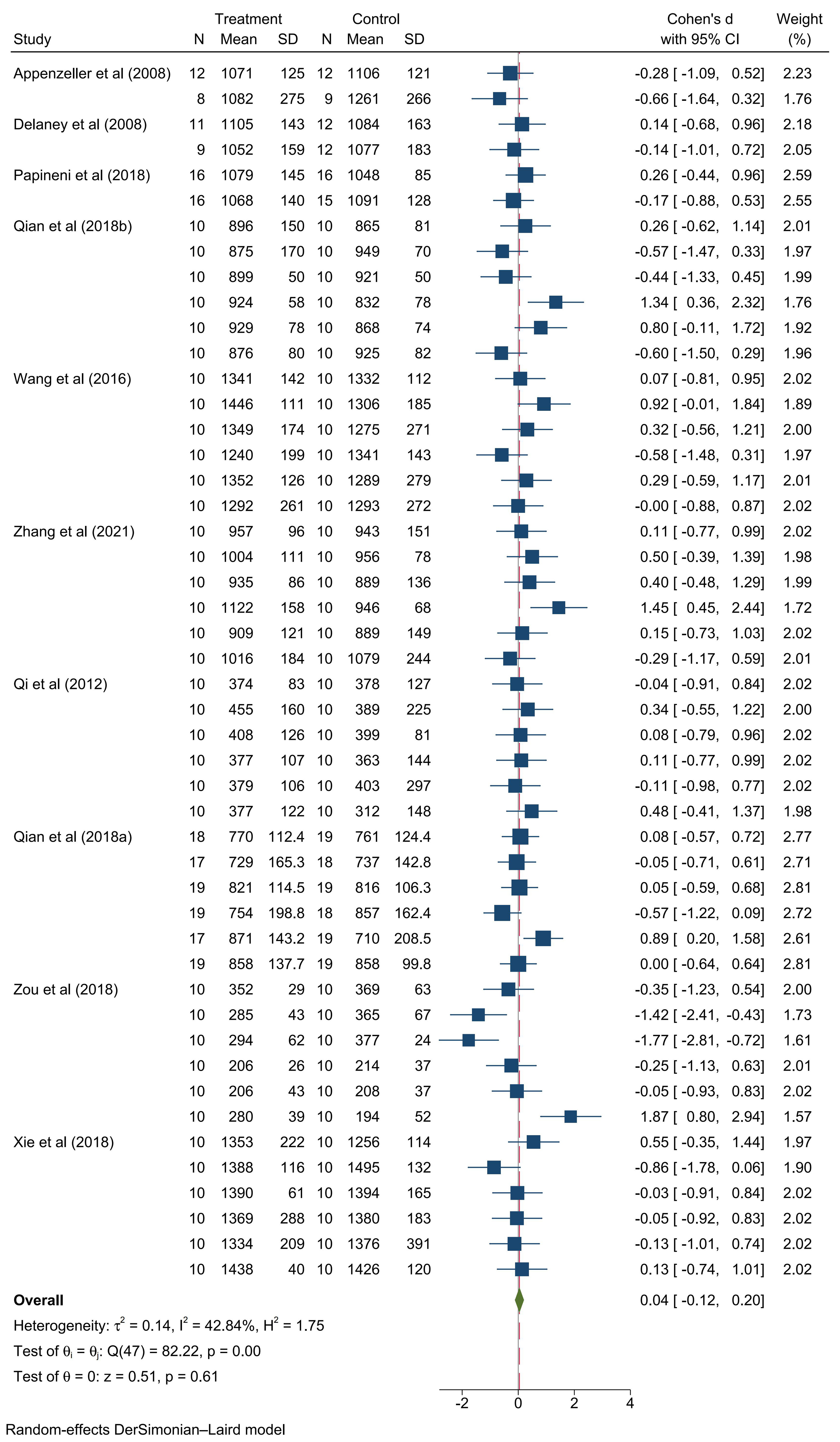


**Figure S74** Consuming GM maize showed no statistically significant impact on mammalian RBC concentration.


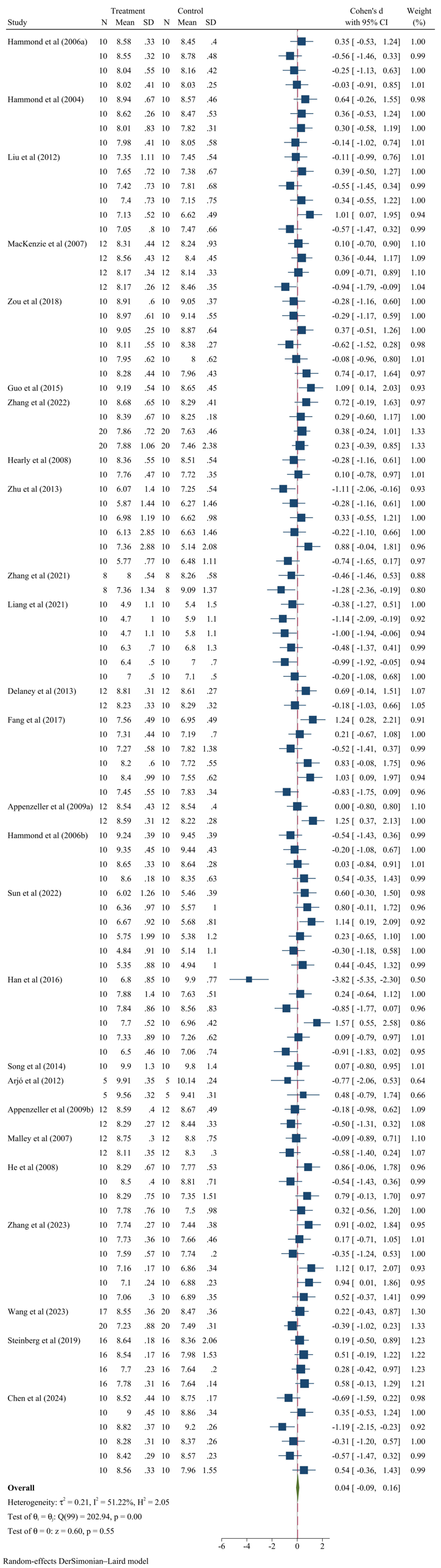


**Figure S75** Consuming GM rice led to statistically significant increase on mammalian RBC concentration.


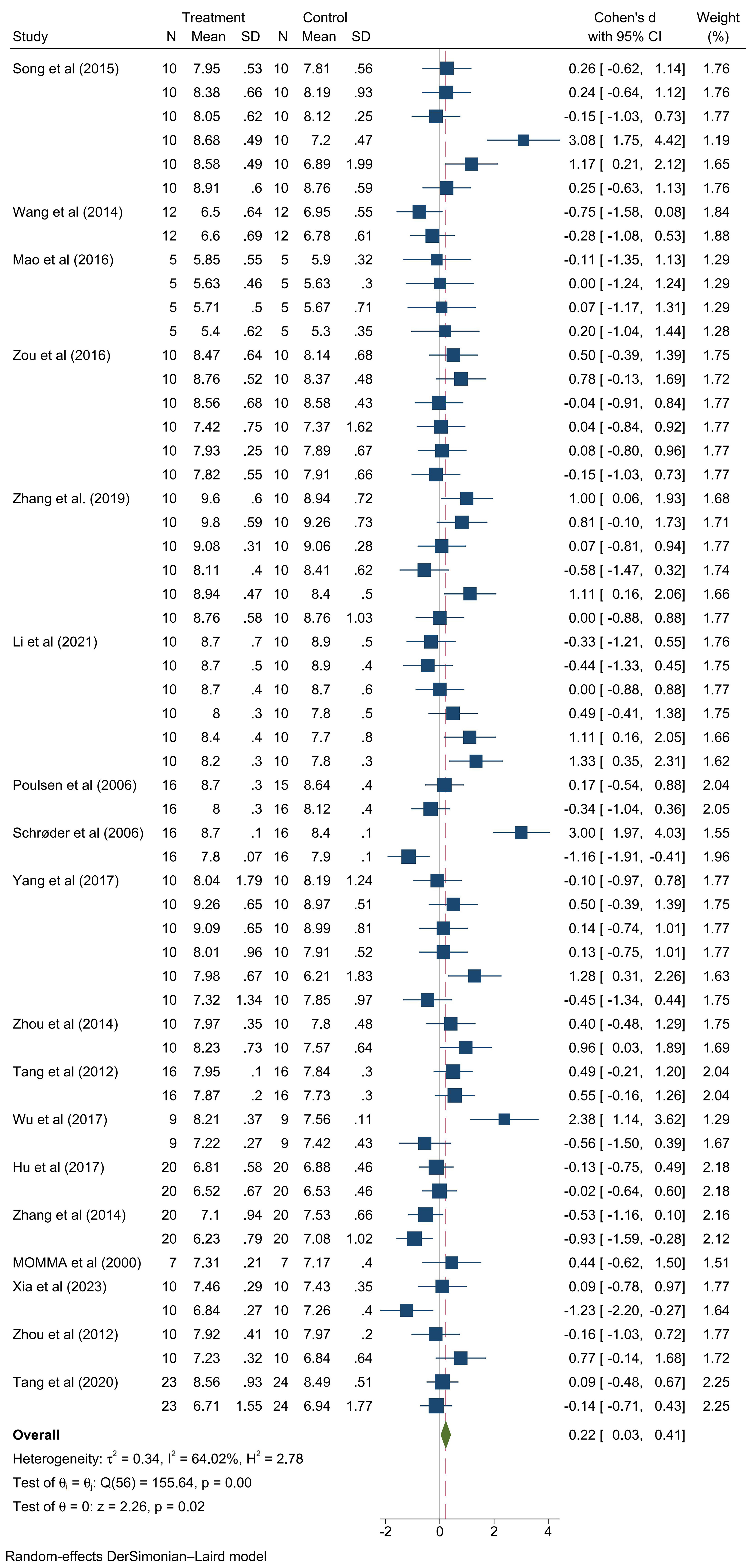


**Figure S76** Consuming GM soybean showed no statistically significant impact on mammalian RBC concentration.


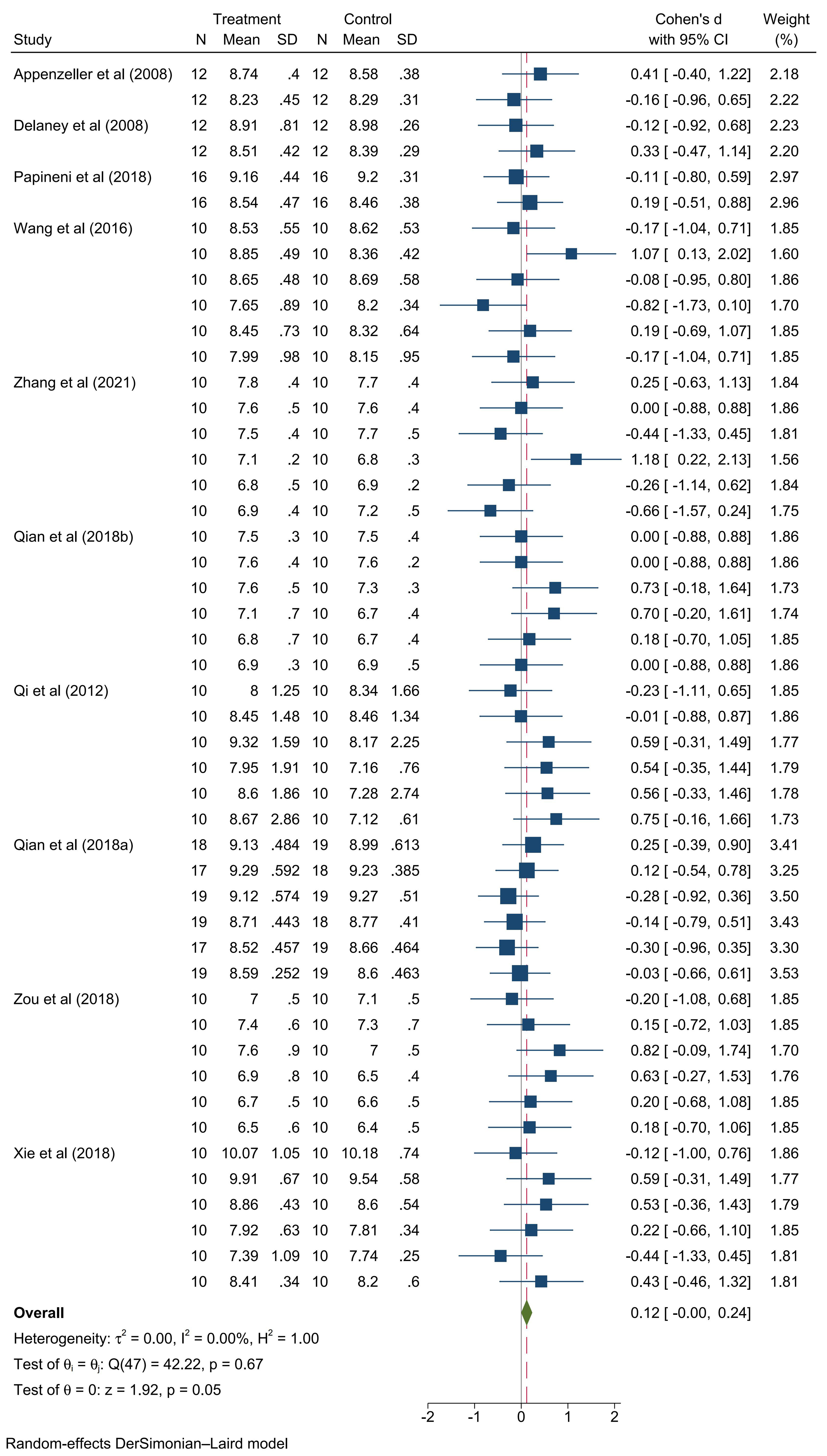


**Figure S77** Consuming low dose of GM rice showed no statistically significant impact on mammalian RBC concentration.


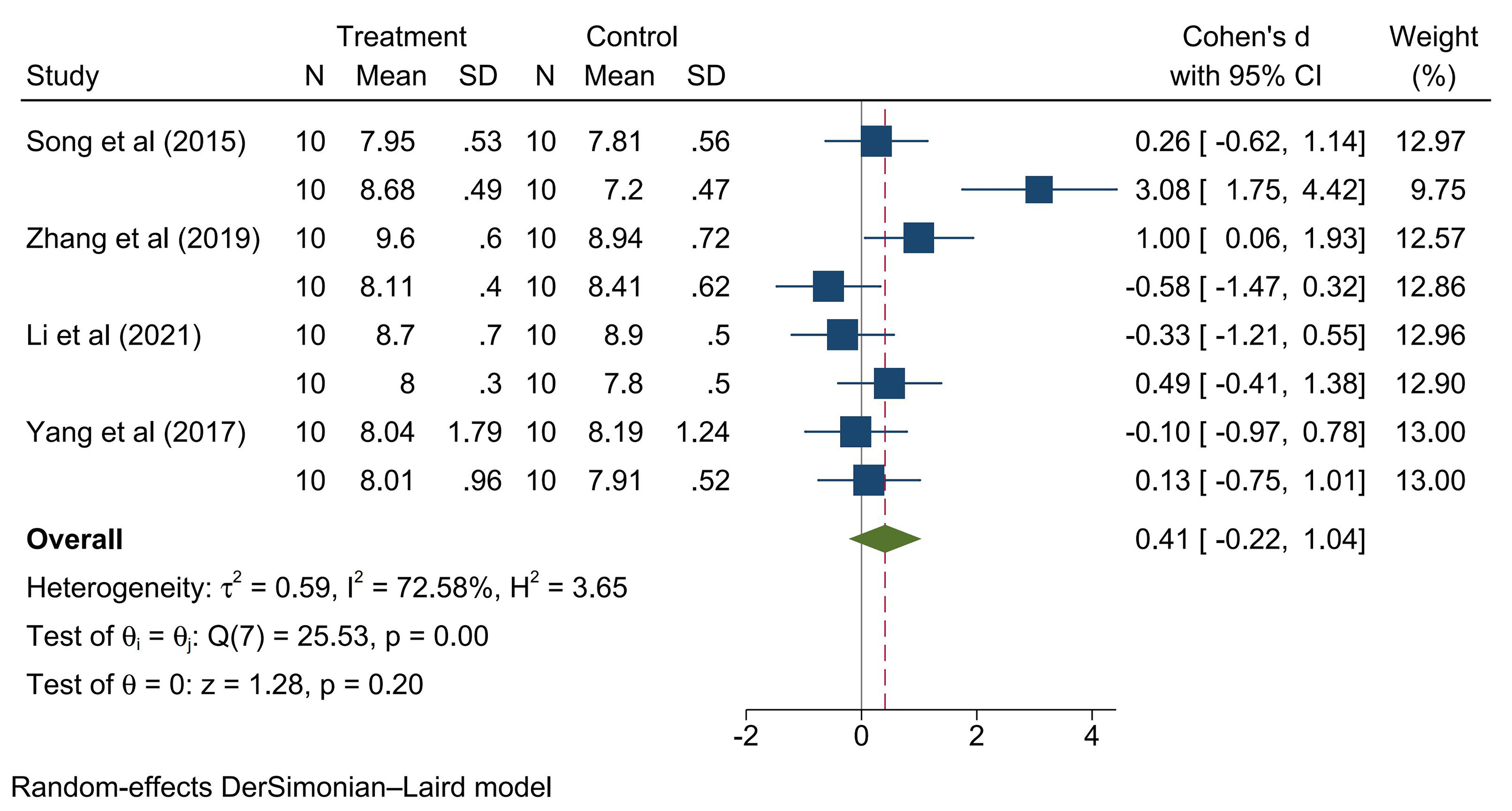


**Figure S78** Consuming medium dose of GM rice led to statistically significant increase on mammalian RBC concentration.


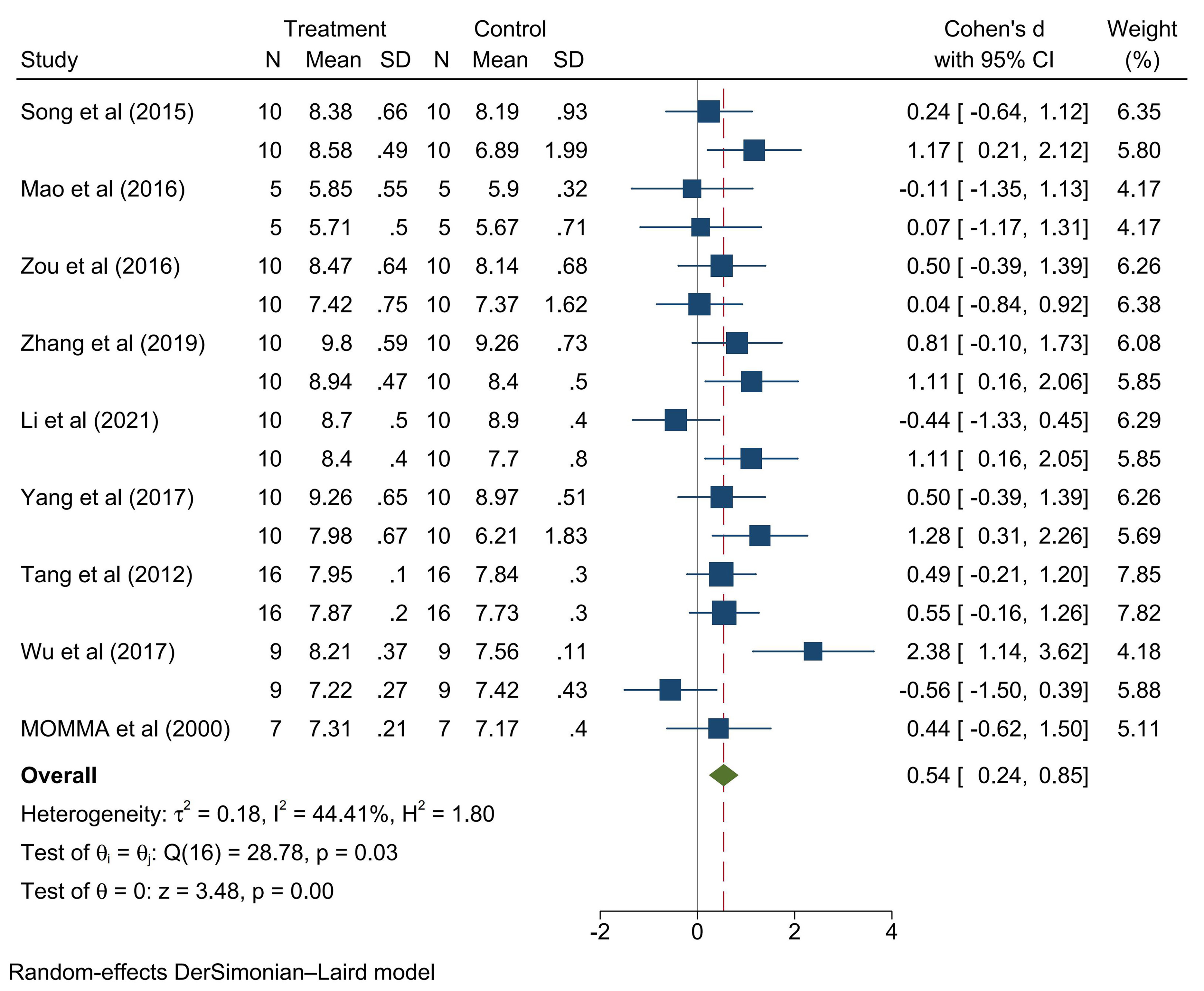


**Figure S79** Consuming high dose of GM rice showed no statistically significant impact on mammalian RBC concentration.

**
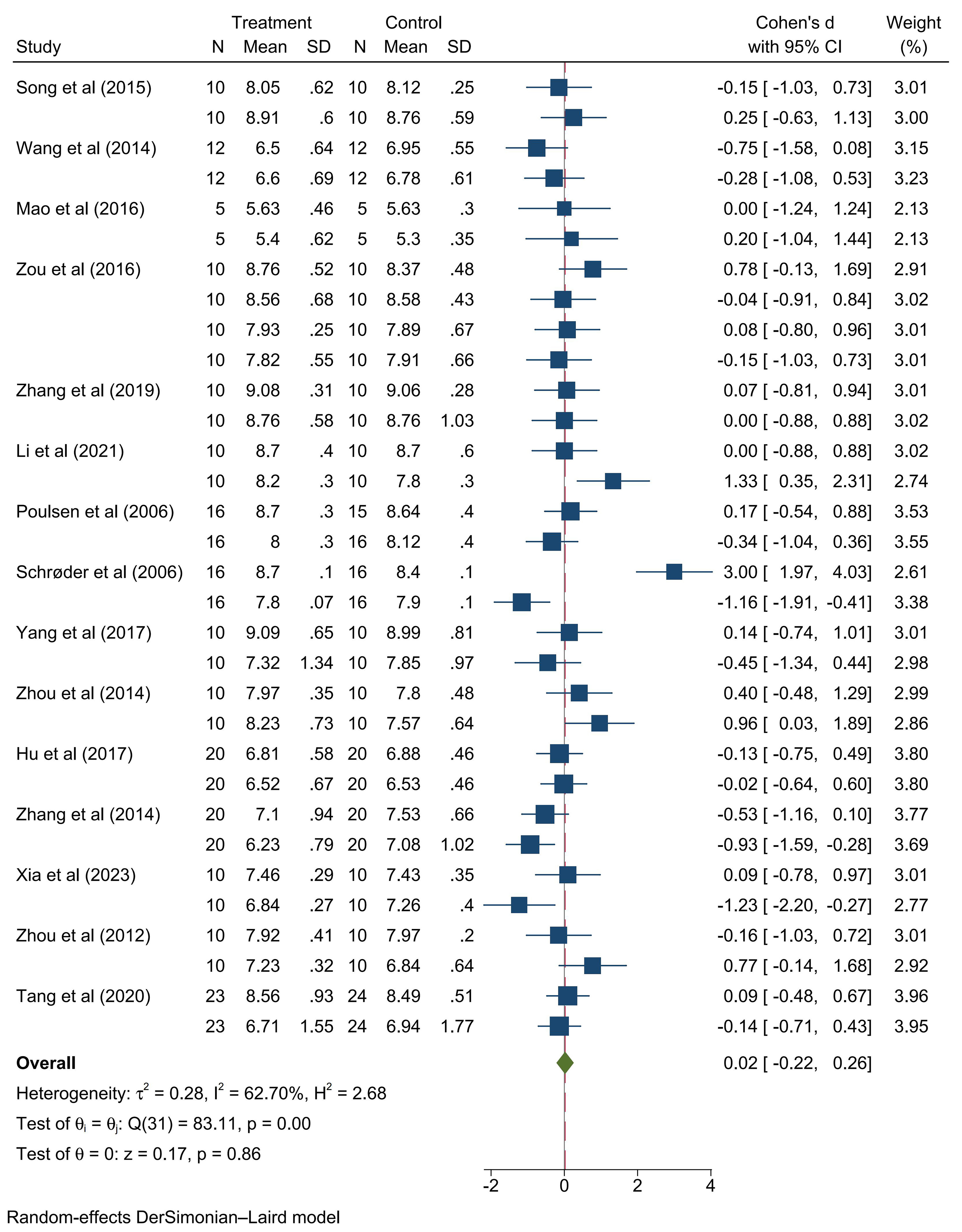
**
