# Supplementary material for: Comprehensive risk assessment revealed some physiological indicators responding to various GM-crop consumption
Source: GM Crops Food. 2025 Dec 19;17(1):2603726. doi: 10.1080/21645698.2025.2603726 (PMC12721096; doi:10.1080/21645698.2025.2603726)

**Indicators of renal function after GM-rice consumption**

**Figure S39** Consuming GM rice showed no statistically significant impact on mammalian GLU concentration.


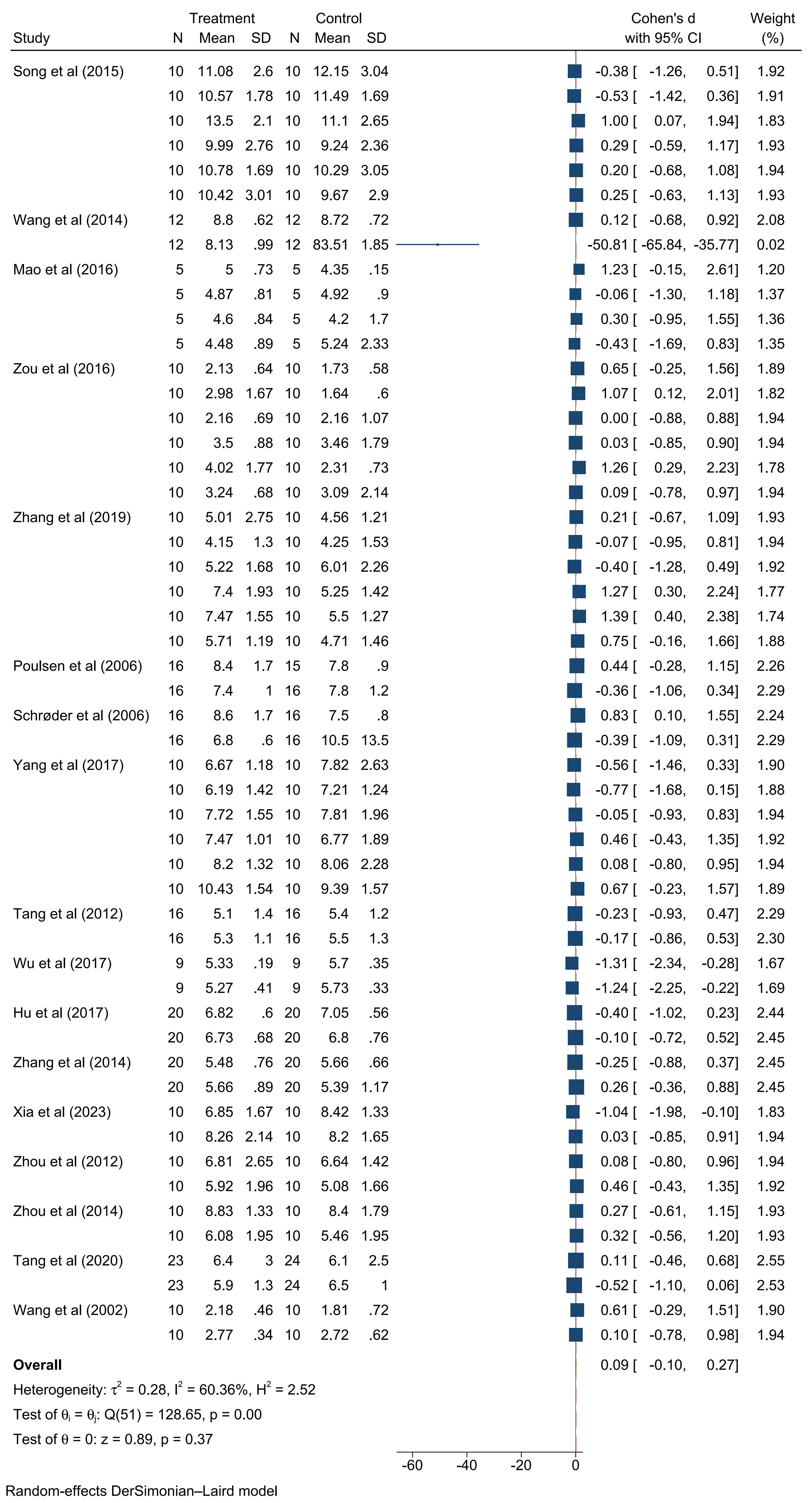


**Figure S40** Consuming GM rice showed no statistically significant impact on mammalian BUN concentration.


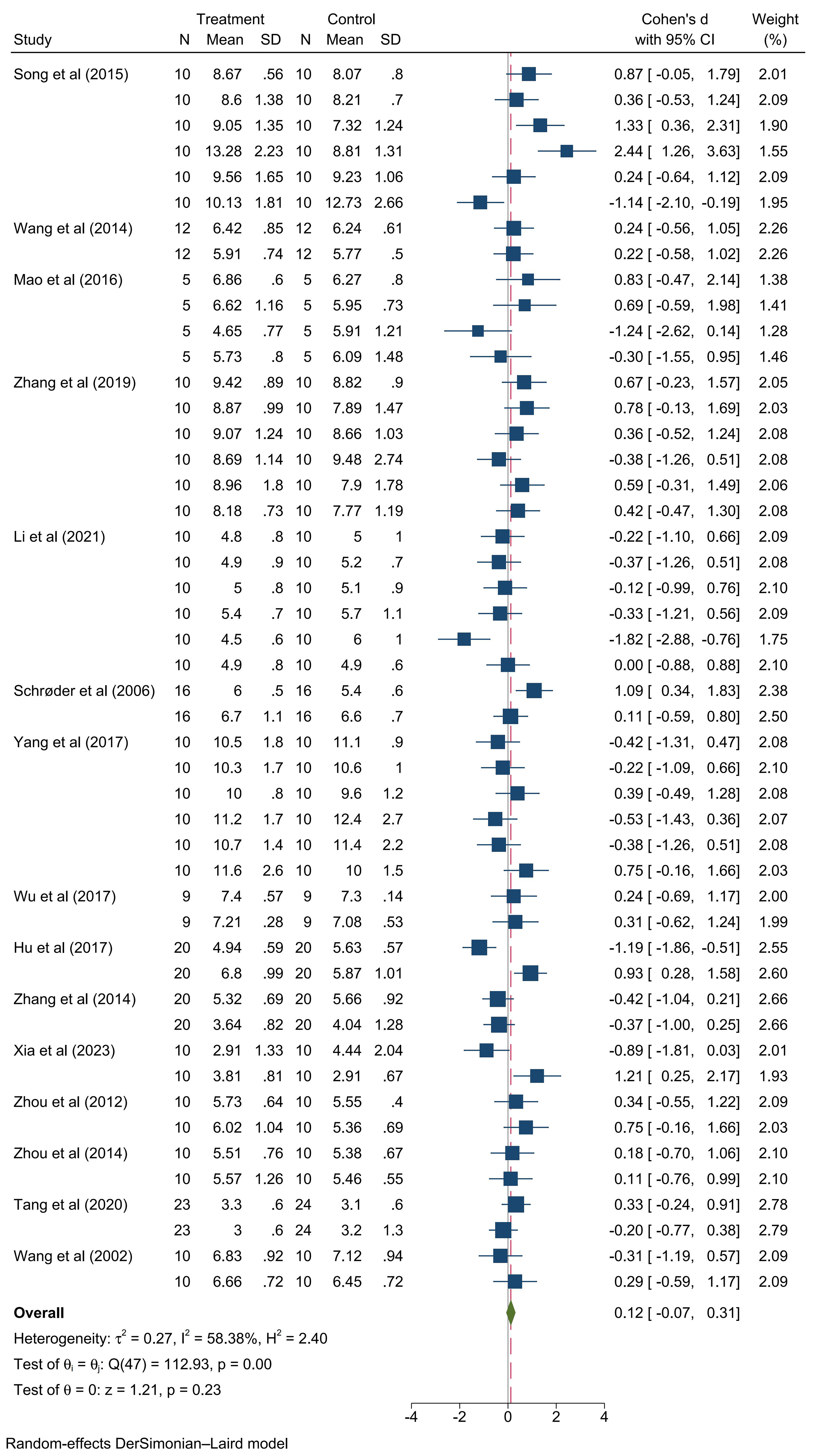


**Figure S41** Consuming GM rice showed no statistically significant impact on mammalian CRE concentration.


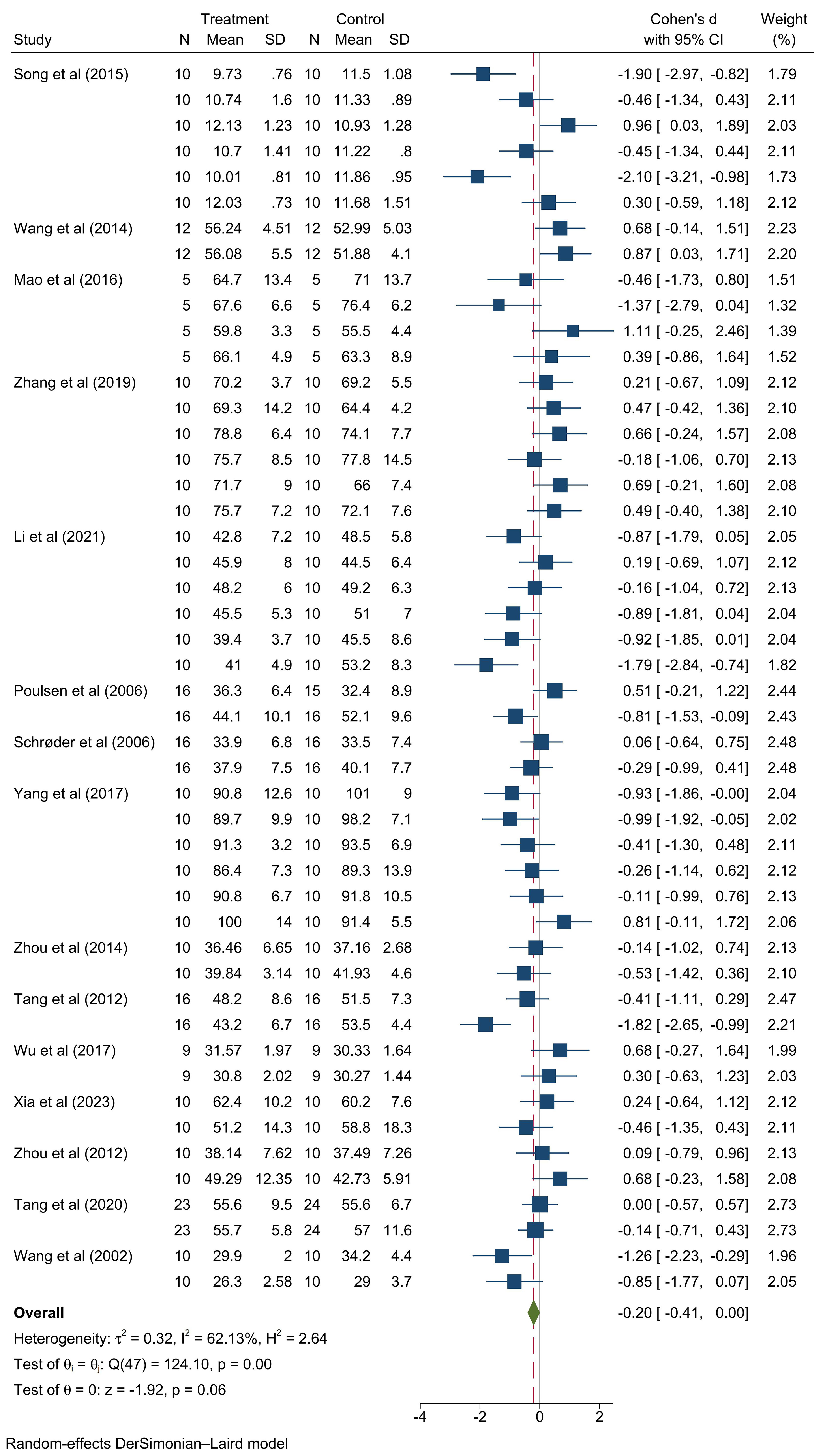

Supplement: Supplementary Figure S39 to S41.docx [file KGMC_A_2603726_SM6461.docx]
